# Effect of neuromuscular blocking agents on tracheal intubation quality in paediatric patients: a systematic review using network meta-analysis and meta-regression

**DOI:** 10.1016/j.bja.2025.08.036

**Published:** 2025-09-03

**Authors:** Luc E. Vanlinthout, Jacques J. Driessen, Robert Jan Stolker, Emmanuel M. Lesaffre, Johan M. Berghmans, Lonneke M. Staals

**Affiliations:** 1 Department of Anaesthesiology, Erasmus MC Sophia Children’s Hospital, University Medical Centre Rotterdam, Rotterdam, The Netherlands; 2 Interuniversity Institute for Biostatistics and statistical Bioinformatics, University of Leuven, Leuven, Belgium; 3 Department of Statistics and Actuarial Science, University of Stellenbosch, Stellenbosch, South Africa; 4 Department of Anaesthesiology and Perioperative Medicine, University of Ghent, Ghent, Belgium; 5 Department of Basic and Applied Medical Sciences, University of Ghent, Ghent, Belgium

**Keywords:** age-specific effects, comparative efficacy, drug interactions, haemodynamic responses, network meta-analysis, neuromuscular blocking agents, paediatric patients (0–12 yr), tracheal intubation

## Abstract

**Background:**

This meta-analysis is the first to compare tracheal intubation conditions and haemodynamic responses produced by various types and doses of neuromuscular blocking agents (NMBAs) in paediatric anaesthesia while also exploring factors associated with variability in outcomes.

**Methods:**

Randomised controlled and controlled clinical trials involving healthy paediatric participants (0–12 yr) were included. Trials compared intubation conditions using various NMBA interventions or NMBA-free settings under direct laryngoscopy. Outcomes included odds ratios (ORs) for excellent and acceptable intubation conditions, and mean differences for MAP and HR. Bayesian network, pairwise, and cumulative meta-analyses, along with meta-regression, assessed NMBA effectiveness and covariate effects.

**Results:**

Data from 105 trials (8008 participants) were analysed. Suxamethonium ≥1.50 mg kg^−1^ and rocuronium ≥0.90 mg kg^−1^ provided similar intubation conditions, though not consistently within 60 s. Other NMBAs were, on average, slower and less effective. Opioids decreased MAP and HR but did not improve intubation conditions when combined with suxamethonium ≥1.00 mg kg^−1^ or rocuronium ≥0.90 mg kg^−1^. Non depolarising NMBAs enhanced excellent (OR: 2.97 [1.82–5.10]) and acceptable intubation conditions (OR: 2.29 [1.14–4.39]) more in younger children (1.64 [1.08–2.20] yr) than in older ones (5.53 [4.04–7.01] yr). Intubation without NMBAs was most difficult in neonates and infants, with conditions improving until about age 4 yr. Beyond this, the difference in intubation quality between groups with and without NMBAs increased with age, indicating a greater benefit of using NMBAs in older children. Values are mean (95% credible interval).

**Conclusions:**

We present a meta-analytical approach to synthesise and consolidate evidence from previous research and demonstrate how *neuromuscular blocking agent* type and dose, intubation timing, age, and induction drugs affect the safety and efficacy of paediatric airway management. Low-to-moderate confidence can be assigned to the recommendations from this meta-analysis.

**Systematic review protocol:**

PROSPERO (CRD42018097146).

**Editor's key points**

• Although neuromuscular blocking agents (NMBAs) improve intubating conditions in paediatric patients, uncertainty remains about the best choice and how covariates such as age, anaesthesia technique, and intubation timing influence outcomes.
• In this meta-analysis, the authors compare commonly used NMBA interventions, rank their effectiveness, and provide age-stratified and anaesthesia-specific efficacy estimates.
• The findings provide an opportunity to refine recommendations on NMBA use for paediatric intubation. The authors call for further high-quality research given the low-to-moderate quality of the available evidence and scarcity of videolaryngoscopy studies.

Tracheal intubation in children during the induction of anaesthesia is conducted to ensure a safe and controlled airway throughout surgical or diagnostic interventions. This should enable controlled ventilation, prevent aspiration, and allow the safe administration of inhalation anaesthesia during the maintenance of general anaesthesia.^1^

Neuromuscular blocking agents (NMBAs) are commonly used in adults to facilitate laryngoscopy and intubation.^2^, ^3^, ^4^ However, in paediatric anaesthesia, the approach may differ, particularly when neuromuscular block is not warranted for the surgery. In these patients, intubation is sometimes conducted without NMBAs.^5^, ^6^, ^7^, ^8^

When tracheal intubation is conducted at inadequate depth of anaesthesia without neuromuscular block, airway reflexes are enhanced, which can pose several problems. These encompass an increased risk of laryngospasm, involuntary movements, airway trauma, oesophageal intubation, and hypoxaemia caused by repeated failed intubation attempts.^9^, ^10^, ^11^, ^12^, ^13^, ^14^ Multiple attempts can induce airway oedema or bleeding, decreasing the chances of success in subsequent attempts.^11^^,^^15^^,^^16^ The high metabolic rate, high cardiac output, and small functional residual capacity of these patients predispose them to the rapid development of oxygen desaturation during apnoea.^2^^,^^17^, ^18^, ^19^

Additionally, anatomical features in younger children, such as a larger occiput, higher placed larynx, proportionally larger tongue, and an elongated epiglottis can complicate optimal alignment, rendering both direct laryngoscopy and tracheal tube insertion more challenging.^18^, ^19^, ^20^ The use of neuromuscular block limits patient movement, reduces airway reflexes, and improves laryngeal exposure, which can potentially improve the quality of intubation and reduce the associated morbidity.^4^^,^^14^^,^^21^, ^22^, ^23^, ^24^

A wide range of NMBAs are available to facilitate tracheal intubation in children in different surgical contexts, including elective and emergency procedures.^25^ To date, intubation quality and haemodynamic effects of different types and doses of NMBAs in children have not been comprehensively analysed in a quantitative review. This systematic review and meta-analysis were conducted to compare intubation conditions and the haemodynamic effects produced by commonly used NMBA treatments and induction protocols. Furthermore, it aimed to identify factors contributing to variability in these outcomes and to quantify their impact on intubation quality and haemodynamic responses.

## Methods

### Research protocol

A systematic review was conducted, incorporating both network and pairwise meta-analyses.^26^ This review followed a registered protocol (PROSPERO registration number: CRD42018097146) and adhered to the guidelines outlined in the Cochrane Handbook for Systematic Reviews^27^ and in the PRISMA Extension Statement for Reporting of Systematic Reviews that include Network Meta-analyses of Healthcare Interventions(Supplementary material 1).^28^

### Search strategy and selection criteria

Based on the Population, Interventions, Comparators, Outcomes, and Study design (PICOS) framework, relevant Medical Subject Headings (MeSH) terms were identified from a preliminary screening. With this approach, all potential studies satisfying the specific criteria described here below could be identified.

We examined randomised (RCTs) and controlled clinical trials (CCTs) published in seven databases: PubMed/MEDLINE, Excerpta Medica Database (Embase), Web of Science, Scopus, Cochrane Central Register of Controlled Trials, Scientific Electronic Library Online (SciELO), and the China National Knowledge Infrastructure (CNKI) (Supplementary material 2). To search for additional relevant studies that were not gathered by the electronic search, we manually reviewed reference lists of the retrieved trials.

The eligible trials were those published as peer-reviewed full-text articles or abstracts. Doctoral dissertations, case reports, animal studies, letters, reviews, and trials that were not peer reviewed were excluded. For studies published more than once (duplicates), we only considered the reports with the most informative and complete data.^29^

We included RCTs and CCTs that enrolled healthy participants aged 0–12 yr, with ASA physical status 1 and 2, in any language without limits on the publication date.^29^ Studies that included a minority of ASA 3 patients were considered, and the effect of their inclusion on the results was evaluated via a sensitivity analysis.

The participants in the included trials were undergoing elective or emergency surgery requiring tracheal intubation. We searched for studies that compared intubation conditions and haemodynamic responses after induction sequences involving different types and doses of NMBAs, both relative to one another and to protocols in which no NMBA was administered. The NMBAs considered included atracurium, cisatracurium, mivacurium, rocuronium, suxamethonium, and vecuronium. We accepted all trials in which anaesthesia was induced using inhalation or i.v. techniques with or without opioids.

As most studies evaluating the impact of NMBAs on tracheal intubation have used direct laryngoscopy, this analysis was restricted to trials that conducted airway management under direct laryngoscopy.

Trials that enrolled preterm neonates (gestational age <37 weeks at birth) and participants with known psychiatric, neurological, neuromuscular, hepatic, renal, and endocrine diseases including trials in which NMBAs were administered by any route other than i.v. were excluded.

### Intervention effect (primary, secondary, and other outcomes)

Intervention/treatment was defined as the administration of a single dose of a specific NMBA to induce neuromuscular block in healthy paediatric participants aged 0–12 yr. The single-bolus method is generally considered as the ‘gold standard’ for measuring the dose–response relationship of NMBAs and appears to be the most common in paediatric clinical practice.^30^^,^^31^

We assessed intubation conditions using the Goldberg scale,^32^ a commonly used composite tool that evaluates ease of laryngoscopy, vocal cord position, and patient response to intubation. Each is rated on a three-point scale, providing a comprehensive score of intubation quality. When not directly reported, study outcomes were converted to this scale if sufficient details were available. The primary outcome was ‘excellent intubation conditions’ which was defined by three criteria: easy tube insertion, immobile open vocal cords, and no patient movement during laryngoscopy and tracheal intubation.^30^^,^^32^ With the complete absence of muscular activity, excellent intubation conditions can be considered as a hard endpoint in which interpretation bias could be avoided.^33^ The secondary outcome was ‘clinically acceptable intubation conditions’ in which the score for each of the three items, that is, ease of intubation, vocal cord movement, and patient response to intubation, was graded either excellent or good.^32^ Other outcomes included unacceptable conditions, failed first pass intubation attempts, the time to laryngoscopy, intubation, or both, MAP (mm Hg), and HR (beats min^−1^) assessed immediately before, and at 1 and 3 min after intubation.

In each study arm, intubation outcomes were analysed using two categories: excellent conditions alone, and acceptable conditions, which included both excellent and good intubation conditions.^32^ For each category, either excellent or acceptable intubation conditions, the proportion of successful events was calculated by dividing the number of participants meeting the criterion (denoted as n) by the total number in the arm (denoted as t), yielding n/t. Odds were calculated by dividing the number of successes (n) by the number of failures (t – n), expressed as n/(t – n). Odds ratios (ORs) were used to compare the odds of an outcome occurring in one treatment arm with the odds of an outcome occurring in another.^34^ For haemodynamic parameters, mean differences were used as the measure of effect.

Studies with multiple arms were included by dividing the ‘shared’ group into two or more groups with smaller sample sizes.^35^ For example, when comparing one control group with two intervention groups (e.g. treatment A *vs* treatment C and treatment B *vs* treatment C), the sample size and event count of the control group (C) were divided equally between the two comparisons to avoid double-counting.

### Risk of bias and quality assessment

The Risk of Bias in Non-Randomized Studies of Interventions (ROBINS-I) risk of bias tool was used to evaluate the quality of evidence and risk of bias in each of the included CCTs.^36^ The evaluation of the risk of bias in the RCTs was evaluated using the Risk-of-Bias 2 (RoB-2) tool.^37^ Furthermore, we reviewed the reporting of sample size calculations. Credibility of results of the meta-analyses was evaluated using the Confidence In Network Meta-Analysis (CINeMA) framework.^38^

### Data management and extraction

EndNote X9 citation management software (EndNote™ (Clarivate Plc, London, UK)) was used to store references, remove duplicates, and facilitate the initial screening process. Two authors (LEV and JMB) independently selected the studies, assessed the risk of bias, rated the quality of evidence, and extracted data. Disagreements were resolved via a consensus. If consensus could not be achieved, a third author (JJD) made the final decision.

### Data synthesis

Different meta-analyses were conducted based on the nature of the evidence available in the primary studies. Moreover, conducting multiple meta-analyses on the same topic can improve the clarity and consistency of the available evidence, thereby refining treatment options, especially in paediatric studies.^39^ This approach helps address the complexities of paediatric reviews, including variations in outcomes across different paediatric age groups.^40^

A. In the first type of meta-analysis, the proportions of excellent and acceptable intubation conditions were collated across multiple nodes (treatment groups)—each comprising similar participants who received a specific NMBA or NMBA-free intervention—using network meta-analysis. This was conducted to perform a comparative outcome analysis of the interventions and rank them according to their efficacy in facilitating tracheal intubation (Part 1: main model).
B. The second type of meta-analysis focuses on how patient-related factors (e.g. age) and procedural variables (e.g. time to intubation, induction agents, NMBA-free approaches) influence intubation quality and haemodynamic responses (Part 2: subgroup analyses).

### Network meta-analysis

Network meta-analysis is a technique for juxtaposing and collating three or more interventions simultaneously in a single analysis. This is accomplished by combining both direct (from head-to-head trials) and indirect evidence across a network of treatment comparisons.^27^ The network diagram provides an accessible format for describing evidence, how information flows indirectly, and whether there is comparator preference or if a given comparison is over- or under-represented.^26^, ^27^, ^28^ To describe network structure, several metrics with distinct properties are available.^41^

We conducted a contrast-based random effects network meta-analysis with Bayesian software using Markov Chain Monte Carlo computations. We assumed common between-study variance (τ^2^). Vague prior distributions, that is, normal distributions with a large variance, were selected for all the coefficients and treatment contrasts.^42^ We created funnel plots and tested their asymmetry to explore potential publication bias, small-study effects, or both.^43^ The treatments were ranked according to their surface under the cumulative ranking curve (SUCRA).^44^

The validity of a network meta-analysis relies on the assumptions of transitivity and coherence.^26^, ^27^, ^28^^,^^45^ (In)transitivity was evaluated by checking the distribution of effect modifiers across treatment nodes. Global inconsistency was evaluated using residual deviance, indicating model fit; high values indicate potential inconsistency within the network. Local inconsistency within closed loops was assessed using the node-splitting method.^45^

### Pairwise and cumulative meta-analysis

We conducted pairwise meta-analysis, cumulative meta-analysis, or both to evaluate the effect of age, timing of intubation, and anaesthesia technique on the creation of excellent or acceptable intubation conditions. Trial sequential analysis was conducted to determine whether the size of information (i.e. the number of participants) available for the pairwise and cumulative meta-analyses was sufficient to detect or reject a particular intervention effect.^46^

### Meta-regression and subgroup analysis

Subgroup analyses and meta-regression were used to explore potential patient-related or procedural factors for their association with the variability in intubation outcomes.^26^, ^27^, ^28^^,^^47^, ^48^, ^49^ Meta-regression examined age (yr), weight (kg), intubation time (min), use of opioids, inhalation agents (yes/no), or both, and publication year (year) as potential effect modifiers.

### Comparing timing of intubation between neuromuscular blocking agent treatments

Intubation times across NMBA treatments were assessed using Bayesian one-way anova, with *post hoc* pairwise comparisons estimating the probability of differences between groups.^50^

### Sensitivity analysis

Sensitivity analyses were conducted to assess the robustness of our findings by examining the impact of studies with high risk of bias, nonrandomised designs, limited inclusion of ASA 3 patients, and those published before the year 2000. These study-level characteristics were included as binary covariates (1/0) in a Bayesian network meta-regression model, with their influence assessed via posterior estimates.

### Software

The free available R programming language version 4.4.2 (The R Foundation for Statistical Computing, Vienna, Austria) was used for statistical computing and graphs.

Bayesian network meta-analysis, comparative effectiveness ranking, consistency analysis, assessment of publication bias, and network meta-regression were conducted using the R package ‘Generate Mixed Treatment Comparison (GeMTC)’, version 1.0–2 (GitHub. R software ‘GeMTC’ package; freely available from https://github.com/gertvv/gemtc).^51^^,^^52^ Evaluation of confidence in the results obtained from network meta-analysis was performed using the ‘CINeMA’ web application (Institute of Social and Preventive Medicine, University of Bern, Bern, Switzerland; freely available from CINeMA unibe.ch).^38^ Pairwise and cumulative meta-analysis along with meta-regression were performed within the Bayesian framework using the R package ‘Bayesmeta’, version 3,3 (GitHub. R software ‘Bayesmeta’ package; freely available from https://gitlab.gwdg.de/croever/bayesmeta).^53^^,^^54^ Trial Sequential Analysis was performed using ‘Trial Sequential Analyzer’, version 0.9.5.9 Beta (Copenhagen Trial Unit, Centre for Clinical Intervention Research, Copenhagen, Denmark; freely available from https://ctu.dk/tsa/).^46^

## Results

### Study selection and participant characteristics

**Fig 1:**
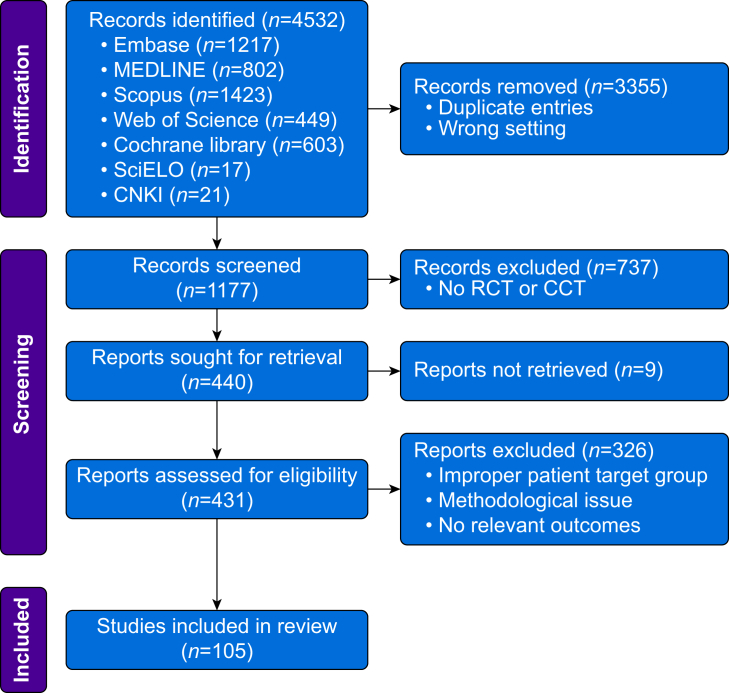
Flowchart of retrieved, excluded, and analysed trials. CCT, controlled clinical trial; CNKI, China National Knowledge Infrastructure.

Electronic searches yielded 4532 potentially relevant trials. Among these, 105 eligible research papers were identified, comprising 94 RCTs and 11 CCTs (Fig. 1). These papers encompassed 8008 paediatric participants, mostly classified as ASA 1–2, across 315 study arms (Supplementary material 3). Notably, three RCTs enrolled a few ASA 3 patients.^55^, ^56^, ^57^

Participants in the incorporated trials were undergoing elective or emergency surgery requiring tracheal intubation and were recruited over an approximately 39-yr period from January 1986 to November 2024, with 28% of them enrolled before 2000, mainly in studies involving atracurium, vecuronium, or suxamethonium (Supplementary material 4), The average (range) of the reported mean ages of the study groups was 5.30 (0.05-11.20) yr with 95% of the study population being younger than 8.80 yr. Participants aged ≤1 yr accounted for 8.50% of the population, whereas those aged ≤2 yr comprised 11.58%. The majority (88.41%) of study arms had an average age ranging between 2 and 12 yr (Supplementary material 5).

In the studies included, the pattern of average age–weight data closely aligns with the known growth standards of the WHO and the Centres for Disease Control and Prevention (Supplementary material 5).

### Study characteristics

Given the risk of bias assessments, ROBINS-I for CCTs^36^ and RoB-2 for RCTs^37^ (Supplementary material 6), many trials lacked details on sequence generation and allocation concealment, with only 36 of 105 (34.3%) calculating the required sample sizes before starting the investigation. These issues indicate that adherence to the Consolidated Standards of Reporting Trials (CONSORT) guidelines^58^ is not always consistent.

Out of 105 studies, 45 (42.9%) reported the intubator’s experience or professional rank, usually as staff anaesthetists. Neuromuscular transmission before intubation was monitored in 62/105 (59.0%) trials (Supplementary material 3).

Based on the nature of the evidence available in the primary studies, different meta-analyses were conducted, each targeting a specific research objective. In the first type of meta-analyses, intubation conditions were compared between specific interventions. The second type of meta-analyses focuses on how various patient-related (age) or procedural variables (timing of airway management, anaesthesia technique, use of specific NMBAs, or NMBA-free approaches) can affect the ease of tracheal tube insertion.

### Part 1: comparative outcome analysis and timing of intubation (main model)

In the first part, different treatment options were juxtaposed, collated, and ranked according to their efficacy, that is, the proportion of excellent and acceptable intubation conditions they were able to produce. This was conducted using a network meta-analysis.

**Fig 2:**
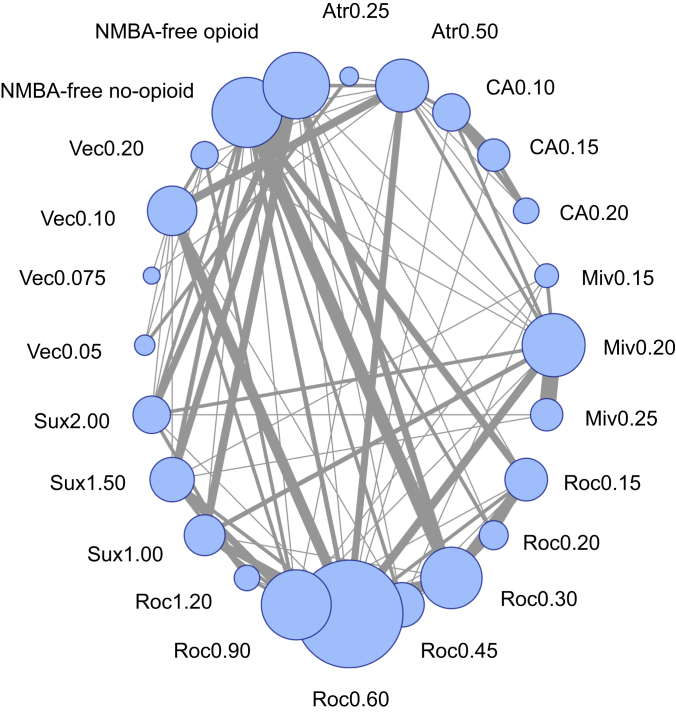
A network plot of treatments, involving neuromuscular blocking agents (NMBAs) and NMBA-free interventions, illustrates the relationships within the network of treatment comparisons. In this diagram, each node represents an intervention, whereas each edge indicates a direct comparison between two interventions. The amount of evidence is reflected in the weighting of nodes and edges: node size corresponds to the number of studies evaluating each intervention, and edge thickness represents the number of direct comparisons. NMBA treatments are labelled according to the NMBA selected and the dose (mg kg^−1^) administered. The following NMBAs are used in the specific treatments: atracurium (Atr), cisatracurium (CA), mivacurium (Miv), rocuronium (Roc), suxamethonium (Sux), and vecuronium (Vec). The NMBA-free interventions were aggregated into nodes labelled according to whether opioids were used (opioid) or not (no opioid).

For the comparative outcome analysis of the intubation conditions, a 24-node treatment network was produced from 95 studies (85 RCTs and 10 CCTs) with 280 study arms, including 7263 paediatric participants (Supplementary material 3). The resulting treatment network included 204 direct comparisons between pairs of interventions (nodes) (Fig. 2). Certain treatment comparisons were informed by several trials, whereas others were sparsely represented (either by direct or indirect evidence). In 158/204 (77.5%) of direct comparisons, more than one study was involved. From the 276 direct pairwise combinations that were possible with 24 treatments (C[24,2] = 23∙24/2=276), only 105 (38.0%) different treatment comparisons could be determined. No evidence of intransitivity, local inconsistency, potential small-study effects, or publication bias could be demonstrated (Supplementary material 7).

**Fig 3:**
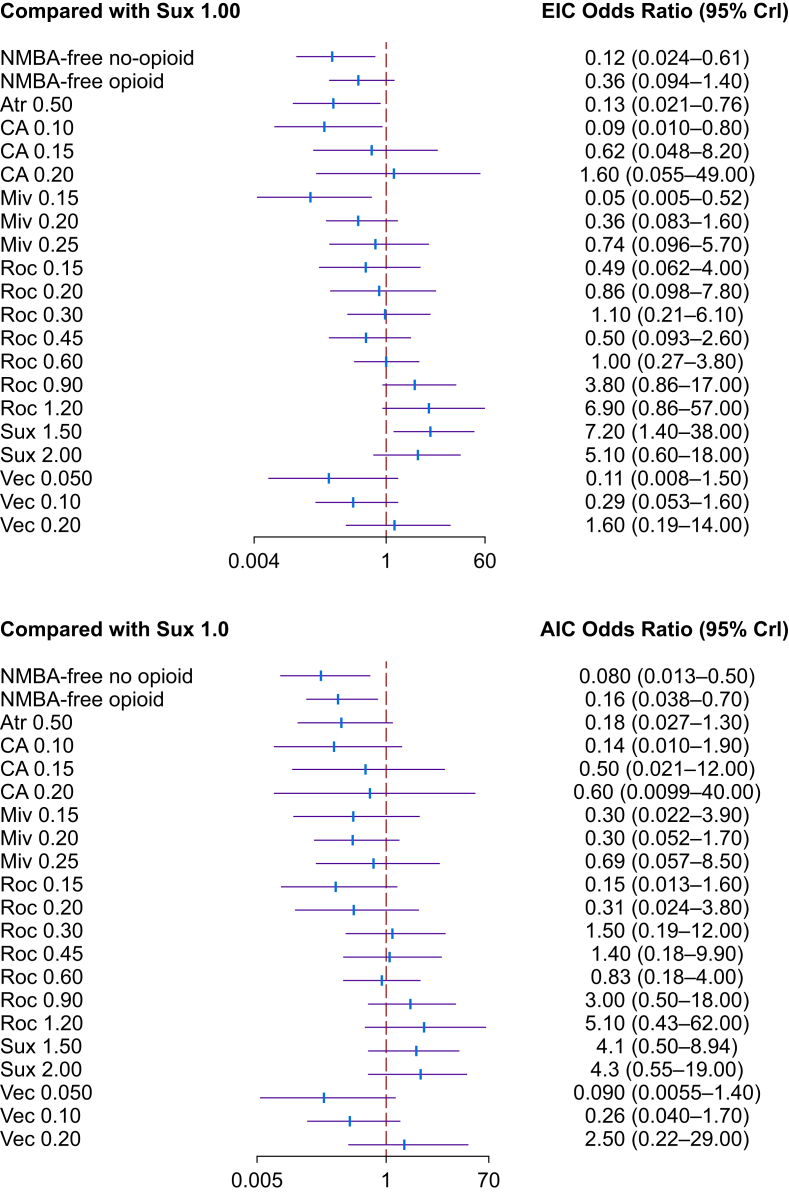
Forest plots representing the relative effectiveness estimates and their 95% credible intervals (95% CrI) for specific neuromuscular blocking agents (NMBAs) and NMBA-free treatments. The upper panel presents odds ratios (mean [95% CrI]) for achieving excellent intubation conditions (EIC), whereas the lower panel shows odds ratios (mean [95% CrI]) for acceptable conditions (AIC), both in relation to the reference treatment of suxamethonium at 1.00 mg kg^−1^ (marked by the red vertical dotted line at odds ratio =1). Odds ratios less than, equal to, or greater than 1 indicate treatment effects that are smaller, the same, or larger than those produced by suxamethonium 1.00 mg kg^−1^, respectively. Each NMBA treatment is labelled according to the specific NMBA used and the administered dose (mg kg^−1^). The NMBAs analysed include atracurium (Atr), cisatracurium (CA), mivacurium (Miv), rocuronium (Roc), suxamethonium (Sux), and vecuronium (Vec). The NMBA-free interventions are categorised based on whether opioids were administered (opioid) or not (no opioid).

The forest plots (Fig. 3) represent the average efficacy estimates along with their 95% credible interval (95% CrI) for both excellent and acceptable intubation conditions. The relatively narrow CrIs for most estimates suggest that imprecision is unlikely to be a major concern. However, some interventions—especially those involving cisatracurium—had wider intervals, likely because of inconsistent intubation timing, limited comparative data, or both (Supplementary material 3).

Supplementary material 8 presents the comparative performance of NMBA treatments to produce excellent or acceptable intubation conditions. Based on SUCRA scores, suxamethonium ≥1.50 mg kg^−1^ and rocuronium ≥0.90 mg kg^−1^ ranked highest, whereas the remaining interventions, on average, demonstrated lower efficacy. Reduced NMBA dosing and NMBA-free induction were generally associated with inferior intubation quality.

**Fig 4:**
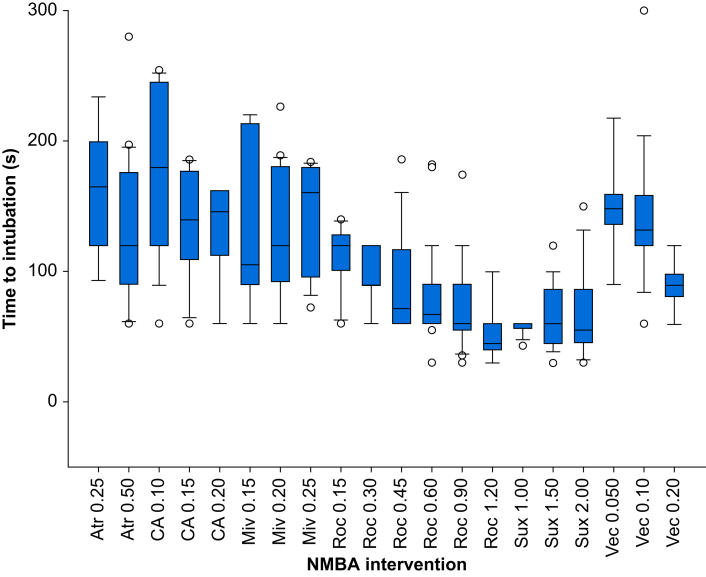
A diagram displaying the intubation times after specific neuromuscular blocking agent treatments, represented as box and whisker plots to illustrate the distribution and variability of the data. Atr, atracurium; CA, cisatracurium; Miv, mivacurium; NMBA, neuromuscular blocking agent; Roc, rocuronium (Roc); Sux, suxamethonium; Vec, vecuronium.

The times to intubation (median [IQR]) are shown in Figure 4. A Bayesian one-way anova with *post hoc* pairwise comparisons reveals significant differences in intubation times among the NMBA treatments evaluated. The shortest intubation times were measured for intubations facilitated with suxamethonium ≥1.00 mg kg^−1^ and rocuronium ≥0.90 mg kg^−1^ (Supplementary material 9).

Adding covariates to the network meta-analysis model resulted in only minor changes in model fit indicators, such as the deviance information criterion, total residual deviance, and the 95% CrI for treatment effect estimates. Consequently, the inclusion of these covariates did not improve the network meta-analysis model’s ability to accurately explain treatment effects.

### Part 2: subgroup analysis

In this second type of meta-analysis, we assessed how participant- and procedure-related variables influenced intubation quality or haemodynamic parameters.

Longer times to intubation significantly improved both excellent (OR: 5.64 [2.23–5.03]) and acceptable intubation conditions (OR: 5.00 [2.03–2.43]). Inhalation anaesthetics enhanced excellent intubation conditions with NMBAs (OR: 2.86 [1.20–7.24]) but did not significantly affect acceptable conditions (OR: 0.84 [0.47–1.52]). No significant differences in excellent or acceptable intubation conditions scores were found between suxamethonium ≥1.00 mg kg^−1^ and rocuronium ≥0.90 mg kg^−1^, with OR changes of 1.57 (0.06–37.34) for excellent and 1.79 (0.91–3.00) for acceptable intubation conditions (Supplementary material 10).

**Fig 5:**
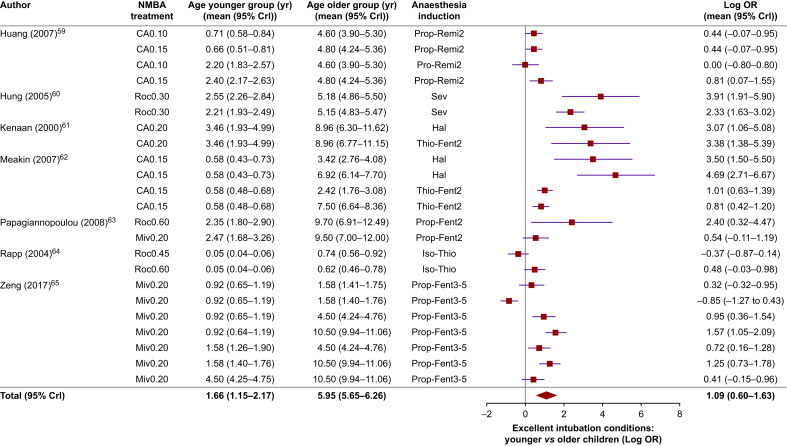
A pairwise meta-analysis evaluates the likelihood of achieving excellent intubation conditions in younger *vs* older paediatric patients, all receiving the same anaesthetic induction and neuromuscular blocking agent (NMBA) treatment. The drugs used for anaesthesia induction include: ‘Prop’ (propofol), ‘Thio’ (thiopental), ‘Hal’ (halothane), ‘Iso’ (isoflurane), ‘Sev’ (sevoflurane), ‘Fent’ (fentanyl), and ‘Remi’ (remifentanil). ‘Fent’ and ‘Remi’ are accompanied by their respective doses in micrograms per kilogram (μg kg^−1^). The results are reported as the log odds ratio (Log OR) with a 95% credible interval (mean [95% CrI]), indicating the difference in the proportion of excellent intubation conditions between the two age groups. A Log OR less than, equal to, or greater than 0 suggests that the treatment effects in the younger age group are smaller, the same, or larger, respectively, than those in the older children. Log OR: 1.09 (0.60–1.63) corresponds to OR: 2.97 (1.82–5.10).^59^, ^60^, ^61^, ^62^, ^63^, ^64^, ^65^

With similar NMBA treatment and anaesthesia induction, younger children (1.66 [1.15–2.17] yr) had better intubation conditions with NMBAs than did older children (5.95 [5.65–6.26] yr) for both excellent (OR: 2.97 [1.82–5.10]) (Fig. 5) and acceptable intubation conditions (OR: 2.29 [1.14–4.39]).^59^, ^60^, ^61^, ^62^, ^63^, ^64^, ^65^ The use of an NMBA significantly increased the likelihood of achieving excellent (OR: 5.05 [2.43–10.48]) and acceptable intubation conditions (OR: 6.23 [3.35–11.58]), compared with an induction without NMBAs (Supplementary material 11). Values are mean (95% CrI).

**Fig 6:**
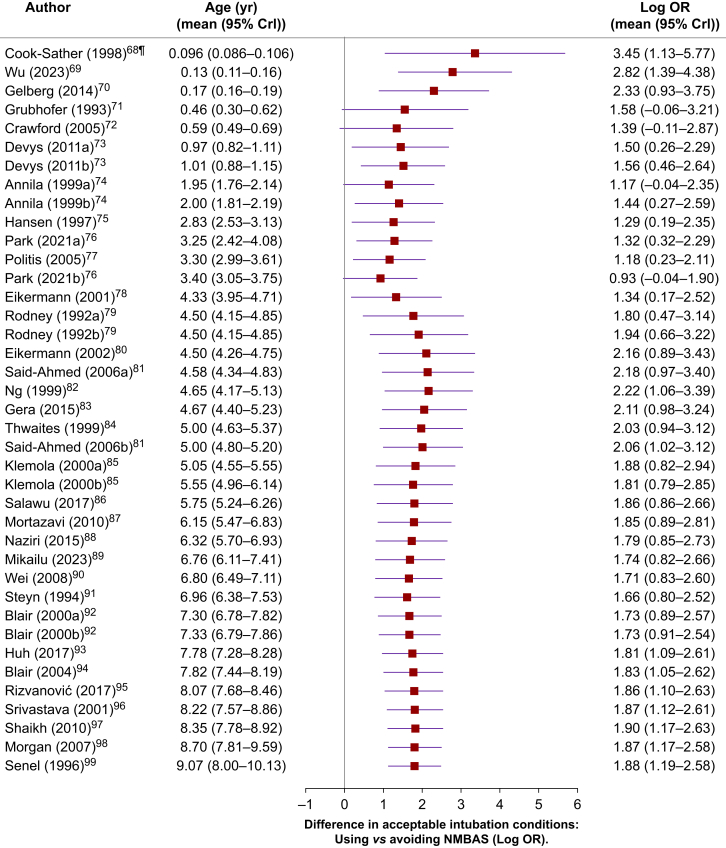
Cumulative meta-analysis of studies evaluating differences in acceptable intubation conditions between induction sequences that include neuromuscular blocking agents (NMBAs) and those that do not, represented as log odds ratios (Log ORs) with a 95% credible interval (mean [95% CrI]). Studies are added sequentially based on increasing mean participant age. Except for Cook-Sather and colleagues^68^ all included studies are RCTs. The analysis represents both the trend and magnitude of changes in effect size with increasing age. Log ORs less than, equal to, or greater than zero indicate that the proportion of acceptable intubation conditions achieved with NMBAs is inferior to, equivalent to, or superior to the proportion obtained without NMBAs, respectively.^68^, ^69^, ^70^, ^71^, ^72^, ^73^, ^74^, ^75^, ^76^, ^77^, ^78^, ^79^, ^80^, ^81^, ^82^, ^83^, ^84^, ^85^, ^86^, ^87^, ^88^, ^89^, ^90^, ^91^, ^92^, ^93^, ^94^, ^95^, ^96^, ^97^, ^98^, ^99^

Figure 6 presents a cumulative meta-analysis^66^^,^^67^ exploring the differences in acceptable intubation conditions between induction sequences that included NMBAs and those that did not. Studies are added sequentially based on increasing mean participant age.^68^, ^69^, ^70^, ^71^, ^72^, ^73^, ^74^, ^75^, ^76^, ^77^, ^78^, ^79^, ^80^, ^81^, ^82^, ^83^, ^84^, ^85^, ^86^, ^87^, ^88^, ^89^, ^90^, ^91^, ^92^, ^93^, ^94^, ^95^, ^96^, ^97^, ^98^, ^99^ The analysis represents both the trend and magnitude of changes in effect size across developmental stages.^40^

The findings indicate that intubation without NMBA was most difficult in newborns and infants and became increasingly easier up to approximately 4 yr of age. Beyond this age, the difference in acceptable intubation conditions between NMBA and NMBA-free groups became larger, indicating a greater benefit of NMBA use in older children.

Pairwise meta-analyses evaluated HR and MAP in comparative trials between groups that received NMBAs and those that did not. Although anticholinergic use was consistent across groups, differences in anaesthesia induction protocols were identified between the NMBA and NMBA-free groups (Supplementary material 11).

Differences in haemodynamic parameters were assessed at three key time points: immediately before intubation and at 1 and 3 mins after laryngoscopy and intubation. The mean MAP differences between induction techniques involving NMBAs and those without were 12.30 (7.59–17.10) mm Hg before intubation, 17.07 (11.19–23.02) mm Hg at 1 min post-intubation, and 7.43 (1.82–16.88) mm Hg at 3 min post-intubation. Similarly, the mean HR differences were 13.19 (8.65–17.83) beats min^−1^ before intubation, 11.24 (6.99–15.98) beats min^−1^ at 1 min post-intubation, and 5.73 (4.03–7.80) beats min^−1^ at 3 min post-intubation (Supplementary material 11).

Trial sequential analysis^46^ confirmed that the amount of available information (represented by the number of participants included in the meta-analysis) was sufficient to validate or refute the effect of a specific intervention (Supplementary material 12).

Meta-regression analyses^47^^,^^48^ show that the disparity in intubation quality—measured by the Log OR of excellent or acceptable conditions—between induction sequences using NMBAs and those avoiding them increased with age, and suxamethonium use (Supplementary materials 11 and 13).

Although opioids enhanced intubation conditions in NMBA-free groups, they did not significantly affect conditions when moderate-to-high doses of NMBAs were used. Moreover, opioids typically stabilised or decreased HR and MAP. In contrast, anticholinergics and suxamethonium—whether administered alone^74^^,^^82^^,^^88^^,^^98^ or in combination^72^^,^^75^^,^^79^^,^^86^^,^^95^, ^96^, ^97^—were associated with tachycardia, hypertension, and arrhythmias,^74^^,^^75^ particularly within the first few minutes after administration (Supplementary materials 11 and 13).

### Sensitivity analyses and credibility of the results

Sensitivity analyses indicated that including CCTs or studies with a small number of ASA physical status 3 patients,^54^, ^55^, ^56^ with a high risk of bias (Supplementary material 6), lacking sample size calculations or that were published before the year 2000 (Supplementary material 3) did not affect the overall results.

CINeMA assessment^38^ showed moderate-to-low confidence in the recommendations drawn from this meta-analysis (Supplementary material 14).

## Discussion

We present the first large-scale systematic review comparing the efficacy and the relative merits of commonly used NMBA treatments to facilitate tracheal intubation in paediatric patients. We further explored the effects of characteristics such as timing of intubation, choice of induction agents, and patient age, and quantified their impact on the variability of outcomes.

Suxamethonium ≥1.50 mg kg^−1^ and rocuronium ≥0.90 mg kg^−1^ produced the most effective and rapid intubation conditions, though these effects did not consistently occur within 60 s post-administration. Other NMBA interventions showed, on average, lower efficacy and slower onset, with longer time to intubation potentially improving quality of airway management. Reduced NMBA dosing and NMBA-free induction protocols generally resulted in less favourable intubation conditions.

Non-depolarising NMBAs acted faster and enhanced excellent and acceptable intubation conditions more in younger than in older children. Intubation without NMBAs was most challenging in neonates and infants, improving up to approximately age 4 yr. Beyond this, the difference in intubation quality between groups that received NMBAs and those that did not increased with age, indicating that NMBAs may confer a greater benefit in older children.

Inhalation anaesthetics enhanced the proportion of excellent, but not acceptable, intubation conditions when used with NMBAs. Opioids decreased MAP and HR but did not improve intubation conditions with suxamethonium ≥1.00 mg kg^−1^ or rocuronium ≥0.90 mg kg^−1^.

The optimal NMBA dose needed to facilitate tracheal intubation is controversial, with advocates and opponents for both higher and lower doses of such an agent. Multiples of ED95 dose equivalents, such as vecuronium ≥0.20 mg kg^−1^ or rocuronium ≥0.90 mg kg^−1^, induce rapid and profound neuromuscular block comparable with that of suxamethonium ≥1.00 mg kg^−1^ but are associated with significantly longer recovery times.^56^^,^^57^ Conversely, although lower doses of these NMBAs allow for faster recovery, they make excellent intubation conditions less probable, intubation attempts more likely to fail, and the vocal cords more susceptible to damage.^9^, ^10^, ^11^^,^^78^^,^^80^^,^^93^

The timing of intubation after NMBA administration is essential for the smooth insertion of the tracheal tube.^100^^,^^101^ High doses of low-potency NMBAs, defined by a higher ED95, such as suxamethonium ≥1.00 mg kg^−1^ or rocuronium ≥0.90 mg kg^−1^, tend to produce faster, more consistent, and superior intubation conditions.^102^, ^103^, ^104^ In contrast, equi-effective doses of more potent agents such as mivacurium^65^^,^^105^ and cisatracurium^59^^,^^62^^,^^106^ are associated with slower and more variable times to peak effect,^105^^,^^106^ often resulting in less predictable intubation conditions unless guided by neuromuscular monitoring.^3^^,^^4^^,^^107^

Intubation without NMBAs can be especially difficult in neonates and infants, often requiring multiple attempts, sometimes up to 10.^9^, ^10^, ^11^, ^12^, ^13^, ^14^, ^15^, ^16^^,^^68^^,^^69^ High-quality studies in children from a few weeks to 11 yr old show that non-depolarising NMBAs act faster and improve intubation conditions more effectively in younger children than in older ones.^58^, ^59^, ^60^, ^61^, ^62^ The more rapid-onset and better intubation conditions seen in younger children are largely attributable to faster drug delivery^59^^,^^62^^,^^100^, ^101^, ^102^, ^103^, ^104^, ^105^^,^^108^, ^109^, ^110^, greater NMBA sensitivity,^111^, ^112^, ^113^, ^114^, ^115^ and more compliant upper airway structures^116^—factors that diminish with age.

The relative benefit of using NMBAs increases with age, as older children tend to have more vigorous responses to airway manipulation—such as coughing, arousal, and muscle tension^116^, ^117^, ^118^, ^119^, ^120^—increasing the difficulty of intubation in the absence of neuromuscular block.^68^, ^69^, ^70^, ^71^, ^72^, ^73^, ^74^, ^75^, ^76^, ^77^, ^78^, ^79^, ^80^, ^81^, ^82^, ^83^, ^84^, ^85^, ^86^, ^87^, ^88^, ^89^, ^90^, ^91^, ^92^, ^93^, ^94^, ^95^, ^96^, ^97^, ^98^, ^99^

The success of airway management relies on the interplay between neuromuscular block, the depth of anaesthesia, and the technical proficiency of the intubating professional.^9^, ^10^, ^11^^,^^68^^,^^69^^,^^78^^,^^80^^,^^85^ A deficiency in one of these factors can often be compensated by optimising the others.^121^ Favourable intubation conditions with slower-acting NMBA treatments may result from deeper CNS depression induced by higher anaesthetic doses, even before complete neuromuscular paralysis is achieved.^85^ Although avoiding NMBAs never improves the quality of intubation,^14^^,^^23^^,^^24^ with sufficient depth of anaesthesia, in the right experienced hands and in the appropriate patient group, such as children aged 1–4 yr, airway management without NMBAs could be a feasible option.^14^^,^^76^^,^^77^

NMBAs facilitate intubation by decreasing muscle tone, reducing the force (torque^122^) required for laryngoscopy, and limiting autonomic and haemodynamic activation during airway instrumentation.^123^^,^^124^ Our findings, however, indicate that suxamethonium, may paradoxically enhance cardiovascular responses. These effects may arise from the depolarising agent’s complex interaction with autonomous cholinergic receptors, potentially altering the balance between sympathetic and vagal tone. Furthermore, factors such as inadequate anaesthetic depth, fasciculations, anticholinergic co-administration, or all of the aforementioned likely contributed to the overall haemodynamic profile.^74^^,^^75^^,^^79^^,^^92^^,^^94^^,^^125^^,^^126^

The addition of opioids to the induction sequence facilitates airway insertion and decreases the haemodynamic responses to intubation Moreover, opioids, hypnotics, and inhalation anaesthetics act synergistically within the CNS.^70^^,^^85^^,^^88^^,^^127^, ^128^, ^129^, ^130^ Depending on their type, concentration, and duration of exposure, inhalation anaesthetics can potentiate NMBA effect.^61^^,^^62^^,^^115^^,^^131^ Although opioids decreased MAP and HR in our study, they provided no additional benefit beyond the rapid and profound neuromuscular block achieved with suxamethonium ≥1.00 mg kg^−1^ or rocuronium ≥0.90 mg kg^−1^ for airway management in children.^132^^,^^133^

Owing to the limitations imposed by circulation time and muscle perfusion,^134^, ^135^, ^136^ doses of suxamethonium up to 2.00 mg kg^−1^ or rocuronium ≥0.90 mg kg^−1^ may not produce optimal intubation conditions within 60 s.^102^^,^^137^ However, key aspects of a modified rapid sequence induction and intubation in paediatric patients include achieving deep anaesthesia, using gentle face mask ventilation to mitigate rapid oxygen desaturation during apnoea,^17^^,^^137^^,^^138^ and confirming profound neuromuscular block through nerve stimulation rather than relying solely on the standard 60-s rule.^3^^,^^4^^,^^139^^,^^140^

### Methodological limitations and generalisability concerns

Participants were enrolled over a 39-yr period, 28% of whom were recruited before 2000, mainly in studies with atracurium, vecuronium, or suxamethonium, to improve comparability and statistical strength. Cochrane's updated guidelines allow inclusion of older studies regardless of publication date, provided they meet current eligibility criteria.^141^ Although no association was found between publication year and outcomes, subtle changes of clinical practice remain a common limitation in meta-analyses, especially in continuously evolving fields.^142^

Restricting inclusion to studies that used direct laryngoscopy for tracheal intubation—a standard and widely accepted method in paediatric anaesthesia—helps reduce variability and more accurately attribute differences in outcomes to NMBA use and anaesthetic practices. Although videolaryngoscopy is increasingly changing intubation practice, paediatric studies comparing NMBAs using this technique remain limited; most investigations focus on comparisons with direct laryngoscopy or specific clinical scenarios.^10^^,^^11^^,^^143^^,^^144^

Differences in induction protocols between NMBA and non-NMBA groups in comparative trials may have confounded the assessment of haemodynamic outcomes. Despite similar anticholinergic use between groups, the elevated MAP and HR observed in the NMBA arms could indicate a lighter depth of anaesthesia rather than a direct effect of the NMBAs themselves.

Rating scales for intubation conditions are primarily designed for adults and lack validation in children, whose airway anatomy and physiological responses to intubation differ significantly.^18^^,^^20^^,^^116^, ^117^, ^118^ Despite limitations, these scales offer a useful framework for comparing NMBA effectiveness in paediatric airway management.

A key challenge in paediatric intubation research relates to limitations of current scoring systems, which mainly focus on the endpoint of merely inserting a tracheal tube between the vocal cords. This raises questions about what distinguishes ‘good’ from ‘excellent’ conditions, whether both are truly acceptable, and to what extent the intubator’s assessment of ‘acceptable’ adequately reflects patient-centred outcomes such as minimal haemodynamic response and absence of morbidity.^24^^,^^145^, ^146^, ^147^ Should future grading systems, therefore, include a post-intubation component to better capture these outcomes, and should time to intubation also be evaluated as a scalable factor?^17^^,^^148^ Moreover, future research on paediatric intubation should aim to develop reliable methods to assess and quantify clinician proficiency.^149^

The confidence in the recommendations drawn from this meta-analysis is rated as low to moderate, mainly owing to several concerns about the risk of bias, incomplete reporting, and clinical heterogeneity among the included studies. Although the assumptions of transitivity and consistency were met, and the study population primarily included healthy children aged 2–12 yr, the findings should still be interpreted with caution. This is owing to the inherent dependence of network meta-analysis on the similarity of included studies and the quality of their data.^38^^,^^45^

In conclusion, we conducted a systematic review and meta-analysis to synthesise and consolidate evidence on the comparative effectiveness of commonly used interventions for facilitating tracheal tube insertion.

Rocuronium ≥0.90 mg kg^−1^ offers intubation quality similar to that of suxamethonium ≥1.50 mg kg^−1^ with fewer side-effects, though not reliably within 60s. Other NMBA interventions demonstrated, on average, lower effectiveness and slower onset, underscoring the importance of neuromuscular monitoring. Intubation without NMBAs is most challenging in neonates and infants, tends to become easier up to approximately age 4 yr, and then becomes more difficult as children grow older, making the use of NMBAs increasingly beneficial with age.

The confidence in making recommendations informed by the current meta-analysis can be rated as low to moderate. Therefore, further investigation is warranted to strengthen the evidence and improve the reliability and validity of the recommendations. Furthermore, the increasing use of videolaryngoscopy in paediatric airway management represents an important area for future research on intubation with *neuromuscular blocking agents*.^150^

## Authors’ contributions

Study design: LEV, JJD, RJS, EML, LMS

Study conception: LEV

Data collection: LEV, JMB

Statistical analysis: LEV

Data interpretation: LEV, JJD, EML, LMS

Drafted the original manuscript: LEV

Critically revised the manuscript for important intellectual content: JJD, RJS, EML, JMB, LMS

Project oversight and leadership: LMS

Approved the final version for publication and agreed to be accountable for all aspects of the work, ensuring the accuracy and integrity of the content: all authors

## Funding

Department of Anaesthesiology, Erasmus MC, University Medical Centre Rotterdam, Rotterdam, The Netherlands.

## Declaration of interest

The Department of Anaesthesiology of the Erasmus Medical Centre in Rotterdam received funding for research activities by MSD/Merck.
