# Supplementary material for: Effect of neuromuscular blocking agents on tracheal intubation quality in paediatric patients: a systematic review using network meta-analysis and meta-regression
Source: Br J Anaesth. 2025 Sep 3;135(6):1787–802. doi: 10.1016/j.bja.2025.08.036 (PMC12799451; doi:10.1016/j.bja.2025.08.036)
Supplement: Multimedia Component 2 [file mmc2.docx]

**Supplementary material File 2: search strategy and search strings.**

1. **Medline ALL Ovid**

(Neuromuscular Blocking Agents / OR Rocuronium / OR Succinylcholine / OR Mivacurium / OR Atracurium / OR Vecuronium Bromide / OR Cisatracurium.nm. OR Sugammadex/ OR ((neuromuscul* ADJ3 block*) OR (muscle* ADJ3 relaxant*) OR Rocuron* OR Suxamethon* OR Mivacur* OR atracur* OR Vecuron* OR Cisatracur* OR Sugammad* OR bridion* OR nimbex* OR tracrium* OR mivacron* OR norcuron* OR succinylcholin*).ab,ti.) AND (Intubation/ OR Intubation, Intratracheal/ OR (intubation* OR (endotrachea* ADJ3 tube*) OR (airway* ADJ3 (insert* OR secur*))).ab,ti.) AND (exp Child/ OR exp Infant/ OR Adolescent/ OR Pediatrics/ OR (adolescen* or child* OR infan* OR pediatr* OR paediatr* OR neonat*).ab,ti.) NOT (*Critical Care / OR exp * Intensive Care Units / OR Infant, Premature / OR (intensive-care OR prematur*).ti.) AND (Exp Controlled clinical trial/ OR "Double-Blind Method"/ OR "Single-Blind Method"/ OR "Random Allocation"/ OR (random* OR factorial* OR crossover* OR cross over* OR placebo* OR ((doubl* OR singl*) ADJ blind*) OR assign* OR allocat* OR volunteer* OR trial OR groups).ab,ti,kf.) NOT (exp Animals/ NOT Humans/)

1. **embase.com**

('neuromuscular blocking agent'/de OR 'muscle relaxant agent'/de OR Rocuronium/de OR Suxamethonium/de OR Mivacurium/de OR 'atracurium besilate'/de OR Vecuronium/de OR Cisatracurium/de OR Sugammadex/de OR ((neuromuscul* NEAR/3 block*) OR (muscle* NEAR/3 relaxant*) OR Rocuron* OR Suxamethon* OR Mivacur* OR atracur* OR Vecuron* OR Cisatracur* OR Sugammad* OR bridion* OR nimbex* OR tracrium* OR mivacron* OR norcuron* OR succinylcholin*):ab,ti) AND (intubation/de OR 'respiratory tract intubation'/exp OR 'endotracheal tube'/exp OR (intubation* OR (endotrachea* NEAR/3 tube*) OR (airway* NEAR/3 (insert* OR secur*))):Ab,ti) AND (child/exp OR adolescent/exp OR pediatrics/de OR 'pediatric anesthesia'/de OR (adolescen* or child* OR infan* OR pediatr* OR paediatr* OR neonat*):ab,ti) NOT ('intensive care'/exp/mj OR 'intensive care unit'/exp/mj OR prematurity/mj OR (intensive-care OR prematur*):ti) AND ('Controlled clinical trial'/exp OR 'Crossover procedure'/de OR 'Double-blind procedure'/de OR 'Single-blind procedure'/de OR (random* OR factorial* OR crossover* OR (cross NEXT/1 over*) OR placebo* OR ((doubl* OR singl*) NEXT/1 blind*) OR assign* OR allocat* OR volunteer* OR trial OR groups):ab,ti,kw) NOT ((animal/exp OR animal*:de OR nonhuman/de) NOT ('human'/exp))

1. **Web of Science**

TS=((((neuromuscul* NEAR/2 block*) OR (muscle* NEAR/2 relaxant*) OR Rocuron* OR Suxamethon* OR Mivacur* OR atracur* OR Vecuron* OR Cisatracur* OR Sugammad* OR bridion* OR nimbex* OR tracrium* OR mivacron* OR norcuron* OR succinylcholin*)) AND ((intubation* OR (endotrachea* NEAR/2 tube*) OR (airway* NEAR/2 (insert* OR secur*)))) AND ((adolescen* or child* OR infan* OR pediatr* OR paediatr* OR neonat*)) AND ((random* OR factorial* OR crossover* OR (cross NEAR/1 over*) OR placebo* OR ((doubl* OR singl*) NEAR/1 blind*) OR assign* OR allocat* OR volunteer* OR trial OR groups))) NOT TI=((intensive-care OR prematur*))

1. **Scopus**

TITLE-ABS-KEY((((neuromuscul* W/2 block*) OR (muscle* W/2 relaxant*) OR Rocuron* OR Suxamethon* OR Mivacur* OR atracur* OR Vecuron* OR Cisatracur* OR Sugammad* OR bridion* OR nimbex* OR tracrium* OR mivacron* OR norcuron* OR succinylcholin*))

AND ((intubation* OR (endotrachea* W/2 tube*) OR (airway* W/2 (insert* OR secur*))))

AND ((adolescen* OR child* OR infan* OR pediatr* OR paediatr* OR neonat*))

AND ((random* OR trial))) AND NOT TITLE((intensive-care OR prematur*))

1. **Cochrane CENTRAL**

(((neuromuscul* NEAR/3 block*) OR (muscle* NEAR/3 relaxant*) OR Rocuron* OR Suxamethon* OR Mivacur* OR atracur* OR Vecuron* OR Cisatracur* OR Sugammad* OR bridion* OR nimbex* OR tracrium* OR mivacron* OR norcuron* OR succinylcholin*):ab,ti) AND ((intubation* OR (endotrachea* NEAR/3 tube*) OR (airway* NEAR/3 (insert* OR secur*))):Ab,ti) AND ((adolescen* or child* OR infan* OR pediatr* OR paediatr* OR neonat*):ab,ti) NOT ((intensive-care OR prematur*):ti)

1. **Scielo**

("intubación orotraqueal" OR "condiciones de intubación" OR puntuación OR "intubation conditions" OR "tracheal intubation") AND (atrac* OR mivacur* OR rocur* OR cisatrac* OR vecuron* OR succinilcolina OR succinylcholine OR "relajantes musculares" OR "bloqueadores neuromusculares" OR "muscle relaxants" OR "neuromuscular block*") AND (niño* OR pediatr* OR neonat* OR infant* OR child*)

1. **CKNI**

(neuromuscular-block* OR muscle-relaxant*) AND (intubation* OR endotracheal-tube*) AND (adolescen* or child* OR infan* OR pediatr*) AND (random* OR trial)
