# Supplementary material for: Effect of neuromuscular blocking agents on tracheal intubation quality in paediatric patients: a systematic review using network meta-analysis and meta-regression
Source: Br J Anaesth. 2025 Sep 3;135(6):1787–802. doi: 10.1016/j.bja.2025.08.036 (PMC12799451; doi:10.1016/j.bja.2025.08.036)
Supplement: Multimedia Component 3 [file mmc3.docx]

**Supplementary material File 3a: Procedural, patient, and intubation characteristics of included trials.**

| **Auteur** | **NMBA**  **Treatment** | **Node** | **Premedication** | **Induction sequence** | **RCT** | **age (sd)**  **(yr)** | **weight (sd)**  **(kg)** | **. t_int (sd)(** | **total**  **(n)** | **EiC**  **(n)** | **AIC**  **(n)** | **Fail**  **(n)** |
| --- | --- | --- | --- | --- | --- | --- | --- | --- | --- | --- | --- | --- |
|  |  |  |  |  |  |  |  |  |  |  |  |  |
| 1. An (2003)& [S1] | Atr0.50 | Atr0.50 | glp 4 µg∙kg^-1^ im; and  mdz 0.05 mg∙kg^-1^ im | thiopentone 5 mg∙kg^-1^; and sevoflurane 2% IC in N_2_O 60%. | 1 | 6.60 (2.30) | 32.70 (10.50) | 176 (23) | 20 | 14 | 19 | 0 |
|  | Vec0.10 | Vec0.10 |  |  |  | 5.10 (3.10) | 26.10 (7.10) | 150 (19) | 20 | 16 | 20 | 0 |
|  | Roc0.60 | Roc0.60 |  |  |  | 5.50 (2.40) | 28.30 (9.60) | 100 (20) | 20 | 18 | 20 | 0 |
| 2. Annila (1999)¶ [S2] | Sux1.50 | Sux1.50 | no premedication | thiopentone 5 mg∙kg^-1^ | 1 | 1.90 (0.80) | 11.80 (1.40) | NA | 30 | 22 | 29 | 0 |
|  | NO | PL-Op |  | propofol 3 mg∙kg^-1^; and alfentanil 10 µg∙kg^-1^ |  | 2.10 (0.70) | 12.30 (1.80) | NA | 30 | 1 | 14 | 6 |
|  | NO | PL |  | halothane 5% IC |  | 2.00 (0.70) | 12.20 (0.70) | NA | 30 | 22 | 30 | 0 |
| 3. Apilioğulları (2006)& [S3] | Miv0.20 | Miv0.20 | atr 15 µg∙kg^-1^ po; and  mdz 0.50 mg∙kg^-1^ po | sevoflurane 5% IC in N2O 50% | 1 | 5.70 (2.80) | 19.57 (7.27) | 226 (119) | 60 | 60 | 60 | 0 |
|  | Roc0.60 | Roc0.60 |  |  |  | 6.52 (2.99) | 21.57 (6.29) | 182 (89) | 60 | 60 | 60 | 0 |
| 4. Bala (2024)†¶& [S4] | CA0.15 | CA0.15 | glp; 5 µg∙kg^-1^  fentanyl 2 µg∙kg^-1^ IV. | sevoflurane 1 MAC  or propofol 2 mg∙kg^-1^. | 1 | 4.55 (2.02) | NA | 148.2 (35.4) | 40 | 38 | 40 | 0 |
|  | Atr0.50 | Atr0.50 |  |  |  | 4.35 (2.01) | NA | 127.2 (37.2) | 40 | 40 | 40 | 0 |
| 5. Balakrishnan (1999)& [S5] | Miv0.20 | Miv0.20 | glp 10 µg∙kg^-1^ im; and  pentazocine 0.50 mg∙kg^-1^ im | thiopentone 5 mg∙kg^-1^ | 1 | 8.00 (2.00) | NA | 120 | 25 | 19 | 25 | 0 |
|  | Atr0.50 | Atr0.50 |  |  |  | 8.00 (2.00) | NA | 120 | 25 | 13 | 22 | 3 |
| 6. Bansal (2023)†& [S6] | Atr0.50 | Atr0.50 | glp 4 µg∙kg^-1^ iv; and  fentanyl 2µg∙kg^-1^ iv | sevoflurane 6% IC in N2O 50% | 1 | 6.51 (3.12) | 20.71 (8.72) | 60 | 35 | 10 | 15 | 0 |
|  | Roc0.60 | Roc0.60 |  |  |  | 6.34 (3.14) | 19.94 (8.02) | 60 | 35 | 30 | 33 | 0 |
| 7. Bartolek (2010)†& [S7] | Vec0.10 | Vec0.10 | mdz 0.25 mg∙kg^-1^ po | alfentanil 20 µg∙kg^-1^; and propofol 2 mg∙kg^-1^; | 1 | 7.20 (0.58) | 22.40 (1.79) | 132 (9) | 35 | 26 | 33 | 0 |
|  | Roc0.60 | Roc0.60 |  |  |  | 7.26 (0.78) | 22.80 (1.97) | 84 (10) | 35 | 29 | 33 | 0 |
|  | Roc0.45 | Roc0.45 |  |  |  | 7.23 (0.55) | 22.60 (2.53) | 186 (20) | 35 | 19 | 33 | 0 |
| 8. Barve (2002)¶& [S8] | Roc0.60 | Roc0.60 | mdz 0.05 mg∙kg^-1^ iv; and  tramadol 2 mg∙kg^-1^ iv. | thiopentone 5-7 mg∙kg^-1^ | 1 | 3.25 (1.59) | 11.33 (3.24) | 71 (14) | 20 | 6 | 15 | 0 |
|  | Sux1.00 | Sux1.00 |  |  |  | 3.00 (1.45) | 11.10 (2.57) | 60 | 20 | 10 | 18 | 0 |
| 9. Bhattacharya (2008)& [S9] | Vec0.10 | Vec0.10 | mdz 0.20-0.40 mg∙kg^-1^ po | thiopentone 5 mg∙kg^-1^, glp 10-20 mg·kg^-1^, tramadol 2-3 mg·kg^-1^ | 1 | 6.20 (3,51) | 17.84 (7,72) | 120 | 25 | 4 | 7 | 0 |
|  | Roc0.60 | Roc0.60 |  |  |  | 5.52 (2,42) | 16.22 (5,12) | 120 | 25 | 15 | 25 | 0 |
|  | Roc0.90 | Roc0.90 |  |  |  | 5.61 (2,14) | 16.84 (4,01) | 120 | 25 | 25 | 25 | 0 |
| 10. Blair (2000)† [S10] | Sux1.00 | Sux1.00 | no premedication | propofol 3 mg∙kg^-1^; and N_2_O 60%. | 1 | 7.52 (2.40) | 26.20 (10.25) | 60 | 40 | 28 | 39 | NA |
|  | NO | PL-Op |  | alfentanil 10 µg∙kg^-1^; and propofol 3 mg∙kg^-1^ |  | 7.08 (2.31) | 25.90 (15.38) | 60 | 40 | 3 | 21 | NA |
|  | NO | PL |  | sevoflurane 8% IC in N_2_O 60% |  | 7.13 (2.48) | 26.00 (9.50) | 180 | 40 | 18 | 35 | NA |
| 11. Blair (2004)† [S11] | Miv0.20 | Miv0.20 | no premedication | propofol 3 mg∙kg^-1^; atr 10 µg∙kg^-1^ iv; and N_2_O 60% | 1 | 8.06 (2.10) | 28.20 (7.50) | 95 | 28 | 16 | 27 | 0 |
|  | NO | PL-Op |  | atr 10 µg∙kg-1 iv; remifentanil 1 µg∙kg^-1^; propofol 3 mg∙kg^-1^; and N_2_O 60% |  | 7.80 (1.79) | 29.80 (10.50) | 60 | 28 | 8 | 14 | 6 |
|  | NO | PL-Op |  | atr 10 µg∙kg-1 iv; remifentanil 2 µg∙kg^-1^; propofol 3 mg∙kg^-1^; and N_2_O 60% |  | 7.25 (1.79) | 24.50 (6.25) | 60 | 26 | 9 | 18 | 1 |
|  | NO | PL-Op |  | atr 10 µg∙kg-1 iv; remifentanil 3 µg∙kg^-1^; propofol 3 mg∙kg^-1^; and N_2_O 60% |  | 8.11 (2.23) | 30.00 (2.50) | 60 | 27 | 13 | 22 | 4 |
| 12a. Bock (2007)†& [S12a] | Roc0.45 | Roc0.45 | mdz 0.45 mg∙kg^-1^ po | propofol 2.5-3.5 mg∙kg^-1^; and N_2_O 60%. | 1 | 4.10 (2.00) | 18.00 (8.40) | 123 (22) | 21 | 18 | 21 | 0 |
|  | Roc0.60 | Roc0.60 |  |  |  | 3.90 (1.70) | 18.00 (9.00) | 85 (21) | 21 | 19 | 21 | 0 |
| 12b. Bock (2007)†& [S12b] | Roc0.45 | Roc0.45 |  |  |  | 4.30 (1.70) | 18.00 (8.50) | 93 (12) | 20 | 16 | 20 | 0 |
|  | Roc0.60 | Roc0.60 |  |  |  | 3.90 (1.30) | 18.00 (8.50) | 55 (6) | 20 | 18 | 20 | 0 |
| 13. Bucx (1994) [S13] | Sux1.50 | Sux1.50 | atr 30 µg∙kg^-1^ po | propofol 2.5 mg∙kg^-1^ + lido 1%; fentanyl 2 µg∙kg^-1^; and N_2_O 60% | 1 | 7.80 (4.40) | 30.30 (17.00) | 86 (3) | 26 | 25 | 26 | 0 |
|  | Vec0.10 | Vec0.10 |  |  |  | 7.40 (4.00) | 27.50 (12.30) | 160 (3) | 28 | 22 | 28 | 0 |
| 14. Castillo-Peralta (2004)& [S14] | Roc0.30 | Roc0.30 | mdz 0.10 mg∙kg^-1^ iv | atr 10 µg∙kg^-1^; propofol 2-3 mg∙kg^-1^; and 1-3 fentanyl µg∙kg^-1^ | 0 | 1.04 (0.48) | NA | 60 | 10 | 1 | 9 | 0 |
|  | Roc0.60 | Roc0.60 |  |  |  | 1.04 (0.48) | NA | 60 | 10 | 8 | 10 | 0 |
|  | Roc0.90 | Roc0.90 |  |  |  | 1.04 (0.48) | NA | 60 | 10 | 10 | 10 | 0 |
| 15. Chee (1998)& [S15] | Miv0.20 | Miv0.20 | no premedication | either intravenously using thiopentone 5 mg∙kg^-1^;  or by inhalation using halothane 4% IC. | 1 | 5.40 (2.60) | 20.20 (9.00) | 108 (24) | 12 | 11 | 11 | 0 |
|  | Miv0.25 | Miv0.25 |  |  |  | 6.25 (3.70) | 24.20 (12.50) | 96 (30) | 8 | 8 | 8 | 0 |
| 16. Cheng (2002)¶ [S16] | Vec0.10 | Vec0.10 | mdz 0.50 mg∙kg^-1^ po | thiopentone 5 mg∙kg^-1^; halothane 1 MAC ET; and N_2_O 66%. | 1 | 5.10 (1.50) | NA | 159.5 (29) | 20 | 13 | 16 | 0 |
|  | Roc0.60 | Roc0.60 |  |  |  | 4.90 (1.40) | NA | 71.3 (19) | 20 | 14 | 17 | 0 |
|  | Roc0.90 | Roc0.90 |  |  |  | 5.20 (1.70) | 30.30 (17.00) | 55.5 (14) | 20 | 17 | 20 | 0 |
| 17. Cheng (2002)†¶& [S17] | Sux1.50 | Sux1.50 | no premedication | alfentanil 10 µg∙kg^-1^; and thiopentone 5 mg∙kg^-1^ | 1 | 4.60 (2.80) | 18.60 (8.60) | 30 | 40 | 37 | 40 | 0 |
|  | Roc0.90 | Roc0.90 |  |  |  | 4.90 (2.70) | 20.30 (9.50) | 30 | 40 | 38 | 39 | 0 |
|  | Roc0.60 | Roc0.60 |  |  |  | 5.50 (2.60) | 21.10 (7.80) | 30 | 40 | 29 | 36 | 0 |
| 18a. Cook (1995)†& [S18a] | Miv0.15 | Miv0.15 | no premedication | atr 100 µg∙iv; and halothane 4% IC in N_2_O 70%. | 1 | 0.48 (0.20) | 7.40 (2.00) | 90 (48) | 10 | 6 | 9 | 0 |
|  | Sux1.60 | Sux1.50 |  |  |  | 0.42 (0.20) | 7.00 (1.00) | 66 (48) | 10 | 10 | 10 | 0 |
| 18b. Cook (1995)†& [S18b] | Miv0.20 | Miv0.20 |  |  |  | 6.5 (3.80) | 26.20 (18.00) | 84 (36) | 12 | 10 | 12 | 0 |
|  | Sux0.90 | Sux1.00 |  |  |  | 5.7 (3.10) | 21.00 (8.00) | 54 (12) | 12 | 12 | 12 | 0 |
| 18c. Cook (1995)†& [S18c] | Miv0.30 | Miv0.30 |  |  |  | 5.10 (2.00) | 18.60 (8.10) | 96 (4) | 12 | 12 | 12 | 0 |
|  | Sux1.50 | Sux1.50 |  |  |  | 4.90 (1.80) | 19.00 (5.70) | 54 (6) | 12 | 12 | 12 | 0 |
| 19. Cook-Sather (1998)¶ [S19] | NO | PL | atr 20 µg∙kg^-1^ po | no | 0 | 0.094 (0.042) | 3.80 (0.80) | 63 [29-252] | 22 | NA | 14 | 8 |
|  | Sux2.00 | Sux2.00 |  | thiopentone 5 - 7 mg∙kg^-1^ |  | 0.098 (0.046) | 4.00 (0.80) | 30 [10-93] | 28 | NA | 26 | 2 |
|  | NMBA‡ | NMBA‡ |  | thiopentone 5 - 7 mg∙kg^-1^ |  | 0.096 (0.042) | 3.80 (0.60) | 36 [10-206] | 26 | NA | 21 | 5 |
| 20. Crawford (2005)† [S20] | Sux2.00 | Sux2.00 | glp 10µg∙kg^-1^ iv | propofol 4 mg∙kg^-1^; lido 0.20 mg∙kg^-1^; and N_2_O 60%. | 1 | 0.59 (0.26) | 8.00 (2.20) | 105 (3) | 12 | 11 | 12 | 0 |
|  | Placebo | PL-Op |  | propofol 4 mg∙kg^-1^; lido 0.20 mg∙kg^-1^; remifentanil 3 µg∙kg^-1^; N_2_O 70%. |  | 0.59 (0.26) | 8.00 (2.20) | 103 (3) | 12 | 12 | 12 | 0 |
| 21. Deepak (2024)†¶& [S21] | CA0.15 | CA0.15 | mdz 0.10; mg∙kg^-1^; glp 1 mg∙kg^-1^ iv; and fentanyl 2 µg∙kg^-1^ iv | ketamine: 2 mg∙kg-1 | 1 | 6.79 (3.29) | 20.28 (8.61) | 81.6 (45) | 25 | 21 | 25 | 0 |
|  | Roc0.60 | Roc0.60 |  |  |  | 7.78 (3.29) | 21.20 (6.81) | 45.6 (43) | 25 | 25 | 25 | 0 |
| 22. Desai (2019)& [S22] | Roc0.90 | Roc0.90 | atr 15 µg∙kg^-1^ iv | fentanyl 1.0 µg∙kg-1; and thiopentone 5 mg∙kg^-1^ | 1 | 4.15 (1.28) | 12.20 (2.28) | 60 | 25 | 20 | 25 | 0 |
|  | Sux1.50 | Sux1.50 |  |  |  | 4.00 (1.29) | 12.15 (2.60) | 60 | 25 | 25 | 25 | 0 |
| 23. Devys (2011)†¶& [S23] | NO | PL | no premedication | sevoflurane 8% IC | 1 | 1.03 (0.50) | 8.60 (2.40) | 90 | 27 | 4 | 17 | 7 |
|  | NO | PL-Op |  | sevoflurane 8% IC; and alfentanil 20 µg∙kg^-1^. |  | 0.94 (0.55) | 8.80 (2.60) | 90 | 23 | 7 | 16 | 5 |
|  | Roc0.30 | Roc0.30 |  | sevoflurane 8% IC |  | 0.99 (0.50) | 8.80 (2.50) | 90 | 25 | 16 | 23 | 2 |
| 24. Eikermann (2001)†¶& [S24] | NO | PL | mdz 0.50 mg∙kg^-1^ po | sevoflurane 2% ET in N_2_O 60% | 1 | 4.33 (1.50) | 19.00 (6.00) | 120 | 10 | 0 | 1 | 1 |
|  | Roc0.15 | Roc0.15 |  |  |  | 4.33 (1.50) | 19.00 (6.00) | 120 | 10 | 6 | 7 | 1 |
|  | Roc0.22 | Roc0.20 |  |  |  | 4.33 (1.50) | 19.00 (6.00) | 120 | 10 | 7 | 9 | 0 |
|  | Roc0.33 | Roc0.30 |  |  |  | 4.33 (1.50) | 19.00 (6.00) | 120 | 10 | 8 | 10 | 0 |
|  | Roc0.50 | Roc0.45 |  |  |  | 4.50 (1.25) | 19.00 (6.00) | 120 | 10 | 8 | 10 | 0 |
|  | Roc1.00 | Roc0.90 |  |  |  | 4.50 (1.25) | 19.00 (6.00) | 120 | 10 | 9 | 10 | 0 |
| 25. Eikermann (2002)†¶& [S25] | NO | PL | mdz 0.50 mg∙kg^-1^ po | sevoflurane 8% IC in N_2_O 60% | 1 | 4.50 (1.25) | 19.00 (6.00) | 120 | 20 | 6 | 7 | 7 |
|  | Roc0.15 | Roc0.15 |  |  |  | 4.50 (1.25) | 19.00 (6.00) | 120 | 20 | 15 | 17 | 5 |
|  | Roc0.22 | Roc0.20 |  |  |  | 4.50 (1.25) | 19.00 (6.00) | 120 | 20 | 17 | 19 | 0 |
|  | Roc0.30 | Roc0.30 |  |  |  | 4.50 (1.25) | 19.00 (6.00) | 120 | 20 | 18 | 20 | 1 |
|  | Roc0.60 | Roc0.60 |  |  |  | 4.50 (1.25) | 19.00 (6.00) | 120 | 20 | 19 | 20 | 0 |
| 26. Fang (2015)¶& [S26] | Miv0.20 | Miv0.20 | phencyclidine 0.01 mg | Midazolam 0.010 mg∙kg^-1^; sufentanil 1 µg∙kg^-1^; propofol 1.5 mg∙kg^-1^ | 1 | 7.30 (2.10) | 22.00 (10.00) | 180 | 34 | 25 | 34 | 0 |
|  | CA0.10 | CA0.10 |  |  |  | 7.20 (2.00) | 24.00 (11.00) | 180 | 34 | 26 | 34 | 0 |
| 27. Fletcher (2004)¶ [S27] | Roc0.40 | Roc0.45 | no premedication | propofol 3 mg∙kg-1; fentanyl 2 µg∙kg-1 in N_2_O 70% | 1 | 3.83 (1.42) | 16.00 (4.00) | 60 | 11 | 7 | 9 | 0 |
|  | Miv0.133 | Miv0.15 |  |  |  | 3.50 (1.00) | 17.00 (5.00) | 60 | 12 | 1 | 7 | 2 |
| 28. Frediani (1993)& [S28] | Vec0.04 | Vec0.05 | hydroxyzine 1 mg∙kg^-1^ im  and atr 15 µg∙kg^-1^ | halothane 3% followed by isoflurane 3% IC in N_2_O 50% | 0 | 4.85 (1.58) | 19.90 (5.60) | 159 (17) | 57 | 53 | 55 | 2 |
|  | Atr0.20 | Atr0.25 |  |  |  | 4.55 (1.40) | 19.80 (6.70) | 165 (21) | 27 | 23 | 26 | 1 |
| 29. Friesdorf (1986)& [S29] | Vec0.08 | Vec0.05 | chlorprothixene 2 mg∙kg^-1^ | ketamine 2 mg∙kg^-1^ | 1 | 3.00 (1.23) | 14.00 (2.98) | 90 | 9 | 0 | 7 | 2 |
|  | Vec0.10 | Vec0.10 |  |  |  | 2.83 (1.23) | 17.00 (3.00) | 90 | 15 | 2 | 13 | 0 |
| 30. Fuchs-Buder (1996)¶& [S30] | Roc0.60 | Roc0.60 | mdz 0.20 mg∙kg^-1^ po | alfentanil 10 µg∙kg^-1^; and thiopentone 5 mg∙kg^-1^ | 1 | 5.00 (1.00) | 20.70 (4.80) | 60 | 35 | 29 | 35 | 0 |
|  | Roc0.90 | Roc0.90 |  |  |  | 5.00 (1.00) | 20.90 (4.50) | 60 | 35 | 33 | 35 | 0 |
| 31. Gelberg (2014)¶& [S31] | NO | PL-Op | mdz 0.30 mg∙kg^-1^ ir in 28/34 | remifentanil 2.3 µg∙kg^-1^; lido 1 mg∙kg^-1^; and propofol 3 mg∙kg^-1^ | 1 | 0.18 (0.06) | 5.3 (1.23) | 60 | 34 | 27 | 31 | 4 |
|  | Roc0.30 | Roc0.30 | mdz 0.30 mg∙kg^-1^ ir in 30/36 |  |  | 0.16 (0.07) | 5.3 (1.25) | 60 | 36 | 31 | 35 | 0 |
| 32. Gera (2015)†¶ [S32] | NO | PL-Op | mdz 0.50 mg∙kg^-1^ po | sevoflurane 4% IC; fentanyl 1 µg∙kg^-1^; and propofol 1.5 mg∙kg^-1^. | 1 | 4.72 (2.15) | 14.52 (4.25) | 180 | 25 | 19 | 24 | 0 |
|  | Roc0.60 | Roc0.60 |  | sevoflurane 4% IC; fentanyl 1 µg∙kg^-1^. |  | 4.62 (1.88) | 15.58 (3.87) | 180 | 25 | 19 | 24 | 0 |
| 33. Gnani (2017)† [S33] | Sux1.50 | Sux1.50 | mdz 0.05 µg∙kg^-1^ po | glp 0.01 mg∙kg^-1^; sevoflurane; propofol 1 mg∙kg^-1^; and fentanyl 2 µg∙kg^-1^. | 1 | 6.12 (1.98) | 14.57 (6.57) | 60 | 60 | 58 | 60 | 0 |
|  | Roc0.90 | Roc0.90 |  |  |  | 6.27 (2.94) | 16.00 (6.06) | 60 | 60 | 50 | 60 | 0 |
|  | Roc1.20 | Roc1.20 |  |  |  | 6.67 (2.65) | 16.77 (5.54) | 60 | 60 | 58 | 60 | 0 |
| 34. Gonzalez (2008) [S34] | Atr0.50 | Atr0.50 | mdz 0.10 mg∙kg^-1^; and atr 10 µg∙kg^-1^ iv | thiopentone 5 mg∙kg^-1^; fentanyl 2.5 µg∙kg^-1^; and N_2_O 50% | 1 | 8.50 (2.50) | 27.90 (5.90) | 180 | 50 | 47 | 50 | 0 |
|  | Vec0.10 | Vec0.10 |  |  |  | 8.00 (4.00) | 27.00 (8.10) | 180 | 50 | 49 | 50 | 0 |
| 35. Green (1998)& [S35] | Miv0.20 | Miv0.20 |  | standardized inhalational anaesthesia induction;  maintenance using isoflurane 0.5-1.0% ET. | 1 | 7.00 (5.00) | NA | 120 | 21 | 20 | 20 | 1 |
|  | Sux2.00 | Sux2.00 | glp 10 µg∙kg^-1^ iv |  |  | 7.00 (5.00) | NA | 60 | 16 | 14 | 15 | 0 |
| 36. Grubhofer (1993)¶& [S36] | NO | PL | mdz 1.00 mg∙kg^-1^ ir. | halothane 2% IC in N_2_O 67%; and propofol 3 mg∙kg^-1^ ; atr 20 µg∙kg^-1^ if hr<90 | 1 | 0.48 (0.48) | 5.90 (2.70) | 15 | 14 | 11 | 13 | 1 |
|  | Atr0.40 | Atr0.50 |  | halothane 2% IC in N_2_O 67%; atr 20 µg∙kg^-1^ if hr<90 |  | 0.44 (0.40) | 6.50 (3.00) | 120 | 14 | 10 | 12 | 2 |
| 37a. Grundman (1991)& [S37a] | Atr0.30 | Atr0.25 | atropine 10 µg∙kg^-1^ iv | halothane 1.5% IC in N_2_O 67% | 1 | 0.23 (0.15) | 5.70 (2.00) | 93 (32) | 10 | NA | 10 | 0 |
|  | Vec0.05 | Vec0.05 |  |  |  | 0.68 (0.60) | 7.10 (1.90) | 136 (46) | 10 | NA | 10 | 0 |
| 37b. Grundman (1991)& [S37b] | Atr0.50 | Atr0.50 | fentanyl 2 µg∙kg^-1^ im; DHBP 0.10 µg∙kg^-1^ im; and atropine 10 µg∙kg^-1^ iv |  |  | 3.43 (1.63) | 16.60 (4.90) | 117 (25) | 10 | NA | 10 | 0 |
|  | Vec0.10 | Vec0.10 |  |  |  | 3.28 (1.68) | 15.10 (2.90) | 123 (27) | 10 | NA | 10 | 0 |
| 38. Hansen (1997) [S38] | NO | PL | mdz 0.50 mg∙kg^-1^ ir | halothane 2.0% ET; and N_2_O 67 %. | 1 | 2.83 (1.50) | 15.00 (4.00) | 45 | 50 | NA | 48 | 1 |
|  | Sux1.50 | Sux1.50 |  | halothane 1.0% ET; and N_2_O 67 %. |  | 2.83 (1.58) | 14.00 (5.00) | 45 | 50 | NA | 48 | 2 |
| 39a. Huang (2007)& [S39a] | CA0.10 | CA0.10 | atropine 15 µg∙kg^-1^ im | remifentanil 2 µg∙kg^-1^; and propofol 1 mg∙kg^-1^ | 1 | 0.71 (0.27) | 7.20 (2.40) | 119 (15) | 20 | 14 | 20 | 0 |
|  | CA0.15 | CA0.15 |  |  |  | 0.66 (0.32) | 7.50 (3.20) | 103 (15) | 20 | 18 | 20 | 0 |
| 39b. Huang (2007)& [S39b] | CA0.10 | CA0.10 |  |  |  | 2.20 (0.80) | 14.50 (4.20) | 247 (18) | 20 | 12 | 20 | 0 |
|  | CA0.15 | CA0.15 |  |  |  | 2.40 (0.50) | 15.80 (4.70) | 179 (23) | 20 | 18 | 20 | 0 |
| 39c. Huang (2007)& [S39c] | CA0.10 | CA0.10 |  |  |  | 4.60 (1.50) | 20.50 (5.60) | 250 (19) | 20 | 12 | 20 | 0 |
|  | CA0.15 | CA0.15 |  |  |  | 4.80 (1.20) | 22.30 (6.40) | 186 (16) | 20 | 16 | 20 | 0 |
| 40. Huh (2017)† [S40] | NO | PL-Op | no premedication | propofol 2.5 mg∙kg^-1^; fentanyl 2 µg∙kg^-1^; and sevoflurane 5% IC. | 1 | 7.39 (2.18) | 29.90 (9.60) | 120 | 25 | 5 | 18 | 0 |
|  | Roc0.15 | Roc0.15 |  |  |  | 8.31 (2.12) | 32.80 (8.00) | 120 | 25 | 11 | 24 | 0 |
|  | Roc0.30 | Roc0.30 |  |  |  | 7.63 (2.35) | 26.90 (8.10) | 120 | 25 | 20 | 25 | 0 |
| 41. Kapdi (2020)& [S41] | Roc0.60 | Roc0.60 | glp 4 µg∙kg^-1^ iv,  mdz 0.02 mg∙kg^-1^ iv, and fentanyl 1 µg∙kg^-1^ iv | thiopentone 5 mg∙kg^-1^ | 1 | 6.20 (1.20) | 24.20 (4.30) | 90.0 (30) | 20 | 12 | 17 | 0 |
|  | Roc0.90 | Roc0.90 |  |  |  | 7.40 (1.50) | 22.50 (8.60) | 174 (21) | 20 | 17 | 20 | 0 |
|  | Roc1.20 | Roc1.20 |  |  |  | 6.30 (1.90) | 23.50 (7.50) | 180 (10) | 20 | 20 | 20 | 0 |
| 42. Karadeniz (2000) [S42] | CA0.15 | CA0.15 | no premedication | propofol 4 mg∙kg^-1^ | 0 | 9.80 (2.40) | 28.60 (9.70) | 120 | 20 | 18 | 20 | 0 |
|  | Atr0.50 | Atr0.50 |  |  |  | 8.30 !3.90) | 25.70 (8.90) | 120 | 20 | 16 | 18 | 2 |
| 43. Klemola (2000)¶ [S43] | Roc0.40 | Roc0.45 | atr 15 µg∙kg^-1^ iv; and  mdz 0.50 mg∙kg^-1^ po | propofol 3.5 mg∙kg^-1^. | 1 | 5.40 (1.90) | 21.90 (5.30) | 60 | 20 | 7 | 20 | 0 |
|  | Roc0.20 | Roc0.20 |  | remifentanil 2 µg∙kg^-1^; propofol 3.5 mg∙kg^-1^. |  | 5.30 (1.80) | 21.60 (4.60) | 60 | 20 | 13 | 19 | 0 |
|  | NO | PL-Op |  | remifentanil 2 µg∙kg^-1^; propofol 3.5 mg∙kg^-1^. |  | 5.70 (1.90) | 21.10 (4.60) | 60 | 20 | 11 | 18 | 0 |
|  | NO | PL-Op |  | remifentanil 4 µg∙kg^-1^; propofol 3.5 mg∙kg^-1^. |  | 4.80 (1.40) | 20.8 (5.40) | 60 | 20 | 18 | 20 | 0 |
| 44. Kulkarni (2010)& [S44] | Sux1.50 | Sux1.50 | glp 4µg∙kg^-1^ im; and  mdz 0.50 mg∙kg^-1^ im | thiopentone 6-8 mg∙kg^-1^. | 1 | 4.00 (2.50) | 17.00 (5.07) | 60 | 100 | 90 | 100 | 0 |
|  | Roc0.60 | Roc0.60 |  |  |  | 4.00 (2.53) | 17.00 (5.30) | 60 | 100 | 40 | 92 | 0 |
|  | Roc0.90 | Roc0.90 |  |  |  | 4.50 (2.60) | 18.00 (5.40) | 60 | 100 | 82 | 100 | 0 |
| 45. Kumar (2023)†& [S45] | Roc1.20 | Roc1.20 | mdz 0.01 mg∙kg^-1^ iv; and  glp 10µg∙kg^-1^ iv | fentanyl 2 µg∙kg^-1^; and propofol 2 mg∙kg^-1^ | 1 | 6.61 (4.25) | 17.92 (8.73) | 45 | 100 | 65 | 90 | 0 |
|  | Sux2.00 | Sux2.00 |  |  |  | 5.24 (3.17) | 15.61 (6.61) | 45 | 100 | 60 | 80 | 0 |
| 46. Lekmanov (1998)& [S46] | Miv0.20 | Miv0.20 | atr 20 µg∙kg^-1^ iv; diazepam 0.20 mg∙kg^-1^ and ketamine 6 mg∙kg^-1^ im. | fentanyl 1 µg∙kg^-1^ and DHBP 0.5 mg∙kg^-1^ | 0 | 5.99 (2.66) | NA | 89 (17) | 25 | 16 | 25 | 0 |
|  | Atr0.50 | Atr0.50 |  |  |  | 7.56 (3.17) | NA | 108 (36) | 16 | 12 | 16 | 0 |
| 47. Lysakowski (2000)¶& [S47] | Miv0.20 | Miv0.20 | diazepam  0.30-0.50 mg∙kg^-1^ ir | alfentanil 15 µg∙kg^-1^; and propofol 3 mg∙kg^-1^ | 1 | 5.00 (2.50) | 20.00 (6.00) | 120 | 13 | 7 | 12 | 0 |
|  | Vec0.14 | Vec0.10 |  |  |  | 5.00 (3.00) | 21.00 (8.50) | 120 | 15 | 11 | 15 | 0 |
| 48a. Malhotra (2002)¶& [S48a] | Roc0.90 | Roc0.90 | no premedication if <1y  atr 10 µg∙kg^-1^ iv; and  morphine 0.30 mg∙kg^-1^ im | sevoflurane 7-8% IC in N_2_O: 50%; mdz 0.10 mg∙kg^-1^;  and morphine 0.10 mg∙kg^-1^; | 1 | 5.50 (6.50) | 11.50 (0.80) | 60 | 20 | 16 | 20 | 0 |
|  | Vec0.20 | Vec0.20 |  |  |  | 6.00 (8.40) | 12.20 (0.80) | 60 | 20 | 8 | 19 | 1 |
| 48b. Malhotra (2002)¶& [S48b] | Roc0.90 | Roc0.90 |  |  |  | 5.50 (6.50) | 11.50 (0.80) | 90 | 20 | 18 | 20 | 0 |
|  | Vec0.20 | Vec0.20 |  |  |  | 6.00 (8.40) | 12.20 (0.80) | 90 | 20 | 16 | 19 | 0 |
| 49. Mangat (1993)¶& [S49] | Miv0.20 | Miv0.20 | trimeprazine 2-3 mg∙kg^-1^po | thiopentone 4-6 mg∙kg^-1^; and halothane 1-1.5% in N_2_O 67%. | 1 | 5.80 (1.90) | 22.90 (6.20). | 93 (14) | 32 | 20 | 31 | 1 |
|  | Sux1.00 | Sux1.00 |  |  |  | 5.80 (1.80) | 20.70 (4.40) | 43 (6) | 32 | 22 | 32 | 0 |
| 50. Mazurek (1998)†& [S50] | Sux1.50 | Sux1.50 | atr 10 µg∙kg^-1^ iv | thiopentone 5 mg∙kg^-1^ | 1 | 6.40 (4.20) | 26.80 (16.40) | 42 (3) | 13 | 10 | 12 | 0 |
|  | Roc1.20 | Roc1.20 |  |  |  | 6.80 (2.40) | 29.20 (14.20) | 40 (4) | 13 | 7 | 12 | 0 |
| 51. Mikailu (2023)† [S51] | NO | PL-Op | atr 20 µg∙kg^-1^ iv, and  mdz 0.05 mg∙kg^-1^ iv | propofol 3 mg∙kg^-1^; lido 0.20 mg∙kg^-1^; and fentanyl 3 µg∙kg^-1^ | 1 | 7.00 (3.32) | NA | 120 | 42 | 9 | 42 | 0 |
|  | Sux1.50 | Sux1.50 |  | propofol 3 mg∙kg^-1^; and lido 0.20 mg∙kg^-1^ |  | 6.52 (2.75) | NA | 120 | 42 | 36 | 42 | 0 |
| 52. Módolo (2002)& [S52] | Roc0.90 | Roc0.90 | mdz 0.30-0.50 mg∙kg^-1^ po | alfentanil 50 µg∙kg^-1^; and propofol 3 mg∙kg-1 in N_2_O 50%. | 1 | 5.93 (3.04) | 23.13 (11.65) | 36 | 22 | NA | 22 | 0 |
|  | Atr0.50 | Atr0.50 |  |  |  | 5.15 (2.91) | 19.58 (7.71) | 78 | 22 | NA | 22 | 0 |
|  | Miv0.15 | Miv0.15 |  |  |  | 5.46 (2.78) | 19.85 (9.46) | 116 | 22 | NA | 22 | 0 |
| 53. Montgomery (1988)& [S53] | Atr0.40 | Atr0.50 | no premedication | thiopentone 4-6 mg∙kg^-1^; and halothane 1.5% IC in N_2_O 60%. | 1 | 4.00 (1.30) | 16.30 (2.60) | 300 | 16 | 15 | 15 | 0 |
|  | Vec0.07 | Vec0.10 |  |  |  | 3.90 (1.50) | 16.40 (3.00) | 300 | 17 | 15 | 17 | 0 |
| 54. Morgan (2007)¶ [S54] | Sux1.00 | Sux1.00 | no premedication | propofol 4 mg∙kg^-1^; and isoflurane 2% IC. | 1 | 8.60 (3.50) | 34.00 (16.00) | 60 | 30 | NA | 26 | 0 |
|  | NO | PL-Op |  | propofol 4 mg∙kg^-1^; remifentanil 1.25 µg∙kg^-1^; and isoflurane 2% IC. |  | 8.80 (3.50) | 32.00 (14.70) | 60 | 30 | NA | 20 | 5 |
| 55. Mortazavi (2010)¶ [S55] | NO | PL-Op | no premedication | remifentanil 1 µg∙kg^-1^; propofol 3 mg∙kg^-1^ | 1 | 6.15 (2.68) | NA | 100 | 15 | NA | 5 | 2 |
|  | NO | PL-Op |  | remifentanil 2 µg∙kg^-1^; propofol 3 mg∙kg^-1^ |  | 6.15 (2.68) | NA | 100 | 15 | NA | 6 | 0 |
|  | NO | PL-Op |  | remifentanil 3 µg∙kg^-1^; propofol 3 mg∙kg^-1^ |  | 6.15 (2.68) | NA | 100 | 15 | NA | 9 | 0 |
|  | Atr0.50 | Atr0.50 |  | propofol 3 mg∙kg^-1^ |  | 6.15 (2.68) | NA | 100 | 15 | NA | 12 | 0 |
| 56. Nadirsha (2023)†¶ [S56] | Atr0.50 | Atr0.50 | glp 4µg∙kg^-1^ iv; and  mdz 0.03 mg∙kg^-1^ im | fentanyl 2 µg∙kg-1; and propofol 2 mg∙kg^-1^ | 1 | 6.13 (2.56) | 19.50 (6.46) | 180 | 52 | 28 | 48 | 4 |
|  | CA0.10 | CA0.10 |  |  |  | 5.48 (2.21) | 17.70 (5.92) | 180 | 52 | 10 | 41 | 11 |
| 57. Naguib (1997)¶& [S57] | Sux1.00 | Sux1.00 | trimeprazine 2 mg∙kg^-1^ po | fentanyl 2 µg∙kg^-1^; and propofol 2 mg∙kg^-1^ | 1 | 5.10 (1.25) | 20.10 (4.10) | 60 | 10 | 9 | 10 | 0 |
|  | Miv0.20 | Miv0.20 |  |  |  | 5.70 (1.75) | 24.00 (10.10) | 60 | 10 | 5 | 9 | 0 |
|  | Roc0.60 | Roc0.60 |  |  |  | 4.40 (1.00) | 16.70 (2.80) | 60 | 10 | 7 | 10 | 0 |
|  | Roc0.90 | Roc0.90 |  |  |  | 5.50 (1.25) | 22.80 (6.50) | 60 | 10 | 10 | 10 | 0 |
| 58. Nava-Ocampo (2001)†& [S58] | Miv0.20 | Miv0.20 | no premedication | atr 20 µg∙kg^-1^; fentanyl 2 µg∙kg^-1^; and propofol 3 mg∙kg^-1^ | 1 | 1.50 (0.53) | 11.10 (2.20) | 120 (36) | 12 | 12 | 12 | 0 |
|  | Miv0.25 | Miv0.25 |  |  |  | 1.27 (0.53) | 10.30 (2.30) | 96 (36) | 12 | 12 | 12 | 0 |
| 59. Naziri (2015)† [S59] | NO | PL-Op | mdz 0.05 mg∙kg^-1^ iv; and  lido 1.50 mg∙kg^-1^ iv | propofol 4 mg∙kg^-1^; remifentanil 2 µg∙kg^-1^; and N_2_O 60%. | 1 | 5.88 (2.65) | 19.76 (7.59) | 60 | 30 | 26 | 30 | 0 |
|  | Sux1.50 | Sux1.50 |  | propofol 3 mg∙kg^-1^; and N_2_O 60%. |  | 6.76 (2.13) | 19.43 (5.78) | 90 | 30 | 30 | 30 | 0 |
| 60. Ng (1990)¶ [S60] | NO | PL-Op | no premedication | halothane 2-3% IC; alfentanil 20 µg∙kg^-1^; and N_2_O 60%. | 1 | 4.60 (1.31) | 15.30 (4.36) | 60 | 20 | 15 | 18 | 2 |
|  | Sux2.00 | Sux2.00 |  | halothane 2-3% IC; and N_2_O 60%. |  | 4.70 (1.74) | 17.50 (4.92) | 60 | 20 | 15 | 20 | 0 |
| 61. Öztekin (2004)† [S61] | Roc0.15 | Roc0.15 | mdz 0.50 mg∙kg^-1^ po and  atr 10 µg∙kg^-1^ iv. | remifentanil 0.50 µg∙kg∙min^-1^ and propofol 2.5 mg∙kg^-1^. | 1 | 7.50 (1.90) | 24.10 (6.40) | 90 | 22 | 4 | 13 | 9 |
|  | Roc0.30 | Roc0.30 |  |  |  | 6.80 (2.30) | 22.80 (5.40) | 90 | 22 | 9 | 21 | 1 |
| 62a. Papagiannopoulou (2008) ¶& [S62a] | Roc0.60 | Roc0.60 | mdz 0.50 mg∙kg^-1^ po | mdz 0.03 mg∙kg^-1^; fentanyl 4 mg∙kg^-1^; and propofol 2-3 mg∙kg^-1^ | 1 | 2.35 (5.80) | 14.90 (3.80) | 60 | 10 | 10 | 10 | 0 |
|  | Miv0.20 | Miv0.20 |  |  |  | 2.47 (5.60) | 14.30 (4.10) | 60 | 10 | 8 | 9 | 0 |
| 62b. Papagiannopoulou (2008) ¶& [S62b] | Roc0.60 | Roc0.60 |  |  |  | 9.70 (3.90) | 39.10 (14.30) | 60 | 10 | 9 | 10 | 0 |
|  | Miv0.20 | Miv0.20 |  |  |  | 9.50 (3.50) | 41.30 (19.60) | 60 | 10 | 8 | 10 | 0 |
| 63. Park (2021)†¶& [S63] | NO | PL | no premedication | propofol 2 mg∙kg^-1^; and sevoflurane 5% IC. | 1 | 3.40 (1.60) | 15.50 (3.90) | 90 | 37 | 22 | 35 | 0 |
|  | NO | RF-Op |  | alfentanil 14 µg∙kg^-1^; and sevoflurane 5% IC. |  | 3.20 (1.20) | 15.00 (3.80) | 90 | 36 | 35 | 36 | 0 |
|  | Roc0.30 | Roc0.30 |  | sevoflurane 5% IC. |  | 3.40 (1.50) | 15.10 (3.90) | 90 | 38 | 33 | 38 | 0 |
| 64. .Pineda Diaz (1996) [S64] | Roc0.60 | Roc0.60 | no premedication | atr 10 µg∙kg^-1^; fentanyl 3 mg∙kg^-1^; propofol 2.5 mg∙kg^-1^ | 1 | 8.45 (4.09) | 30.13 (17.72) | 71 (20) | 60 | 59 | 60 | 0 |
|  | Atr0.60 | Atr0.50 |  |  |  | 8.45 (4.09) | 30.13 (17.72) | 162 (22) | 60 | 45 | 60 | 0 |
|  | Vec0.10 | Vec0.10 |  |  |  | 8.45 (4.09) | 30.13 (17.72) | 132 (44) | 60 | 49 | 59 | 1 |
| 65. Politis (2005)¶ [S65] | NO | PL | mdz 0.50 – 0.60 mg∙kg^-1^ po | halothane 3% IC | 1 | 3.80 (3.00) | 13.80 (5.50) | 180 | 19 | NA | 17 | 2 |
|  | Roc0.25 | Roc0.30 |  |  |  | 2.80 (2.50) | 12.70 (6.10) | 180 | 23 | NA | 20 | 2 |
| 66a. Rapp (2004)& [S66a] | Roc0.45 | Roc0.45 | no premedication | thiopentone 5-7 mg∙kg^-1^; and isoflurane 1% ET | 1 | 0.05 (0.02) | 3.44 (0.49) | 60 | 10 | 6 | 10 | 0 |
|  | Roc0.60 | Roc0.60 |  |  |  | 0.05 (0.03) | 3.66 (0.52) | 60 | 10 | 7 | 10 | 0 |
| 66b. Rapp (2004)& [S66b] | Roc0.45 | Roc0.45 |  |  |  | 0.17 (0.06) | 4.20 (1.27) | 60 | 9 | 6 | 7 | 0 |
|  | Roc0.60 | Roc0.60 |  |  |  | 0.20 (0.07) | 4.81 (1.53) | 60 | 12 | 5 | 9 | 0 |
| 66c. Rapp (2004)& [S66c] | Roc0.45 | Roc0.45 | mdz 0.40 mg∙kg^-1^ ir; and  atr 20 µg∙kg^-1^ iv |  |  | 0.74 (0.25) | 8.71 (0.98) | 60 | 10 | 7 | 10 | 0 |
|  | Roc0.60 | Roc0.60 |  |  |  | 0.62 (0.23) | 8.36 (1.22) | 60 | 10 | 8 | 10 | 0 |
| 67. Rizvanovic (2017)†¶ [S67] | Sux1.00 | Sux1.00 | mdz 0.50 mg∙kg^-1^ po.; and atr 10 µg∙kg^-1^ iv | propofol 3 mg∙kg^-1^; and lido 0.20 mg∙kg^-1^ | 1 | 8.00 (2.00) | NA | 60 | 40 | 39 | 40 | 0 |
|  | NO | PL-Op |  | fentanyl 3 µg∙kg^-1^; propofol 3 mg∙kg^-1^; and lido 0.20 mg∙kg^-1^ |  | 8.00 (2.00) | NA | 360 | 40 | 34 | 38 | 2 |
| 68. Rodney (1992) [S68] | NO | PL | atr 20 µg∙kg^-1^ iv | propofol 3.5 mg∙kg^-1^. | 1 | 4.50 (1.25) | NA | 40 | 25 | 0 | 0 | 4 |
|  | NO | PL-Op |  | alfentanil 20 µg∙kg^-1^; and propofol 3.5 mg∙kg^-1^. |  | 4.50 (1.25) | NA | 40 | 25 | 5 | 8 | 1 |
|  | Sux2.00 | Sux2.00 |  | thiopentone 5 mg∙kg^-1^ |  | 4.50 (1.25) | NA | 50 | 25 | 22 | 24 | 0 |
| 69. Salawu (2017)† [S69] | Sux1.50 | Sux1.50 | atr 10 µg∙kg^-1^ iv | fentanyl 2 µg∙kg^-1^; propofol 2.5 mg∙kg^-1^; and lido 0.20 mg∙kg^-1^ | 1 | 5.90 (2.20) | 22.80 (8.00) | 91 (30) | 33 | 28 | 33 | 2 |
|  | NO | PL |  | fentanyl 2 µg∙kg^-1^-sevoflurane 5% IC in N_2_O 60%. |  | 5.60 (2.00) | 21.10 (6.30) | 219 (64) | 33 | 15 | 31 | 3 |
| 70a. Said-Ahmed (2006)¶& [S70a] | NO | PL | mdz 0.50 mg∙kg^-1^ po | propofol 3 mg∙kg-1 | 1 | 5.00 (1.00) | 19.00 (6.00) | 120 | 20 | NA | 5 |  |
|  | Roc0.15 | Roc0.15 |  |  |  | 5.00 (1.00) | 19.00 (6.00) | 120 | 20 | NA | 15 |  |
|  | Roc0.22 | Roc0.20 |  |  |  | 5.00 (1.00) | 19.00 (6.00) | 120 | 20 | NA | 17 |  |
|  | Roc0.30 | Roc0.30 |  |  |  | 5.00 (1.00) | 19.00 (6.00) | 120 | 20 | NA | 19 |  |
|  | Roc0.60 | Roc0.60 |  |  |  | 5.00 (1.00) | 19.00 (6.00) | 120 | 20 | NA | 20 |  |
| 70b. Said-Ahmed (2006)¶& [S70b] | NO | PL |  | sevoflurane 8% IC in N2O 60% |  | 4.58 (1.25) | 19.00 (6.00) | 120 | 20 | NA | 7 |  |
|  | Roc0.15 | Roc0.15 |  |  |  | 4.58 (1.25) | 20.00 (4.00) | 120 | 20 | NA | 16 |  |
|  | Roc0.22 | Roc0.20 |  |  |  | 4.58 (1.25) | 20.00 (4.00) | 120 | 20 | NA | 18 |  |
|  | Roc0.30 | Roc0.30 |  |  |  | 4.58 (1.25) | 20.00 (4.00) | 120 | 20 | NA | 19 |  |
|  | Roc0.60 | Roc0.60 |  |  |  | 4.58 (1.25) | 20.00 (4.00) | 120 | 20 | NA | 20 |  |
| 71. Schreiber (1996)& [S71] | Roc0.60 | Roc0.60 | mdz 0.50 mg∙kg^-1^ po | atr 10 µg∙kg^-1^; etomidate 0.2-0.4 mg∙kg^-1^; fentanyl 1-3 mg∙kg^-1^; and N_2_O 50%. | 1 | 3.75 (1.13) | NA | 60 | 20 | 20 | 20 | 0 |
|  | Atr0.50 | Atr0.50 |  |  |  | 3.75 (1.13) | NA | 60 | 20 | 1 | 10 | 5 |
|  | Vec0.10 | Vec0.10 |  |  |  | 3.75 (1.13) | NA | 60 | 20 | 5 | 14 | 1 |
| 72. Schulz (1998) [S72] | Sux2.00 | Sux2.00 | atr 10 µg∙kg^-1^ iv | thiopentone 5 mg∙kg^-1^; and N_2_O 70%. | 1 | 4.50 (1.25) | NA | 30 | 18 | 12 | 17 | 0 |
|  | Roc1.20 | Roc1.20 |  |  |  | 4.50 (1.25) | NA | 30 | 14 | 6 | 13 | 0 |
| 73. Senel (1999) [S73] | NO | PL-Op | no premedication | propofol 2.5 mg∙kg^-1^; and alfentanil 20 µg∙kg^-1^ | 1 | 9.60 (2.97) | 28.47 (7.18) | 90 | 15 | NA | 12 | 2 |
|  | Atr0.50 | Atr0.50 |  | propofol 2.5 mg∙kg^-1^ |  | 8.53 (2.97) | 23.60 (6.62) | 90 | 15 | NA | 15 | 0 |
| 74. Shaginyan (2008)& [S74] | Sux1.00 | Sux1.00 | mdz 0.30 mg∙kg^-1^ im; and  atr 10 µg∙kg^-1^ im. | halothane 0.8% IC; fentanyl 2 µg∙kg^-1^; and N_2_O 60%. | 0 | 9.80 (0.50) | 32.40 (1.80) | 53 (12) | 25 | 18 | 25 | 0 |
|  | Roc0.30 | Roc0.30 |  |  |  | 11.10 (1.20) | 45.00 (5.60) | 89 (9) | 20 | 19 | 20 | 0 |
|  | Roc0.60 | Roc0.60 |  |  |  | 10.80 (0.90) | 44.60 (3.60) | 62 (5) | 20 | 20 | 20 | 0 |
|  | Roc0.90 | Roc0.90 |  |  |  | 11.20 (0.80) | 45.50 (3.50) | 43 (4) | 20 | 20 | 20 | 0 |
| 75. Shaikh (2010)†¶ [S75] | NO | PL-Op | mdz 0.05 mg∙kg^-1^; and  atr 10 µg∙kg^-1^ iv | fentanyl 4 µg∙kg^-1^; propofol 3 mg∙kg^-1^; and lido 0.20 mg∙kg^-1^ | 1 | 7.90 (2.80) | 19.70 (6.13) | 60 | 40 | 14 | 38 | 1 |
|  | Sux1.00 | Sux1.00 |  | propofol 3 mg∙kg^-1^; and lido 0.20 mg∙kg^-1^ |  | 8.80 (2.40) | 21.00 (5.50) | 60 | 40 | 36 | 40 | 0 |
| 76. Shakeet (2021) [S76] | Roc0.60 | Roc0.60 | Glp 4 µg∙kg^-1^; ranitidine 1 mg∙kg^-1^; odansetron 80 µg∙kg^-1^; and tramadol 1 mg∙kg^-1^ | thiopentone 5 mg∙kg^-1^ | 0 | 6.82 (1.43) | NA | 90 | 50 | 47 | 50 | 0 |
|  | Sux2.00 | Sux2.00 |  |  |  | 7.10 (1.86) | NA | 90 | 50 | 50 | 50 | 0 |
| 77. Shangguan (2007)& [S77] | CA0.10 | CA0.10 | ketamine 2 mg∙kg^-1^;  atr 10-20 µg∙kg^-1^; mdz 0.10 mg∙kg^-1^ | propofol 2 mg∙kg^-1^; and fentanyl 3 µg∙kg^-1^ | 1 | 2.33 (0.75) | 12.90 (2.90) | 238 | 15 | 11 | 15 | 0 |
|  | CA0.15 | CA0.15 |  |  |  | 2.25 (1.08) | 13.20 (2.60) | 140 | 15 | 15 | 15 | 0 |
|  | CA0.20 | CA0.20 |  |  |  | 2.25 (0.67) | 13.70 (4.10) | 130 | 15 | 15 | 15 | 0 |
| 78. Shorten (1996)& [S78] | Sux2.00 | Sux2.00 | no premedication | propofol 2.5 mg∙kg^-1^; atr10 µg∙kg^-1^; fentanyl 2-4 µg∙kg^-1^ in N_2_O 70%. | 1 | 6.10 (2.10) | 21.70 (6.50) | 36 (6) | 12 | NA | 12 | 0 |
|  | Miv0.20 | Miv0.20 |  |  |  | 5.20 (1.20) | 20.90 (5.20) | 90 (12) | 12 | NA | 11 | 0 |
|  | Miv0.30 | Miv0.30 |  |  |  | 7.10 (2.60) | 25.50 (8.50) | 72 (12) | 12 | NA | 12 | 0 |
|  | Miv0.40 | Miv0.40 |  |  |  | 5.10 (2.10) | 19.20 (8.40) | 60 (12) | 12 | NA | 12 | 0 |
| 79. Shrey (2020)†& [S79] | Atr0.50 | Atr0.50 | mdz 0.05 mg∙kg^-1^ iv; glp 4 µg∙kg^-1^ iv; ranitidine 10 mg iv; and odansetron 0.10 mg∙kg^-1^ iv | fentanyl 1 mg∙kg^-1^; propofol 1.5-2 mg∙kg^-1^. | 1 | 4.80 (0.89) | NA | 197 (38) | 45 | 15 | 41 | NA |
|  | CA0.20 | CA0.20 |  |  |  | 4.60 (0.68) | NA | 162 (7) | 45 | 18 | 43 | NA |
| 80. Sloan (1991)¶& [S80] | Sux2.00 | Sux2.00 | no premedication | atr 20 2.5 µg∙kg-1; thiopentone 2.5 mg∙kg^-1^; diazepam 0.15 mg∙kg^-1^ | 1 | 3.83 (1.11) | 16.70 (2.00) | 48 (10) | 10 | 10 | 10 | 0 |
|  | Vec0.10 | Vec0.10 |  |  |  | 5.40 (2.43) | 20.40 (9.10) | 109 (26) | 10 | 10 | 10 | 0 |
|  | Vec0.20 | Vec0.20 |  |  |  | 3.81 (1.36) | 16.20 (2.50) | 81 (21) | 10 | 10 | 10 | 0 |
|  | Vec0.40 | Vec0.40 |  |  |  | 4.65 (1.42) | 18.00 (4.60) | 57 (13) | 10 | 10 | 10 | 0 |
| 81. Söğüt (2000)& [S81] | Roc0.60 | Roc0.60 | atr 15 µg∙kg^-1^; and  mdz 0.06 mg∙kg^-1^ im. | sevoflurane 5% IC in N_2_O 50% | 0 | 5.01 (1.63) | 18.35(4.29) | 92.14 | 20 | 17 | 20 | 0 |
|  | Miv0.20 | Miv0.20 |  |  |  | 5.17 (1.46) | 18.45(4.73) | 126.43 | 20 | 20 | 20 | 0 |
|  | CA0.10 | CA0.10 |  |  |  | 5.11 (1.69) | 17.84(4.54) | 167.14 | 20 | 18 | 20 | 0 |
| 82. Srivastava (2001)¶ [S82] | NO | PL-Op | no premedication | atr 10 µg∙kg^-1^; fentanyl 1 µg∙kg^-1^; propofol 4 mg∙kg^-1^ lido 0.20 mg∙kg^-1^ | 1 | 7.95 (3.01) | 19.53 (7.07) | 60 | 40 | 10 | 31 | 7 |
|  | Sux1.00 | Sux1.00 |  | thiopentone 3-5 mg∙kg^-1^ |  | 8.57 (2.37) | 18.03 (5.80) | 60 | 30 | 27 | 29 | 1 |
| 83. Steyn (1994)†¶ [S83] | Placebo | RF-Op | no premedication | alfentanil 15 µg∙kg^-1^; propofol 4 mg∙kg^-1^; and lido 0.20 mg∙kg^-1^ | 1 | 6.83 (2.60) | 23.02 (10.75) | 45 | 40 | NA | 32 | 0 |
|  | Sux1.50 | Sux1.50 |  | propofol 4 mg∙kg^-1^; and lido 0.20 mg∙kg^-1^ |  | 7.08 (2.63) | 23.62 (8.90) | 45 | 40 | NA | 35 | 0 |
| 84. Stoddart (1998)¶& [S84] | Sux1.00 | Sux1.00 | paracetamol 20 mg∙kg^-1^. | propofol 3-4 mg∙kg^-1^. | 1 | 7.50 (3.00) | 27.00 (15.60) | 60 | 30 | 25 | 30 | 0 |
|  | Roc0.60 | Roc0.60 |  |  |  | 7.50 (3.30) | 26.70 (10.70) | 60 | 30 | 27 | 30 | 0 |
| 85. Tartari (1990)& [S85] | Vec0.05 | Vec0.05 | diazepam 0.20 mg∙kg^-1^ ir; and atr 10 µg∙kg^-1^ iv. | thiopentone 5 mg∙kg^-1^; | 0 | 6.60 (3.60) | 23.00 (10.0) | 218 (55) | 11 | 3 | 7 | 0 |
|  | Vec0.10 | Vec0.10 |  |  |  | 9.50 (4.30) | 35.00 (17.00) | 135 (18) | 11 | 8 | 11 | 0 |
|  | Vec0.15 | Vec0.20 |  |  |  | 9.80 (4.00) | 37.00 (15.00) | 98 (21) | 9 | 8 | 9 | 0 |
|  | Vec0.05 | Vec0.05 |  | isoflurane |  | 5.50 (2.70) | 30.00 (13.00) | 148 (40) | 10 | 8 | 10 | 0 |
| 86. Thwaites (1999)†¶ [S86] | Sux2.00 | Sux2.00 | ibuprofen 4 mg∙kg^-1^ po. | propofol 3-4 mg∙kg^-1^; morphine 0.1 mg∙kg^-1^; and N_2_O 67%. | 1 | 5.00 (1.75) | 21.00 (9.00) | 150 | 33 | 27 | 33 | 0 |
|  | NO | PL-Op |  | sevoflurane 8% IC; morphine 0.1 mg∙kg^-1^; and N_2_O 67%. |  | 5.00 (1.50) | 20.20 (8.88) | 150 | 31 | 17 | 31 | 0 |
| 87. Ved (1989)& [S87] | Atr0.25 | Atr0.25 | no premedication | halothane 1.5% in N_2_O 60%. | 1 | 4.60 (2.88) | 19.3 (7.27) | 234 (38) | 10 | NA | 10 | NA |
|  | Atr0.40 | Atr0.50 |  |  |  | 4.90 (2.21) | 20.4 (8.22) | 90 (38) | 10 | NA | 10 | NA |
| 88. Villegas (1999)¶& [S88] | Roc0.40 | Roc0.45 | atr 10 µg∙kg^-1^ iv | fentanyl 2 µg∙kg^-1^; propofol 3 mg∙kg^-1^. | 1 | 6.33 (4.15) | 23.46 (2.98) | 83 (24) | 15 | 13 | 15 | 0 |
|  | Roc0.60 | Roc0.60 |  |  |  | 9.33 (3.90) | 35.00 (17.12) | 88 (43) | 15 | 15 | 15 | 0 |
|  | Roc0.80 | Roc0.90 |  |  |  | 8.60 (3.73) | 30.31 (17.08) | 70 (34) | 15 | 15 | 15 | 0 |
| 89. Voss (2002)†& [S89] | Atr0.50 | Atr0.50 | mdz 0.40 mg∙kg^-1^ po | alfentanil 15; thiopentone 5-7 mg∙kg-1 | 1 | 7.00 (2.50) | NA | 120 | 42 | 30 | 41 | 0 |
|  | CA0.10 | CA0.10 |  |  |  | 7.00 (2.50) | NA | 120 | 42 | 20 | 29 | 3 |
| 90. Wei (2008) [S90] | NO | PL-Op | mdz 0.10 mg∙kg^-1^ im; and atr 10 µg∙kg^-1^ im. | sevoflurane 3% IC in N_2_O 60%.; and remifentanil 1 µg∙kg^-1^. | 1 | 6.60 (1.90) | 23.30 (7.60) | 60 | 30 | NA | 24 | 0 |
|  | NO | PL-Op |  | sevoflurane 3% IC in N2O 60%.; and remifentanil 2 µg∙kg^-1^. |  | 6.60 (1.80) | 23.00 (5.10) | 60 | 30 | NA | 26 | 0 |
|  | NO | PL-Op |  | sevoflurane 3% IC in N_2_O 60%.; and remifentanil 3 µg∙kg^-1^. |  | 7.20 (1.60) | 23.90 (6.10) | 60 | 30 | NA | 27 | 0 |
|  | Vec0.10 | Vec0.10 |  | sevoflurane 3% IC in N_2_O 60%. |  | 6.80 (1.60) | 22.60 (5.90) | 120 | 30 | NA | 28 | 0 |
| 91. Wei (2010)& [S91] | CA0.10 | CA0.10 | no premedication | mdz 0.20 mg∙kg-1; fentanyl 2 µg∙kg^-1^; and propofol 2-4 mg∙kg^-1^. | 1 | 4.00 (1.60) | 17.00 (3.70) | 254 (62) | 15 | 14 | 15 | 0 |
|  | CA0.15 | CA0.15 |  |  |  | 4.40 (1.50) | 19.00 (4.00) | 174 (39) | 15 | 15 | 15 | 0 |
|  | CA0.20 | CA0.20 |  |  |  | 3.60 (1.60) | 16.50 (3.00) | 162 (30) | 15 | 15 | 15 | 0 |
| 92. Wei (2011)& [S92] | CA0.10 | CA0.10 | no premedication | mdz 0.20 mg∙kg^-1^; fentanyl 2 µg∙kg^-1^; propofol 2-4 mg∙kg^-1^ and N_2_O 50%. | 1 | 4.60 (1.50) | 17.90 (5.90) | 60 | 20 | 4 | 19 | 0 |
|  | CA0.15 | CA0.15 |  |  |  | 5.30 (1.60) | 18.90 (5.20) | 60 | 20 | 15 | 20 | 0 |
|  | CA0.20 | CA0.20 |  |  |  | 5.00 (1.60) | 18.40 (5.40) | 60 | 20 | 16 | 20 | 0 |
| 93. Woloszczuk(1998)& [S93] | Roc0.80 | Roc0.90 | mdz 0.30 mg∙kg^-1^ iv | thiopentone 7.5 mg∙kg^-1^; fentanyl 1 µg∙kg^-1^; and N_2_O 70%. | 0 | 7.00 (2.00) | NA | 39 | 10 | 8 | 10 | 0 |
|  | Roc0.60 | Roc0.60 |  |  |  | 7.00 (2.00) | NA | 90 | 10 | 8 | 9 | 0 |
| 94. Wu (2023)†¶& [S94] | NO | PL | no premedication | Sevoflurane 4% IC; and remifentanil 2 µg∙kg-1. | 1 | 0.12 (0.11) | 4.50 (1.00) | 90 | 35 | NA | 23 | 5 |
|  | Roc0.45 | Roc0.45 |  |  |  | 0.14 (0.10) | 4.40 (0.90) | 90 | 33 | NA | 31 | 2 |
|  | Roc0.60 | Roc0.60 |  |  |  | 0.21 (0.07) | 4.50 (0.90) | 90 | 34 | NA | 33 | 1 |
| 95a. Zeng (2017)¶& [S95a] | Miv0.15 | Miv0.15 | ketamin 1-2 mg∙kg^-1^;  mdz 0.10-0.20 mg∙kg^-1^;  and atr 20 µg∙kg^-1^ | mdz 0.10-0.20 mg∙kg^-1^; fentanyl 3-5 µg∙kg^-1^;  propofol 2-3 mg∙kg^-1^ followed by 50-100 µg∙kg^-1^·min^-1^;  remifentanil 0.30-0.50 µg∙kg^-1^·min^-1^ | 1 | 0.92 (0.83) | 8.50 (2.40) | 220 (73) | 39 | 29 | 39 | 0 |
|  | Miv0.20 | Miv0.20 |  |  |  | 0.92 (0.83) | 9.10 (2.40) | 189 (64) | 39 | 37 | 39 | 0 |
| 95b. Zeng (2017)¶& [S95b] | Miv0.15 | Miv0.15 |  |  |  | 0.92 (0.83) | 8.60 (2.20) | 213 (71) | 37 | 29 | 37 | 0 |
|  | Miv0.20 | Miv0.20 |  |  |  | 0.92 (0.83) | 8.80 (2.00) | 181 (60) | 40 | 38 | 40 | 0 |
| 95c. Zeng (2017)¶& [S95c] | Miv0.20 | Miv0.20 |  |  |  | 1.58 (0.46) | 13.10 (6.10) | 173 (51) | 29 | 27 | 29 | 0 |
|  | Miv0.25 | Miv0.25 |  |  |  | 1.58 (0.46) | 14.30 (7.20) | 184 (60) | 28 | 24 | 28 | 0 |
| 95d. Zeng (2017)¶& [S95d] | Miv0.20 | Miv0.20 |  |  |  | 1.58 (0.46) | 11.50 (2.10) | 180 (56) | 25 | 22 | 25 | 0 |
|  | Miv0.25 | Miv0.25 |  |  |  | 1.58 (0.46) | 12.60 (3.10) | 167 (52) | 24 | 24 | 24 | 0 |
| 95e. Zeng (2017)¶& [S95e] | Miv0.20 | Miv0.20 |  |  |  | 4.50 (0.75) | 20.30 (5.20) | 183 (57) | 34 | 27 | 34 | 0 |
|  | Miv0.25 | Miv0.25 |  |  |  | 4.50 (0.75) | 19.80 (6.50) | 161 (51) | 35 | 32 | 35 | 0 |
| 95f. Zeng (2017)¶& [S95f] | Miv0.20 | Miv0.20 |  |  |  | 4.50 (0.75) | 19.10 (5.90) | 172 (55) | 36 | 35 | 36 | 0 |
|  | Miv0.25 | Miv0.25 |  |  |  | 4.50 (0.75) | 17.90 (5.50) | 160 (50) | 36 | 30 | 36 | 0 |
| 95g. Zeng (2017)¶& [S95g] | Miv0.20 | Miv0.20 |  |  |  | 10.50 (1.75) | 31.10 (11.30) | 184 (57) | 40 | 36 | 40 | 0 |
|  | Miv0.25 | Miv0.25 |  |  |  | 10.50 (1.75) | 33.70 (11.80) | 181 (58) | 40 | 39 | 40 | 0 |
| 95h. Zeng (2017)¶& [S95h] | Miv0.20 | Miv0.20 |  |  |  | 10.50 (1.75) | 34.60 (17.20) | 187 (66) | 40 | 35 | 38 | 0 |
|  | Miv0.25 | Miv0.25 |  |  |  | 10.50 (1.75) | 32.20 (13.10) | 182 (56) | 40 | 37 | 40 | 0 |
| 96. Hung (2005)¶ [S96] | Roc0.30 | Roc0.30 | no premedication | sevoflurane 4% IC in N_2_O 50%. | 1 | 2.55 (0.63) | 13.40 (2.39) | 60 | 20 | 19 | 20 | 0 |
|  | Roc0.30 | Roc0.30 |  |  |  | 2.21 (0.60) | 12.65 (2.00) | 90 | 20 | 20 | 20 | 0 |
|  | Roc0.30 | Roc0.30 |  |  |  | 5.18 (0.69) | 18.40 (2.70) | 60 | 20 | 13 | 20 | 0 |
|  | Roc0.30 | Roc0.30 |  |  |  | 5.15 (0.61) | 18.10 (3.34) | 90 | 20 | 16 | 20 | 0 |
| 97. Hadimioglu (2005)¶& [S97] | Miv0.20 | Miv0.20 | mdz 0.50 mg∙kg^-1^ po | lido 10 mg; fentanyl 1-2 µg∙kg^-1^; propofol 2 mg∙kg^-1^ and 100-120 µg∙kg^-1^∙min^-1^ | 1 | 6.90 (2.90) | 24.60 (9.80) | 186 (114) | 30 | 16 | 30 | 0 |
|  | Miv0.20 | Miv0.20 |  | sevoflurane 8% IC; and fentanyl 1-2 µg∙kg^-1^ |  | 5.80 (3.30) | 22.40 (8.60) | 108 (48) | 30 | 18 | 30 | 0 |
| 98. Kocabaş (2002) [S98] | Roc0.60 | Roc0.60 | no premedication | atr 10 µg∙kg^-1^; fentanyl 2 µg∙kg^-1^; thiopentone 5 mg∙kg^-1^; and  halothane 1-1.5% in N_2_O 67% | 1 | 5.08 (1.89) | 19.85 (6.79) | 76 (26) | 20 | 17 | 20 | 0 |
|  | Roc0.60 | Roc0.60 |  | atr 10 µg∙kg-1; fentanyl 2 µg∙kg^-1^; propofol 2 mg∙kg^-1^ and N_2_O 67% |  | 7.10 (3.50) | 25.05 (9.09) | 106 (58) | 20 | 18 | 20 | 0 |
| 99a. Kenaan (2000)& [S99a] | CA0.20 | CA0.20 | mdz 0.10-0.20 mg∙kg^-1^ ins | halothane 3% IC in N_2_O 60-70% | 1 | 3.46 (1.46) | 14.90 | 90 | 6 | 6 | 6 | 0 |
|  | CA0.20 | CA0.20 |  |  |  | 8.96 (3.96) | 27.40 | 90 | 15 | 11 | 14 | 0 |
| 99b. Kenaan (2000)& [S99b] | CA0.20 | CA0.20 |  | fentanyl 2 µg∙kg^-1^; and thiopentone 5 mg∙kg^-1^ |  | 3.46 (1.46) | 16.20 | 90 | 6 | 6 | 6 | 0 |
|  | CA0.20 | CA0.20 |  |  |  | 8.96 (3.96) | 28.40 | 90 | 15 | 10 | 15 | 0 |
| 100. Meakin (2007)†& [S100] | CA0.15 | CA0.15 | <6M:mdz 0.20-0.30 mg∙kg^-1^ ins  or mdz 0.50-0.70 mg∙kg^-1^ po | halothane ≥ 3% IC in N_2_O 60-70% | 1 | 0.58 (0.40) | 8.00 (2.72) | 120 | 30 | 30 | 30 | 0 |
|  | CA0.15 | CA0.15 |  |  |  | 3.42 (1.76) | 16.00 (3.40) | 120 | 30 | 27 | 29 | 0 |
|  | CA0.15 | CA0.15 |  |  |  | 6.92 (2.10) | 25.00 (6.80) | 120 | 30 | 22 | 29 | 0 |
|  | CA0.15 | CA0.15 |  | fentanyl 2 µg∙kg^-1^; and thiopentone 5 mg∙kg^-1^; in N_2_O 60-70% |  | 0.58 (0.28) | 9.00 (2.04) | 120 | 30 | 25 | 30 | 0 |
|  | CA0.15 | CA0.15 |  |  |  | 2.42 (1.81) | 13.00 (4.08) | 120 | 31 | 20 | 27 | 0 |
|  | CA0.15 | CA0.15 |  |  |  | 7.50 (2.27) | 26.00 (5.44) | 120 | 29 | 20 | 28 | 0 |
| 101. Hopkinson (1997)¶& [S101] | Roc0.60 | Roc0.60 | mdz 0.50 mg∙kg^-1^ po | thiopentone 6-8 mg∙kg^-1^; and alfentanil 50 µg∙kg^-1^ | 1 | 6.90 (2.60) | 23.50 (7.40) | 30 | 12 | 0 | 4 | 1 |
|  | Roc0.60 | Roc0.60 |  |  |  | 6.60 (3.10) | 24.30 (8.60) | 40 | 12 | 0 | 6 | 1 |
|  | Roc0.60 | Roc0.60 |  |  |  | 6.30 (2.10) | 22.40 (6.50) | 50 | 12 | 1 | 8 | 0 |
|  | Roc0.60 | Roc0.60 |  |  |  | 5.50 (2.10) | 20.70 (8.00) | 60 | 12 | 7 | 11 | 0 |
|  | Roc0.60 | Roc0.60 |  |  |  | 6.60 (3.70) | 25.10 (11.4) | 70 | 12 | 9 | 12 | 0 |
| 102. Lee (2010)¶ [S102] | Roc0.60 | Roc0.60 | pethidine 1 mg∙kg^-1^ im | propofol 2.5 mg∙kg-1; and lido 1.00 mg∙kg^-1^ | 1 | 4.60 (2.60) | 20.10 (8.90) | 80 | 31 | 27 | 31 | 0 |
|  | Roc0.60 | Roc0.60 |  |  |  | 5.50 (2.20) | 33.00 (10.10) | 60 | 36 | 21 | 34 | 1 |
| 103. McCluskey (1996)¶& [S103] | Miv0.20 | Miv0.20 | mdz 0.30-0.50 mg∙kg^-1^ po | thiopentone 6-8 mg∙kg-1; in N2O 67% | 1 | 5.50 (2.25) | NA | 60 | 50 | 13 | 33 | 9 |
|  | Miv0.20 | Miv0.20 |  |  |  | 5.50 (2.25) | NA | 90 | 50 | 38 | 49 | 0 |
| 104. McDonald (1997)¶& [S104] | Roc0.60 | Roc0.60 | no premedication | fentanyl 2 µg∙kg^-1^; and thiopentone 6 mg∙kg^-1^; | 0 | 5.50 (1.90) | 22.00 (6.80) | 50 | 15 | 11 | 14 | 0 |
|  | Roc0.60 | Roc0.60 |  |  |  | 5.90 (1.80) | 23.20 (7.40) | 60 | 15 | 12 | 15 | 0 |
| 105. Shimhi (1997)¶& [S105] | Miv0.30 | Miv0.30 | mdz 0.25-0.30 mg∙kg^-1^ ins | atr 10-20 µg∙kg^-1^; and halothane 3 % IC | 1 | 3.75 (1.32) | 15.00 (3.49) | 45 (4) | 15 | 7 | 9 | 3 |
|  | Miv0.30 | Miv0.30 |  |  |  | 4.50 (1.26) | 18.00 (3.87) | 48 (17) | 15 | 3 | 8 | 2 |
|  | Miv0.30 | Miv0.30 |  |  |  | 4.17 (1.32) | 17.00 (4.65) | 88 (10) | 15 | 7 | 12 | 1 |
|  | Miv0.30 | Miv0.30 |  |  |  | 4.58 (1.13) | 18.00 (3.20) | 84 (19) | 15 | 7 | 14 | 0 |
|  |  |  |  |  |  |  |  |  |  |  |  |  |

**Table S1.** The analysis includes 105 studies (94 RCTs and 11 CCTs) with 315 study arms and 8008 paediatric participants, examining neuromuscular block (NMBA) interventions, procedural aspects, and patient outcomes related to intubation conditions and treatment effectiveness. Treatment groups received either NMBA (specified by agent and dose in mg∙kg^-1^ iv) or a placebo (NO). Placebo groups were further categorized as NMBA-free (placebo) without opioids (PL) or NMBA-free (placebo) with opioids (PL-Op). A node represents a unit where similar treatments are grouped. Key variables include time to intubation (t_int) in seconds, total (n): number of participants per study arm exposed; EIC(n), AIC(n), and Fail (n): numbers of participants in study arm exhibiting either, excellent, acceptable or failed intubation, respectively.

The studied NMBA agents include atracurium (Atr), cis-atracurium (CA), mivacurium (Miv), rocuronium (Roc), suxamethonium (Sux), and vecuronium (Vec), along with adjunct medications such as atropine (atr), glycopyrrolate (glp), midazolam (mdz), lidocaine (lido), and dehydrobenzperidol (DHBP). Routes of administration include peroral (po), intravenous (iv), intranasal (ins), and rectal (ir), with reported inspiratory (IC) and end-tidal (ET) concentrations. NA: not available, indicates missing values; † marks sample size calculations conducted before study initiation; ¶ explicitly mentions the experience level or seniority of the intubating professional; & use of neuromuscular transmission monitoring prior to intubation; ‡ marks the NMBA use of either suxamethonium (2 mg∙kg^-1^), vecuronium (0.1-0.2 mg∙kg^-1^), rocuronium (0.6-1.0 mg∙kg^-1^), or atracurium (0.4-0.5 mg∙kg^-1^)

**Supplementary material File 3b: Blood pressure and heart rate before and at 1 and 3 minutes after intubation.**

| **Author** | **Treatment** | **Node** | SAP0 | MAP0 | DAP0 | HR0 | SAP1 | MAP1 | DAP1 | HR1 | SAP3 | MAP3 | DAP3 | HR3 |
| --- | --- | --- | --- | --- | --- | --- | --- | --- | --- | --- | --- | --- | --- | --- |
| 1. An (2003) [S1] | Atr0.50 | Atr0.50 | NA | 82.0 (8.9) | NA | 103.8 (3.0) | NA | 88.4 (6.3) | NA | 118.1 (4.4) | NA | 84.0 (4.4) | NA | 121.2 (7.2) |
|  | Vec0.10 | Vec0.10 | NA | 81.7 (7.0) | NA | 101.9 (4.3) | NA | 85.2 (8.7) | NA | 104.5 (4.1) | NA | 86.9 (3.7) | NA | 112.6 (5.0) |
|  | Roc0.60 | Roc0.60 | NA | 87.7 (7.0) | NA | 101.1 (4.2) | NA | 90.5 (9.1) | NA | 116.6 (5.4) | NA | 92.9 (6.0) | NA | 118.5 (2.2) |
| 2. Annila (1999) [S2] | Sux1.50 | Sux1.50 | NA | 102 (22) | NA | 124 | NA | 96 (38) | NA | 112 | NA | 61 | NA | 104 |
|  | NO | PL-Op | NA | 78 (22) | NA | 102 | NA | 86 | NA | 97 | NA | 52 | NA | 94 |
|  | NO | PL | NA | 74 (22) | NA | 99 | NA | 55 | NA | 98 | NA | 56 | NA | 99 |
| 3. Apilioğulları (2006) [S3] | Miv0.20 | Miv0.20 | NA | NA | NA | NA | NA | NA | NA | NA | NA | NA | NA | NA |
|  | Roc0.60 | Roc0.60 | NA | NA | NA | NA | NA | NA | NA | NA | NA | NA | NA | NA |
| 4. Bala (2024)† [S4] | CA0.15 | CA0.15 | NA | 68.2 (8.2) | NA | 111.3 (14.2) | NA | 70.6 (9.4) | NA | 113.7 (11.8) | NA | 65.9 (8.2) | NA | 116.1 (14.2) |
|  | Atr0.50 | Atr0.50 | NA | 68.2 (8.2) | NA | 111.3 (14.2) | NA | 68.9 (7.1) | NA | 113.7 (11.7) | NA | 65.9 (8.2) | NA | 116.1 (14.2) |
| 5. Balakrishnan (1999) [S5] | Miv0.20 | Miv0.20 | NA | NA | NA | NA | NA | NA | NA | NA | NA | NA | NA | NA |
|  | Atr0.50 | Atr0.50 | NA | NA | NA | NA | NA | NA | NA | NA | NA | NA | NA | NA |
| 6. Bansal (2023)† [S6] | Atr0.50 | Atr0.50 | NA | NA | NA | NA | NA | NA | NA | NA | NA | NA | NA | NA |
|  | Roc0.60 | Roc0.60 | NA | NA | NA | NA | NA | NA | NA | NA | NA | NA | NA | NA |
| 7. Bartolek (2010)† [S7] | Vec0.10 | Vec0.10 | NA | NA | NA | NA | NA | NA | NA | NA | NA | NA | NA | NA |
|  | Roc0.60 | Roc0.60 | NA | NA | NA | NA | NA | NA | NA | NA | NA | NA | NA | NA |
|  | Roc0.45 | Roc0.45 | NA | NA | NA | NA | NA | NA | NA | NA | NA | NA | NA | NA |
| 8. Barve (2002) [S8] | Roc0.60 | Roc0.60 | NA | NA | NA | NA | NA | NA | NA | NA | NA | NA | NA | NA |
|  | Sux1.00 | Sux1.00 | NA | NA | NA | NA | NA | NA | NA | NA | NA | NA | NA | NA |
| 9. Bhattacharya (2008) [S9] | Vec0,10 | Vec0,10 | NA | NA | NA | NA | NA | NA | NA | NA | NA | NA | NA | NA |
|  | Roc0,60 | Roc0,60 | NA | NA | NA | NA | NA | NA | NA | NA | NA | NA | NA | NA |
|  | Roc0,90 | Roc0,90 | NA | NA | NA | NA | NA | NA | NA | NA | NA | NA | NA | NA |
| 10. Blair (2000)† [S10] | Sux1.00 | Sux1.00 | NA | NA | NA | 119.5 (22.3) | NA | NA | NA | NA | NA | NA | NA | NA |
|  | NO | PL-Op | NA | NA | NA | 97.6 (22.8) | NA | NA | NA | NA | NA | NA | NA | NA |
|  | NO | PL | NA | NA | NA | 115.0 (27.8) | NA | NA | NA | NA | NA | NA | NA | NA |
| 11. Blair (2004)† [S11] | Miv0.20 | Miv0.20 | NA | NA | NA | NA | NA | NA | NA | NA | NA | NA | NA | NA |
|  | NO | RF-Op | NA | NA | NA | NA | NA | NA | NA | NA | NA | NA | NA | NA |
|  | NO | RF-Op | NA | NA | NA | NA | NA | NA | NA | NA | NA | NA | NA | NA |
|  | NO | PL | NA | NA | NA | NA | NA | NA | NA | NA | NA | NA | NA | NA |
| 12a. Bock (2007)† [S12a] | Roc0.45 | Roc0.45 | NA | NA | NA | NA | NA | NA | NA | NA | NA | NA | NA | NA |
|  | Roc0.60 | Roc0.60 | NA | NA | NA | NA | NA | NA | NA | NA | NA | NA | NA | NA |
| 12b. Bock (2007)† [S12b] | Roc0.45 | Roc0.45 | NA | NA | NA | NA | NA | NA | NA | NA | NA | NA | NA | NA |
|  | Roc0.60 | Roc0.60 | NA | NA | NA | NA | NA | NA | NA | NA | NA | NA | NA | NA |
| 13. Bucx (1994) [S13] | Sux1.50 | Sux1.50 | NA | NA | NA | 114 | NA | NA | NA | 106 | NA | NA | NA | 108 |
|  | Vec0.10 | Vec0.10 | NA | 117 | NA | 81 | NA | 111 | NA | 79 | NA | 100 | NA | 74 |
| 14. Castillo-Peralta (2004) [S14]. | Roc0.30 | Roc0.30 | NA | NA | NA | NA | NA | NA | NA | NA | NA | NA | NA | NA |
|  | Roc0.60 | Roc0.60 | NA | NA | NA | NA | NA | NA | NA | NA | NA | NA | NA | NA |
|  | Roc0.90 | Roc0.90 | NA | NA | NA | NA | NA | NA | NA | NA | NA | NA | NA | NA |
| 15. Chee (1998) [S15] | Miv0.20 | Miv0.20 | NA | NA | NA | NA | NA | NA | NA | NA | NA | NA | NA | NA |
|  | Miv0.25 | Miv0.25 | NA | NA | NA | NA | NA | NA | NA | NA | NA | NA | NA | NA |
| 16. Cheng (2002) [S16] | Vec0.10 | Vec0.10 | NA | NA | NA | 114 | NA | NA | NA | 106 | NA | NA | NA | 108 |
|  | Roc0.60 | Roc0.60 | NA | 117 | NA | 81 | NA | 111 | NA | 79 | NA | 100 | NA | 74 |
|  | Roc0.90 | Roc0.90 | NA | NA | NA | NA | NA | NA | NA | NA | NA | NA | NA | NA |
| 17. Cheng (2002)† [S17] | Sux1.50 | Sux1.50 | NA | NA | NA | NA | NA | NA | NA | NA | NA | NA | NA | NA |
|  | Roc0.90 | Roc0.90 | NA | NA | NA | NA | NA | NA | NA | NA | NA | NA | NA | NA |
|  | Roc0.60 | Roc0.60 | NA | NA | NA | NA | NA | NA | NA | NA | NA | NA | NA | NA |
| 18a. Cook (1995)† [S18a] | Miv0.15 | Miv0.15 | 91 (7) | 65 (5) | 73 (7) | 148 (19) | 93 (6) | 64 (10) | 44 (10) | 156 (9) | 99 (7) | 71 (10) | 50 (12) | 166 (9) |
|  | Sux1.60 | Sux1.50 | NA | NA | NA | NA | NA | NA | NA | NA | NA | NA | NA | NA |
| 18b. Cook (1995)† [S18b] | Miv0.20 | Miv0.20 | 92 (12) | 70 (6) | 53 (10) | 111 (23) | 97 (13) | 70 (10) | 51 (10) | 114 (21) | 104 (28) | 78 (9) | 59 (12) | 123 (28) |
|  | Sux0.90 | Sux1.00 | NA | NA | NA | NA | NA | NA | NA | NA | NA | NA | NA | NA |
| 18c. Cook (1995)† [S18c] | Miv0.30 | Miv0.30 | 85 (11) | 62 (17) | 44 (10) | 102 (20) | 84 (10) | 58 (13) | 43 (10) | 115 (16) | 84 (7) | 60 (8) | 41 (7) | 111 (13) |
|  | Sux1.50 | Sux1.50 | NA | NA | NA | 149 | NA | NA | NA | NA | NA | NA | NA | NA |
| 19. Cook-Sather (1998) [S19] | NO | PL | NA | NA | NA | 195 (17) | NA | NA | NA | 192 (29) | NA | NA | NA | NA |
|  | Sux2.00 | Sux2.00 | NA | NA | NA | 187 (20) | NA | NA | NA | 177 (29) | NA | NA | NA | NA |
|  | NMBA | NMBA | NA | NA | NA | 185 (15) | NA | NA | NA | 179 (22) | NA | NA | NA | NA |
| 20. Crawford (2005)† [S20] | Sux2.00 | Sux2.00 | NA | 77 (16) | NA | 157 (28) | NA | 86 (16) | NA | 159 (16) | NA | 74 (16) | NA | 167 (16) |
|  | Placebo | RF-Op | NA | 77 (16) | NA | 149 (25) | NA | 71 (16) | NA | 142 (16) | NA | 65 (16) | NA | 152 (8) |
| 21. Deepak (2024)† [S21] | CA0.15 | CA0.15 | NA | 88.9 | NA | 106.5 | NA | 88.9 | NA | 104.4 | NA | 86.7 | NA | 106.6 |
|  | Roc0.60 | Roc0.60 | NA | 88.4 | NA | 104.9 | NA | 86.4 | NA | 107.0 | NA | 86.7 | NA | 104.9 |
| 22. Desai (2019) [S22] | Roc0.90 | Roc0.90 | NA | NA | NA | NA | NA | NA | NA | NA | NA | NA | NA | NA |
|  | Sux1.50 | Sux1.50 | NA | NA | NA | NA | NA | NA | NA | NA | NA | NA | NA | NA |
| 23. Devys (2011)† [S23] | NO | PL | NA | 59 (10) | NA | 127 (15) | NA | NA | NA | NA | NA | NA | NA | NA |
|  | NO | PL-Op | NA | 58 (10) | NA | 124 (18) | NA | NA | NA | NA | NA | NA | NA | NA |
|  | Roc0.30 | Roc0.30 | NA | 61 (12) | NA | 132 (18) | NA | NA | NA | NA | NA | NA | NA | NA |
| 24. Eikermann (2001)† [S24] | NO | PL | NA | NA | NA | NA | NA | NA | NA | NA | NA | NA | NA | NA |
|  | Roc0.15 | Roc0.15 | NA | NA | NA | NA | NA | NA | NA | NA | NA | NA | NA | NA |
|  | Roc0.22 | Roc0.20 | NA | NA | NA | NA | NA | NA | NA | NA | NA | NA | NA | NA |
|  | Roc0.33 | Roc0.30 | NA | NA | NA | NA | NA | NA | NA | NA | NA | NA | NA | NA |
|  | Roc0.50 | Roc0.45 | NA | NA | NA | NA | NA | NA | NA | NA | NA | NA | NA | NA |
|  | Roc1.00 | Roc0.90 | NA | NA | NA | NA | NA | NA | NA | NA | NA | NA | NA | NA |
| 25. Eikermann (2002)† [S25] | NO | PL | NA | NA | NA | NA | NA | NA | NA | NA | NA | NA | NA | NA |
|  | Roc0.15 | Roc0.15 | NA | NA | NA | NA | NA | NA | NA | NA | NA | NA | NA | NA |
|  | Roc0.22 | Roc0.20 | NA | NA | NA | NA | NA | NA | NA | NA | NA | NA | NA | NA |
|  | Roc0.30 | Roc0.30 | NA | NA | NA | NA | NA | NA | NA | NA | NA | NA | NA | NA |
|  | Roc0.60 | Roc0.60 | NA | NA | NA | NA | NA | NA | NA | NA | NA | NA | NA | NA |
| 26. Fang (2015) [S26] | Miv0.20 | Miv0.20 | NA | 76.3 (12.4) | NA | 103.2 (15.4) | NA | NA | NA | NA | NA | 73.2 (15.1) | NA | 101.3 (15.1) |
|  | CA0.10 | CA0.10 | NA | 76.1 (13.9) | NA | 102.8 (14.1) | NA | NA | NA | NA | NA | 78.2 (13.9) | NA | 101.5 (14.7) |
| 27. Fletcher (2004) [S27] | Roc0.40 | Roc0.45 | NA | NA | NA | NA | NA | NA | NA | NA | NA | NA | NA | NA |
|  | Miv0.133 | Miv0.15 | NA | NA | NA | NA | NA | NA | NA | NA | NA | NA | NA | NA |
| 28. Frediani (1993) [S28] | Vec0.04 | Vec0.05 | NA | NA | NA | NA | NA | NA | NA | NA | NA | NA | NA | NA |
|  | Atr0.20 | Atr0.25 | NA | NA | NA | NA | NA | NA | NA | NA | NA | NA | NA | NA |
| 29. Friesdorf (1986) [S29] | Vec0.08 | Vec0.075 | NA | NA | NA | NA | NA | NA | NA | NA | NA | NA | NA | NA |
|  | Vec0.10 | Vec0.10 | NA | NA | NA | NA | NA | NA | NA | NA | NA | NA | NA | NA |
| 30. Fuchs-Buder (1996) [S30] | Roc0.60 | Roc0.60 | NA | NA | NA | NA | NA | NA | NA | NA | NA | NA | NA | NA |
|  | Roc0.90 | Roc0.90 | NA | NA | NA | NA | NA | NA | NA | NA | NA | NA | NA | NA |
| 31. Gelberg (2014) [S31] | NO | PL-Op | NA | NA | NA | NA | NA | NA | NA | NA | NA | NA | NA | NA |
|  | Roc0.30 | Roc0.30 | NA | NA | NA | NA | NA | NA | NA | NA | NA | NA | NA | NA |
| 32. Gera (2015)† [S32] | NO | RF-Op | NA | 73 (15) | NA | 113 (12) | NA | 68 (9) | NA | 132 (10) | NA | 64 (6) | NA | 118 (6) |
|  | Roc0.60 | Roc0.60 | NA | 73 (115) | NA | 113 (12) | NA | 68 (9) | NA | 122 (10) | NA | 58 (6) | NA | 118 (6) |
| 33. Gnani (2017)† [S33] | Sux1.50 | Sux1.50 | 103 | NA | 73 | 111 | 120 | NA | 81 | 123 | 107 | NA | 75 | 115 |
|  | Roc0.90 | Roc0.90 | 100 | NA | 72 | 111 | 113 | NA | 81 | 122 | 102 | NA | 75 | 115 |
|  | Roc1.20 | Roc1.20 | 97 | NA | 70 | 112 | 113 | NA | 78 | 122 | 102 | NA | 72 | 113 |
| 34. Gonzalez (2008) [S34] | Atr0.50 | Atr0.50 | NA | NA | NA | NA | NA | NA | NA | NA | NA | NA | NA | NA |
|  | Vec0.10 | Vec0.10 | NA | NA | NA | NA | NA | NA | NA | NA | NA | NA | NA | NA |
| 35. Green (1998) [S35] | Miv0.20 | Miv0.20 | NA | NA | NA | NA | NA | NA | NA | NA | NA | NA | NA | NA |
|  | Sux2.00 | Sux2.00 | NA | NA | NA | NA | NA | NA | NA | NA | NA | NA | NA | NA |
| 36. Grubhofer (1993) [S36] | NO | PL | NA | 55 (14) | NA | 88 (12) | NA | 57 (12) | NA | 95 (16) | NA | 61 (28) | NA | 93 (21) |
|  | Are0.40 | Atr0.50 | NA | 56 (16) | NA | 80 (17) | NA | 60 (23) | NA | 98 (16) | NA | 52 (13) | NA | 87 (10) |
| 37a. Grundman (1991) [S37a] | Atr0.30 | Atr0.25 | 80.0 (13.7) | 55.0 (5.8) | 42.5 (5.4) | 154.5 (14.8) | 81.5 (13.1) | 55.2 (5.4) | 42.0 (4.8) | 154.0 (13.9) | 79.5 (14.3) | 54.2 (5.0) | 41.5 (2.4) | 153.0 (14.3) |
|  | Vec0.05 | Vec0.05 | 78.5 (16.6) | 53.8 (8.1) | 41.5 (8.8) | 153.0 (9.7) | 79.5 (14.9) | 66.2 (8.4) | 59.5 (10.1) | 154.0 (11.7) | 78.0 (15.4) | 53.3 (7.6) | 41.0 (8.4) | 153.0 (10.5) |
| 37b. Grundman (1991) [S37b] | Atr0.50 | Atr0.50 | 99.5 (10.3) | 72.5 (7.9) | 59.0 (10.2) | 126.0 (11.9) | 101.5 (12.2) | 61.8 (7.1) | 42.0 (8.8) | 125.1 (12.0) | 100.5 (10.9) | 73.5 (7.2) | 60.0 (9.4) | 128.5 (14.9) |
|  | Vec0.10 | Vec0.10 | 99.5 (10.3) | 64.5 (7.3) | 47.0 (9.7) | 129.0 (18.3) | 95.5 (11.1) | 63.2 (7.5) | 47.0 (9.7) | 129.5 (15.3) | 94.0 (13.2) | 62.3 (7.7) | 46.5 (9.4) | 127.0 (13.9) |
| 38. Hansen (1997) [S38] | NO | PL | 95 (11) | NA | NA | 107 (20) | 96 (11) | NA | NA | 146 (14) | 100 (14) | NA | NA | 148 (16) |
|  | Sux1.50 | Sux1.50 | 98 (11) | NA | NA | 120 (20) | 111 (16) | NA | NA | 160 (14) | 98 (13) | NA | NA | 154 (16) |
| 39a. Huang (2007) [S39a] | CA0.10 | CA0.10 | NA | NA | NA | NA | NA | NA | NA | NA | NA | NA | NA | NA |
|  | CA0.15 | CA0.15 | NA | NA | NA | NA | NA | NA | NA | NA | NA | NA | NA | NA |
| 39b. Huang (2007) [S39b] | CA0.10 | CA0.10 | NA | NA | NA | NA | NA | NA | NA | NA | NA | NA | NA | NA |
|  | CA0.15 | CA0.15 | NA | NA | NA | NA | NA | NA | NA | NA | NA | NA | NA | NA |
| 39c. Huang (2007) [S39c] | CA0.10 | CA0.10 | NA | NA | NA | NA | NA | NA | NA | NA | NA | NA | NA | NA |
|  | CA0.15 | CA0.15 | NA | NA | NA | NA | NA | NA | NA | NA | NA | NA | NA | NA |
| 40. Huh (2017)† [S40] | NO | PL-Op | NA | NA | NA | NA | NA | NA | NA | NA | NA | NA | NA | NA |
|  | Roc0.15 | Roc0.15 | NA | NA | NA | NA | NA | NA | NA | NA | NA | NA | NA | NA |
|  | Roc0.30 | Roc0.30 | NA | NA | NA | NA | NA | NA | NA | NA | NA | NA | NA | NA |
| 41. Kapdi (2020) [S41] | Roc0.60 | Roc0.60 | NA | NA | NA | NA | NA | NA | NA | NA | NA | NA | NA | NA |
|  | Roc0.90 | Roc0.90 | NA | NA | NA | NA | NA | NA | NA | NA | NA | NA | NA | NA |
|  | Roc1.20 | Roc1.20 | NA | NA | NA | NA | NA | NA | NA | NA | NA | NA | NA | NA |
| 42. Karadeniz (2000) [S42] | CA0.15 | CA0.15 | 105.0 (7.8) | 74.3 (4.2) | 59.0 (5.0) | 105 (17) | 119.8 (12.0) | 91.9 (8.9) | 78.0 (12.0) | 125. (9) | NA | NA | NA | NA |
|  | Atr0.50 | Atr0.50 | 110.0 (17) | 78.7 (9.3) | 63.0 (11) | 98 (21) | 117.0 (8.0) | 87.7 (9.1) | 73.0 (13.0) | 123 (14) | NA | NA | NA | NA |
| 43. Klemola (2000) [S43] | Roc0.40 | Roc0.45 | NA | 87 (15) | NA | 106 (22) | NA | 96 (14) | NA | 101 (25) | NA | NA | NA | NA |
|  | Roc0.20 | Roc0.20 | NA | 72 (8) | NA | 104 (20) | NA | 70 (6) | NA | 112 (20) | NA | NA | NA | NA |
|  | NO | PL-Op | NA | 60 (12) | NA | 96 (10) | NA | 58 (10) | NA | 100 (13) | NA | NA | NA | NA |
| 44. Kulkarni (2010) [S44] | Sux1.50 | Sux1.50 | NA | NA | NA | NA | NA | NA | NA | NA | NA | NA | NA | NA |
|  | Roc0.60 | Roc0.60 | NA | NA | NA | NA | NA | NA | NA | NA | NA | NA | NA | NA |
|  | Roc0.90 | Roc0.90 | NA | NA | NA | NA | NA | NA | NA | NA | NA | NA | NA | NA |
| 45. Kumar (2023)† [S45] | Roc1.20 | Roc1.20 | 99.7 (17.6) | 74.8 (9.0) | 62.3(10.3) | 98.6 (8.3) | 106.3 (18.5) | 76.6 (7.5) | 61.7 (6.5) | 110.8 (7.5) | NA | NA | NA | NA |
|  | Sux2.00 | Sux2.00 | 107.3(5.6) | 73.6 (3.8) | 56.7 (5.0) | 103.7 (8.7) | 105.0 (11.5) | 78.9 (7.0) | 65.8 (8.7) | 104.2 (8.0) | NA | NA | NA | NA |
| 46. Lekmanov (1998) [S46] | Miv0.20 | Miv0.20 | NA | NA | NA | NA | NA | NA | NA | NA | NA | NA | NA | NA |
|  | Atr0.50 | Atr0.50 | NA | NA | NA | NA | NA | NA | NA | NA | NA | NA | NA | NA |
| 47. Lysakowski (2000) [S47] | Miv0.20 | Miv0.20 | NA | NA | NA | NA | NA | NA | NA | NA | NA | NA | NA | NA |
|  | Vec0.14 | Vec0.15 | NA | NA | NA | NA | NA | NA | NA | NA | NA | NA | NA | NA |
| 48a. Malhotra (2002) [S48a] | Roc0.90 | Roc0.90 | NA | 88 (4.2) | NA | 78 (4.2) | NA | 92 (6.8) | NA | 79 (5.8) | NA | NA | NA | NA |
|  | Vec0.20 | Vec0.20 | NA | 86 (5.4) | NA | 79.8 (11.4) | NA | 98 (8.2) | NA | 78.6 (10.2) | NA | NA | NA | NA |
| 48b. Malhotra (2002) [S48b] | Roc0.90 | Roc0.90 | NA | 88 (4.2) | NA | 78 (4.2) | NA | 108 (6.4) | NA | 85 (4.4) | NA | NA | NA | NA |
|  | Vec0.20 | Vec0.20 | NA | 86 (5.4) | NA | 79.8 (11.4) | NA | 102 (2.4) | NA | 77.8 (4.6) | NA | NA | NA | NA |
| 49. Mangat (1993) [S49] | Miv0.20 | Miv0.20 | NA | NA | NA | NA | NA | NA | NA | NA | NA | NA | NA | NA |
|  | Sux1.00 | Sux1.00 | NA | NA | NA | NA | NA | NA | NA | NA | NA | NA | NA | NA |
| 50. Mazurek (1998)† [S50] | Sux1.50 | Sux1.50 | NA | NA | NA | NA | NA | NA | NA | NA | NA | NA | NA | NA |
|  | Roc1.20 | Roc1.20 | NA | NA | NA | NA | NA | NA | NA | NA | NA | NA | NA | NA |
| 51. Mikailu (2023)† [S51] | NO | PL-Op | NA | NA | NA | NA | NA | NA | NA | NA | NA | NA | NA | NA |
|  | Sux1.50 | Sux1.50 | NA | NA | NA | NA | NA | NA | NA | NA | NA | NA | NA | NA |
| 52. Módolo (2002) [S52] | Roc0.90 | Roc0.90 | NA | 69 (15) | NA | 84 (17) | NA | 77 (13) | NA | 89 (19) | NA | 73 (10) | NA | 90 (22) |
|  | Atr0.50 | Atr0.50 | NA | 64 (15) | NA | 82 (17) | NA | 73 (13) | NA | 92 (18) | NA | 69 (9) | NA | 89 (25) |
|  | Miv0.15 | Miv0.15 | NA | 73 (13) | NA | 91 (19) | NA | 78 (9) | NA | 106 (14) | NA | 75 (10) | NA | 101 (23) |
| 53. Montgomery (1988) [S53] | Atr0.40 | Atr0.50 | NA | NA | NA | NA | NA | NA | NA | NA | NA | NA | NA | NA |
|  | Vec0.07 | Vec0.075 | NA | NA | NA | NA | NA | NA | NA | NA | NA | NA | NA | NA |
| 54. Morgan (2007) [S54] | Sux1.00 | Sux1.00 | NA | NA | NA | NA | NA | NA | NA | NA | NA | NA | NA | NA |
|  | NO | PL-Op | NA | NA | NA | NA | NA | NA | NA | NA | NA | NA | NA | NA |
| 55. Mortazavi (2010 [S55] | NO | PL-Op | NA | NA | NA | NA | NA | NA | NA | NA | NA | NA | NA | NA |
|  | NO | PL-Op | NA | NA | NA | NA | NA | NA | NA | NA | NA | NA | NA | NA |
|  | NO | PL-Op | NA | NA | NA | NA | NA | NA | NA | NA | NA | NA | NA | NA |
|  | NO | PL-Op | NA | NA | NA | NA | NA | NA | NA | NA | NA | NA | NA | NA |
|  | Atr0.50 | Atr0.50 | NA | NA | NA | NA | NA | NA | NA | NA | NA | NA | NA | NA |
| 56. Nadirsha (2023)† [S56] | Atr0.50 | Atr0.50 | 97.2 (8.4) | 71.7 (9.0) | 57.3 (9.8) | 99.8 (17.3) | 110.9 (12.3) | 83.2 (11.4) | 68.9 (12.2) | 115.7 (17.7) | 104.5 (12.0) | 78.4 (10.0) | 63.4 (11.5) | 110.50 (17.51) |
|  | CA0.10 | CA0.10 | 99.1 (10.4) | 73.3 (8.9) | 59.8 (9.6) | 100.4 (16.3) | 115.0 (11.5) | 86.5 (10.2) | 73.3 (10.9) | 120.3 (13.4) | 110.2 (10.4) | 83.5 (9.4) | 69.8 (10.8) | 115.17 (12.86) |
| 57. Naguib (1997) [S57] | S1.00 | Sux1.00 | NA | NA | NA | NA | NA | NA | NA | NA | NA | NA | NA | NA |
|  | M0.20 | Miv0.20 | NA | NA | NA | NA | NA | NA | NA | NA | NA | NA | NA | NA |
|  | R0.60 | Roc0.60 | NA | NA | NA | NA | NA | NA | NA | NA | NA | NA | NA | NA |
|  | R0.90 | Roc0.90 | NA | NA | NA | NA | NA | NA | NA | NA | NA | NA | NA | NA |
| 58. Nava-Ocampo (2001)† [S58] | Miv0.20 | Miv0.20 | 92 (9) | NA | 51 (10) | 94 (18) | 96 (11) | NA | 51 (7) | 114 (10) | 93 (11) | NA | 50 (8) | 115 (8) |
|  | Miv0.25 | Miv0.25 | 93 (11) | NA | 53 (12) | 100 (18) | 97 (11) | NA | 56 (12) | 116 (9) | 96 (10) | NA | 54 (9) | 115 (12) |
| 59. Naziri (2015)† [S59] | NO | PL-Op | 99 (14) | NA | NA | 95 (11) | 100 (10) | NA | NA | 115 (12) | 97 (14) | NA | NA | 105 (11) |
|  | Sux1.50 | Sux1.50 | 97 (15) | NA | NA | 101 (11) | 107 (13) | NA | NA | 111 (12) | 102 (9) | NA | NA | 113 (11) |
| 60. Ng (1990) [S60] | NO | PL-Op | NA | 100 (34) | NA | 105 (13) | NA | 110 (35) | NA | 103 (13) | NA | 103 (34) | NA | 104 (13) |
|  | Sux2.00 | Sux2.00 | NA | 67 (32) | NA | 82 (9) | NA | 74 (32) | NA | 84 (9) | NA | 80 (32) | NA | 92 (9) |
| 61. Öztekin (2004)† [S61] | Roc0.15 | Roc0.15 | NA | 67.5 (8.3) | NA | 95.7 (18.2) | NA | 67.6 (10.7) | NA | 99.1 (17.2) | NA | 63.9 (10.4) | NA | 98 (16.2) |
|  | Roc0.30 | Roc0.30 | NA | 70.8 (12.1) | NA | 100.2 (15.8) | NA | 72.2 (13.2) | NA | 104.4 (17.9) | NA | 63.9 (10.4) | NA | 102 (13.5) |
| 62a. Papagiannopoulou (2008) [S62a] | Roc0.60 | Roc0.60 | NA | NA | NA | NA | NA | NA | NA | NA | NA | NA | NA | NA |
|  | Miv0.20 | Miv0.20 | NA | NA | NA | NA | NA | NA | NA | NA | NA | NA | NA | NA |
| 62b. Papagiannopoulou (2008) [S62b] | Roc0.60 | Roc0.60 | NA | NA | NA | NA | NA | NA | NA | NA | NA | NA | NA | NA |
|  | Miv0.20 | Miv0.20 | NA | NA | NA | NA | NA | NA | NA | NA | NA | NA | NA | NA |
| 63. Park (2021)† [S63] | NO | PL | NA | 63 (2) | NA | 127 (5) | NA | 66 (3) | NA | 145 (5) | NA | 60 (2) | NA | 144 (4) |
|  | NO | PL-Op | NA | 58 (2) | NA | 112 (6) | NA | 59 (2) | NA | 134 (5) | NA | 57 (2) | NA | 137 (4) |
|  | Roc0.30 | Roc0.30 | NA | 61 (2) | NA | 121 (5) | NA | 68 (2) | NA | 142 (5) | NA | 61 (2) | NA | 137 (4) |
| 64..Pineda Diaz (1996) [S64] | Roc 0.60 | Roc 0.60 | NA | NA | NA | NA | NA | NA | NA | NA | NA | NA | NA | NA |
|  | Atr0.60 | Atr0.50 | NA | NA | NA | NA | NA | NA | NA | NA | NA | NA | NA | NA |
|  | Vec0.10 | Vec0.10 | NA | NA | NA | NA | NA | NA | NA | NA | NA | NA | NA | NA |
| 65.Politis (2005) [65] | NO | PL | NA | NA | NA | NA | NA | NA | NA | NA | NA | NA | NA | NA |
|  | Roc0.25. | Roc0.30 | NA | NA | NA | NA | NA | NA | NA | NA | NA | NA | NA | NA |
| 66a. Rapp (2004) [S66a] | Roc0.45 | Roc0.45 | NA | NA | NA | NA | NA | NA | NA | NA | NA | NA | NA | NA |
|  | Roc0.60 | Roc0.60 | NA | NA | NA | NA | NA | NA | NA | NA | NA | NA | NA | NA |
| 66b. Rapp (2004) [S66b] | Roc0.45 | Roc0.45 | NA | NA | NA | NA | NA | NA | NA | NA | NA | NA | NA | NA |
|  | Roc0.60 | Roc0.60 | NA | NA | NA | NA | NA | NA | NA | NA | NA | NA | NA | NA |
| 66c. Rapp (2004) [S66c] | Roc0.45 | Roc0.45 | NA | NA | NA | NA | NA | NA | NA | NA | NA | NA | NA | NA |
|  | Roc0.60 | Roc0.60 | NA | NA | NA | NA | NA | NA | NA | NA | NA | NA | NA | NA |
| 67. Rizvanovic (2017)† [S67] | Sux1.00 | Sux1.00 | 121.5 (29) | 89.4 (18) | 73.3 (22) | 123.2 (18) | 119.9 (21) | NA | 71.1 (14) | 120.3 (16) | 112.0 (13) | NA | 67.0 (13) | 119.65 (14) |
|  | NO | PL-Op | 95.5 (29) | 67.9 (18) | 53.8 (22) | 107.3 (18) | 100.9 (21) | NA | 58.7 (14) | 110.1 (16) | 106.5 (13) | NA | 64.5 (13) | 116.50 (14) |
| 68. Rodney (1992) [S68] | NO | PL | NA | NA | NA | NA | NA | NA | NA | NA | NA | NA | NA | NA |
|  | NO | RF-Op | NA | NA | NA | NA | NA | NA | NA | NA | NA | NA | NA | NA |
|  | Sux2.00 | Sux2.00 | NA | NA | NA | NA | NA | NA | NA | NA | NA | NA | NA | NA |
| 69. Salawu (2017)† [S69] | S1.50 | Sux1.50 | NA | 79 | NA | 112 | NA | 85 | NA | 136 | NA | 78 | NA | 136 |
|  | NO | PL | NA | 76 | NA | 99 | NA | 76 | NA | 129 | NA | 71 | NA | 129 |
| 70a. Said-Ahmed (2006) [S70a] | NO | PL | NA | NA | NA | NA | 105 (10) | NA | NA | 113 (20) | 95 (14) | NA | NA | 130 (25) |
|  | Roc0.15 | Roc0.15 | NA | NA | NA | NA | 105 (10) | NA | NA | 113 (20) | 95 (14) | NA | NA | 130 (25) |
|  | Roc0.22 | Roc0.20 | NA | NA | NA | NA | 105 (10) | NA | NA | 113 (20) | 95 (14) | NA | NA | 130 (25) |
|  | Roc0.30 | Roc0.30 | NA | NA | NA | NA | 105 (10) | NA | NA | 113 (20) | 95 (14) | NA | NA | 130 (25) |
|  | Roc0.60 | Roc0.60 | NA | NA | NA | NA | 105 (10) | NA | NA | 113 (20) | 95 (14) | NA | NA | 130 (25) |
| 70a. Said-Ahmed (2006) [S70b] | NO | PL | NA | NA | NA | NA | 105 (10) | NA | NA | 113 (20) | 95 (14) | NA | NA | 130 (25) |
|  | Roc0.15 | Roc0.15 | NA | NA | NA | NA | 105 (10) | NA | NA | 113 (20) | 95 (14) | NA | NA | 130 (25) |
|  | Roc0.22 | Roc0.20 | NA | NA | NA | NA | 105 (10) | NA | NA | 113 (20) | 95 (14) | NA | NA | 130 (25) |
|  | Roc0.30 | Roc0.30 | NA | NA | NA | NA | 105 (10) | NA | NA | 113 (20) | 95 (14) | NA | NA | 130 (25) |
|  | Roc0.60 | Roc0.60 | NA | NA | NA | NA | 105 (10) | NA | NA | 113 (20) | 95 (14) | NA | NA | 130 (25) |
| 71. Schreiber (1996) [S71] | Roc0.60 | Roc0.60 | NA | NA | NA | NA | NA | NA | NA | NA | NA | NA | NA | NA |
|  | Atr0.50 | Atr0.50 | NA | NA | NA | NA | NA | NA | NA | NA | NA | NA | NA | NA |
|  | Vec0.10 | Vec0.10 | NA | NA | NA | NA | NA | NA | NA | NA | NA | NA | NA | NA |
| 72. Schulz (1998) [S72] | Sux2.00 | Sux2.00 | NA | NA | NA | NA | NA | NA | NA | NA | NA | NA | NA | NA |
|  | Roc1.20 | Roc1.20 | NA | NA | NA | NA | NA | NA | NA | NA | NA | NA | NA | NA |
| 73. Senel (1999) [S73] | NO | RF-Op | NA | 80 | NA | 104 | NA | 76 | NA | 112 | NA | 75 | NA | 99 |
|  | Atr0.50 | Atr0.50 | NA | 76 | NA | 115 | NA | 76 | NA | 114 | NA | 74 | NA | 102 |
| 74. Shaginyan (2008) [S74] | Sux1.00 | Sux1.00 | NA | 78.3 (2.8) | NA | 91.8 (4.1) | NA | 70.8 (4.9) | NA | 78.6 (6.2) | NA | 77.8 (4.4) | NA | 90.6 (5.8) |
|  | Roc0.30 | Roc0.30 | NA | 73.00 (2.8) | NA | 95.6 (4.4) | NA | 73.5 (2.9) | NA | 95.7 (4.1) | NA | 74.5 (3.2) | NA | 96.0 (3.5) |
|  | Roc0.60 | Roc0.60 | NA | 77.2 (3.8) | NA | 96.2 (4.3) | NA | 81.7 (3.4) | NA | 98.0 (3.8) | NA | 79.8 (1.9) | NA | 97. (4.6) |
|  | Roc0.90 | Roc0.90 | NA | 76.57 (4.6) | NA | 92.0 (2.0) | NA | 71.4 (3.1) | NA | 97.5 (3.3) | NA | 73.5 (2.5) | NA | 95.6 (2.5) |
| 75. Shaikh (2010)† [S75] | NO | PL-Op | 109.2 (27) | NA | NA | 103.5 (32) | 108.6 | NA | NA | 121.6 | 104.4 | NA | NA | 101.3 |
|  | Sux1.00 | Sux1.00 | 125.3 (27) | NA | NA | 122.5 (32) | 122.7 | NA | NA | 103.4 | 118.3 | NA | NA | 114.5 |
| 76. Shakeet (2021) [S76] | Roc0.60 | Roc0.60 | NA | NA | NA | NA | NA | NA | NA | NA | NA | NA | NA | NA |
|  | Sux2.00 | Sux2.00 | NA | NA | NA | NA | NA | NA | NA | NA | NA | NA | NA | NA |
| 77. Shangguan (2007) [S77] | CA0.10 | CA0.10 | NA | NA | NA | NA | NA | NA | NA | NA | NA | NA | NA | NA |
|  | CA0.15 | CA0.15 | NA | NA | NA | NA | NA | NA | NA | NA | NA | NA | NA | NA |
|  | CA0.20 | CA0.20 | NA | NA | NA | NA | NA | NA | NA | NA | NA | NA | NA | NA |
| 78. Shorten (1996) [S78] | Sux2.00 | Sux2.00 | NA | 82 (12) | NA | NA | NA | 85 (16) | NA | NA | NA | 75 (14) | NA | NA |
|  | Miv0.20 | Miv0.20 | NA | 76 (9) | NA | NA | NA | 73 (17) | NA | NA | NA | 84 (15) | NA | NA |
|  | Miv0.30 | Miv0.30 | NA | 71 (12) | NA | NA | NA | 85 (17) | NA | NA | NA | 79 (13) | NA | NA |
|  | Miv0.40 | Miv0.40 | NA | 71 (15) | NA | NA | NA | 77 (17) | NA | 108.8 (7.9) | NA | 78 (20) | NA | NA |
| 79. Shrey (2020)† [S79] | Atr 0.50 | Atr 0.50 | NA | 93.4 (8.9) | NA | 98.8 (8.9) | NA | 97.5 (5.7) | NA | 96.7 (5.9) | NA | NA | NA | NA |
|  | CA0.20 | CA0.20 | NA | 92.4 (4.8) | NA | 94.4 (7.3) | NA | 93.7 (8.12) | NA | NA | NA | NA | NA | NA |
| 80. Sloan (1991) [S80] | Sux2.00 | Sux2.00 | NA | NA | NA | NA | NA | NA | NA | NA | NA | NA | NA | NA |
|  | Vec0.10 | Vec0.10 | NA | NA | NA | NA | NA | NA | NA | NA | NA | NA | NA | NA |
|  | Vec0.20 | Vec0.20 | NA | NA | NA | NA | NA | NA | NA | NA | NA | NA | NA | NA |
|  | Vec0.40 | Vec0.40 | NA | NA | NA | NA | NA | NA | NA | NA | NA | NA | NA | NA |
| 81. Söğüt (2000) [S81] | Roc0.60 | Roc0.60 | NA | NA | NA | NA | NA | NA | NA | NA | NA | NA | NA | NA |
|  | Miv0.20 | Miv0.20 | NA | NA | NA | NA | NA | NA | NA | NA | NA | NA | NA | NA |
|  | CA0.10 | CA0.10 | NA | NA | NA | NA | NA | NA | NA | NA | NA | NA | NA | NA |
| 82. Srivastava (2001) [S82] | NO | PL-Op | 101.5 (13.9) | NA | NA | 99.9 (12.7) | 95.6 (11.2) | NA | NA | 93.1 (9.3) | NA | NA | NA | NA |
|  | Sux1.00 | Sux1.00 | 127.6 (9.9) | NA | NA | 119.1 (9.6) | 124.5 (8.9) | NA | NA | 113.9 (7.6) | NA | NA | NA | NA |
| 83. Steyn (1994)† [S83] | NO | PL-Op | NA | 86 (13) | NA | 118 (15) | NA | 67 (8) | NA | 97 (15) | NA | 61 (8) | NA | 94 (19) |
|  | Sux1.50 | Sux1.50 | NA | 67 (13) | NA | 97 (15) | NA | 88 (8) | NA | 122 (15) | NA | 70 (8) | NA | 112 (19) |
| 84. Stoddart (1998) [S84] | Sux1.00 | Sux1.00 | NA | NA | NA | NA | NA | NA | NA | NA | NA | NA | NA | NA |
|  | Roc0.60 | Roc0.60 | NA | NA | NA | NA | NA | NA | NA | NA | NA | NA | NA | NA |
| 85. Tartari (1990) [S85] | Vec0.05 | Vec0.05 | NA | NA | NA | NA | NA | NA | NA | NA | NA | NA | NA | NA |
|  | Vec0.10 | Vec0.10 | NA | NA | NA | NA | NA | NA | NA | NA | NA | NA | NA | NA |
|  | Vec0.15 | Vec0.15 | NA | NA | NA | NA | NA | NA | NA | NA | NA | NA | NA | NA |
|  | Vec0.05 | Vec0.05 | NA | NA | NA | NA | NA | NA | NA | NA | NA | NA | NA | NA |
| 86. Thwaites (1999)† [S86] | Sux2.00 | Sux2.00 | NA | NA | NA | NA | NA | NA | NA | NA | NA | NA | NA | NA |
|  | NO | PL-Op | NA | NA | NA | NA | NA | NA | NA | NA | NA | NA | NA | NA |
| 87. Ved (1989) [S87] | Atr0.25 | Atr0.25 | NA | NA | NA | NA | NA | NA | NA | NA | NA | NA | NA | NA |
|  | Atr0.40 | Atr0.50 | NA | NA | NA | NA | NA | NA | NA | NA | NA | NA | NA | NA |
| 88. Villegas (1999) [S88] | Roc0.40 | Roc0.45 | NA | NA | NA | NA | NA | NA | NA | NA | NA | NA | NA | NA |
|  | Roc0.60 | Roc0.60 | NA | NA | NA | NA | NA | NA | NA | NA | NA | NA | NA | NA |
|  | Roc0.80 | Roc0.90 | NA | NA | NA | NA | NA | NA | NA | NA | NA | NA | NA | NA |
| 89. Voss (2002)† [S89] | Atr0.50 | Atr0.50 | NA | NA | NA | NA | NA | NA | NA | NA | NA | NA | NA | NA |
|  | CA0.10 | CA0.10 | NA | NA | NA | NA | NA | NA | NA | NA | NA | NA | NA | NA |
| 90. Wei (2008) [S90] | NO | PL-Op | 89.5 (9.7) | 56.5 (6.1) | 40.0 (5.8) | 109.6 (17.7) | 93.7 (10.7) | 61.9 (5.7) | 46.0 (5.7) | 115.4 (15.4) | 94.5 (6.5) | 64.4 (6.5) | 49.3 (6.5) | 116.1 (13.1) |
|  | NO | PL-Op | 89.7 (10.1) | 59.2 (9.9) | 43.9 (3.9) | 105.0 (25.2) | 94.4 (9.3) | 63.9 (9.3) | 48.1 (4.3) | 119.9 (18.4) | 94.8 (7.4) | 64.7 (7.4) | 49.6 (7.4) | 113.8 (17.3) |
|  | NO | PL-Op | 86.4 (8.9) | 54.2 (6.9) | 38.1 (6.2) | 92.3 (12.8) | 85.5 (9.2) | 57.6 (7.2) | 42.1 (5.2) | 94.9 (14.2) | 91.5 (6.9) | 61.9 (6.9) | 47.1 (6.9) | 98.4 (23.3) |
|  | Vec0.10 | Vec0.10 | 100.8 (11.2) | 65.5 (7.0) | 48.2 (4.2) | 124.2 (13.4) | 110.1 (12.6) | 75.7 (12.7) | 58.5 (6.7) | 137.4 (11.4) | 104.2 (11.1) | 69.2 (11.1) | 51.7 (11.1) | 135.1 (12.6) |
| 91. Wei (2010) [S91] | CA0.10 | CA0.10 | 93.8 (5.5) | 65.4 (3.9) | 51.2 (5.2) | 87.9 (6.9) | 95.4 (6.0) | 66.3 (3.7) | 51.8 (4.6) | 88.2 (2.5) | NA | NA | NA | NA |
|  | CA0.15 | CA0.15 | 94.1 (6.1) | 66.0 (4.0) | 52.0 (5.2) | 85.5 (6.9) | 94.7 (5.4) | 65.6 (3.0) | 51.0 (3.6) | 89.3 (6.3) | NA | NA | NA | NA |
|  | CA0.20 | CA0.20 | 93.0 (5.5) | 64.4 (3.4) | 50.1 (4.3) | 89.3 (7.0) | 93.5 (5.0) | 65.5 (4.0) | 51.5 (5.5) | 88.2 (6.0) | NA | NA | NA | NA |
| 92. Wei (2011) [S92] | CA0.10 | CA0.10 | NA | NA | NA | NA | NA | NA | NA | NA | NA | NA | NA | NA |
|  | CA0.15 | CA0.15 | NA | NA | NA | NA | NA | NA | NA | NA | NA | NA | NA | NA |
|  | CA0.20 | CA0.20 | NA | NA | NA | NA | NA | NA | NA | NA | NA | NA | NA | NA |
| 93. Woloszczuk(1998) [S93] | Roc0.80 | Roc0.90 | NA | NA | NA | NA | NA | NA | NA | NA | NA | NA | NA | NA |
|  | Roc0.60 | Roc0.60 | NA | NA | NA | NA | NA | NA | NA | NA | NA | NA | NA | NA |
| 94. Wu (2023)† [S94] | NO | PL | NA | NA | NA | NA | NA | NA | NA | NA | NA | NA | NA | NA |
|  | Roc0.45 | Roc0.45 | NA | NA | NA | NA | NA | NA | NA | NA | NA | NA | NA | NA |
|  | Roc0.60 | Roc0.60 | NA | NA | NA | NA | NA | NA | NA | NA | NA | NA | NA | NA |
| 95a. Zeng (2017) [S95a] | Miv0.15 | Miv0.15 | NA | NA | NA | NA | NA | NA | NA | NA | NA | NA | NA | NA |
|  | Miv0.20 | Miv0.20 | NA | NA | NA | NA | NA | NA | NA | NA | NA | NA | NA | NA |
| 95b. Zeng (2017) [S95b] | Miv0.15 | Miv0.15 | NA | NA | NA | NA | NA | NA | NA | NA | NA | NA | NA | NA |
|  | Miv0.20 | Miv0.20 | NA | NA | NA | NA | NA | NA | NA | NA | NA | NA | NA | NA |
| 95c. Zeng (2017) [S95c] | Miv0.20 | Miv0.20 | NA | NA | NA | NA | NA | NA | NA | NA | NA | NA | NA | NA |
|  | Miv0.25 | Miv0.25 | NA | NA | NA | NA | NA | NA | NA | NA | NA | NA | NA | NA |
| 95d. Zeng (2017) [S95d] | Miv0.20 | Miv0.20 | NA | NA | NA | NA | NA | NA | NA | NA | NA | NA | NA | NA |
|  | Miv0.25 | Miv0.25 | NA | NA | NA | NA | NA | NA | NA | NA | NA | NA | NA | NA |
| 95e. Zeng (2017) [S95e] | Miv0.20 | Miv0.20 | NA | NA | NA | NA | NA | NA | NA | NA | NA | NA | NA | NA |
|  | Miv0.25 | Miv0.25 | NA | NA | NA | NA | NA | NA | NA | NA | NA | NA | NA | NA |
| 95f. Zeng (2017) [S95f] | Miv0.15 | Miv0.15 | NA | NA | NA | NA | NA | NA | NA | NA | NA | NA | NA | NA |
|  | Miv0.20 | Miv0.20 | NA | NA | NA | NA | NA | NA | NA | NA | NA | NA | NA | NA |
| 95g. Zeng (2017) [S95g] | Miv0.15 | Miv0.15 | NA | NA | NA | NA | NA | NA | NA | NA | NA | NA | NA | NA |
|  | Miv0.20 | Miv0.20 | NA | NA | NA | NA | NA | NA | NA | NA | NA | NA | NA | NA |
| 95h. Zeng (2017) [S95h] | Miv0.20 | Miv0.20 | NA | NA | NA | NA | NA | NA | NA | NA | NA | NA | NA | NA |
|  | Miv0.25 | Miv0.25 | NA | NA | NA | NA | NA | NA | NA | NA | NA | NA | NA | NA |
|  |  |  |  |  |  |  |  |  |  |  |  |  |  | ^^[[1]](#footnote-1)^^ |

**Table S2.** Summary providing an overview of haemodynamic characteristics, specifically blood pressure and heart rate, measured across 95 studies, encompassing 280 study arms and 7263 paediatric participants. Evaluations were conducted immediately before intubation (0 minutes) and at one (1) and three (3) minutes post-laryngoscopy and intubation. The haemodynamic parameters assessed include systolic (SAP), mean (MAP), and diastolic (DAP) blood pressures, along with heart rate (HR), recorded at the specified time points.Treatment groups received either NMBA (specified by agent and dose in mg/kg) or a placebo, classified as relaxant-free (NO), either as placebo without (PL) or with opioids (PL-Op).The NMBA agents analyzed include atracurium (Atr), cis-atracurium (CA), mivacurium (Miv), rocuronium (Roc), suxamethonium (Sux), and vecuronium (Vec). NA: not available, denotes missing values.

**Supplementary material File 3c: References of included articles.**

[S1] An TH. The onset of neuromuscular blockade and hemodynamic effects after atracurium, vecuronium, or rocuronium in children. Korean J Anesthesiol. 2003; 44: 163-168. DOI: 10.4097/kjae.2003.44.2.163

[S2] Annila P, Viitanen H, Reinikainen P, Baer G, Lindgren L. Induction characteristics of thiopentone/suxamethonium, propofol/alfentanil or halothane alone in children aged 1-3 years. Eur J Anaesthesiol. 1999 Jun;16(6):359-66. doi: 10.1046/j.1365-2346.1999.00484.x.

[S3] Apilioğulları S, Ökesli S, Reisli R, Duman A, Öğün CO. Effects of nondepolarizing muscle relaxants mivacurium and rocuronium on neuromuscular blockade and intubation conditions in children.

Genel Tıp Dergisi 2006; 16: 153-9.

[S4] Bala M, Ahlawat G, Sachdeva A, Ahlawat MS, Kshetrapal K, Bala R. Comparative Study of the Use of Cisatracurium and Atracurium in Children under General Anaesthesia.

Annals of Health Research. 2024; 10: 351-60. DOI: https://doi.org/10.30442/ahr.1004-04-255

[S5] Balakrishnan K, Mrunalini AK, Mathan Kumar R, Krishnan N, Ravi S. Comparison of mivacurium and atracurium for intubation in paediatric patients. Ind J Anaesth. 1999; 43: 61-62

[S6] Bansal S, Pandit Rao MM, Pandit Rao MM. Intubating Conditions and Efficacy of Rocuronium versus Atracurium in Paediatric Patients undergoing Elective Surgeries under General Anaesthesia: A Randomised Clinical Trial. Journal of Clinical and Diagnostic Research (JCDR). 2023; 17(1). DOI: 10.7860/JCDR/2023/61085.17427

[S7] Bartolek D, Jakobović J, Bartolek F, Finci D, Munjiza A. Reduced-dose rocuronium for day-case tonsillectomy in children where volatile anaesthetics are not used: operating room time saving. Paediatr Anaesth. 2010 Jan;20(1):47-55. doi: 10.1111/j.1460-9592.2009.03175.x.

[S8] Barve M, Sharma R. Comparison of intubating conditions and time course of action of rocuronium bromide and succinylcholine in paediatric patients. Indian J Anaesth. 2002;46:465–8.

[S9] Bhattacharya P, Ahmad S, Behary BK, Kushwaha BB, Ranjan P, Sharma P. Comparison of Intubating Condition and Cardiovascular Effects after administration of Rocuronium and Vecuronium in Children. J. Anaesthesiol Clin Pharmacol. 2008; 24(4): 458-62.

[S10] Blair JM, Hill DA, Bali IM, Fee JP. Tracheal intubating conditions after induction with sevoflurane 8% in children. A comparison with two intravenous techniques. Anaesthesia. 2000 Aug;55(8):774-8. doi: 10.1046/j.1365-2044.2000.01470.x.

[S11] Blair JM, Hill DA, Wilson CM, Fee JP. Assessment of tracheal intubation in children after induction with propofol and different doses of remifentanil. Anaesthesia. 2004 Jan;59(1):27-33. doi: 10.1111/j.1365-2044.2004.03524.x..

[S12] Bock M, Haselmann L, Böttiger BW, Motsch J. Priming with rocuronium accelerates neuromuscular block in children: a prospective randomized study. Can J Anaesth. 2007 Jul;54(7):538-43. doi: 10.1007/BF03022317. Erratum in: Can J Anaesth. 2007 Nov;54(11):961.

[S13] Bucx MJ, Van Geel RT, Meursing AE, Stijnen T, Scheck PA. Forces applied during laryngoscopy in children. Are volatile anaesthetics essential for suxamethonium induced muscle rigidity? Acta Anaesthesiol Scand. 1994 Jul;38(5):448-52. doi: 10.1111/j.1399-6576.1994.tb03927.x.

[S14] Castillo-Peralta LA, Mandujano-Martínez AM, Castillo-Zamora C, Gutiérrez-Castrellón P. [Effective doses of rocuronium for successful intubation in children under 2 years of age undergoing surgery.] Rev. mex. anestesiol. 2004; 27: 196-199

[S15] Chee HL, Pua HL, Tan S. The efficacy and safety of mivacurium in children in Singapore. Singapore Med J. 1998 May;39(5):200-1.

[S16] Cheng KI, Chu KS, Chen WC, Tang CS. The train of four ratio decreases to zero in anesthetized children is the guide to achieve a satisfactory intubation condition. Kaohsiung J Med Sci. 2002 Jan;18(1):23-9.

[S17] Cheng CA, Aun CS, Gin T. Comparison of rocuronium and suxamethonium for rapid tracheal intubation in children. Paediatr Anaesth. 2002 Feb;12(2):140-5. doi: 10.1046/j.1460-9592.2002.00771.x.

[S18] Cook DR, Gronert BJ, Woelfel SK. Comparison of the neuromuscular effects of mivacurium and suxamethonium in infants and children. Acta Anaesthesiol Scand Suppl. 1995;106:35-40. doi: 10.1111/j.1399-6576.1995.tb04307.x.

[S19] Cook-Sather SD, Tulloch HV, Cnaan A, Nicolson SC, Cubina ML, Gallagher PR, Schreiner MS. A comparison of awake versus paralyzed tracheal intubation for infants with pyloric stenosis. Anesth Analg. 1998 May;86(5):945-51. doi: 10.1097/00000539-199805000-00006.

[S20] Crawford MW, Hayes J, Tan JM. Dose-response of remifentanil for tracheal intubation in infants. Anesth Analg. 2005 Jun;100(6):1599-1604. doi: 10.1213/01.ANE.0000150940.57369.B5.

[S21] Deepak R, Seema S, Namrata J, Sanyukta P. comparative study to evaluate the efficacy of cisatracurium and rocuronium for endotracheal intubation in pediatric patients: A prospective randomized study. Asian Journal of Medical Sciences. 2024: 15(9):35-40. doi: 10.71152/ajms.v15i9.4108

[S22] Desai D, Skapdi M. Comparative study of 3*ed95 rocuronium versus succinylcholine. A Intubating agent in a pediatric patient.

Indian Journal of Applied Basic Medical Sciences (IJABMS). 2019;21A: 90-5.

[S23] Devys JM, Mourissoux G, Donnette FX, Plat R, Schauvliège F, Le Bigot P, Dureau P, Plaud B. Intubating conditions and adverse events during sevoflurane induction in infants. Br J Anaesth. 2011 Feb;106(2):225-9. doi: 10.1093/bja/aeq346.

[S24] Eikermann M, Renzing-Köhler K, Peters J. Probability of acceptable intubation conditions with low dose rocuronium during light sevoflurane anaesthesia in children. Acta Anaesthesiol Scand. 2001 Sep;45(8):1036-41. doi: 10.1034/j.1399-6576.2001.450819.x.

[S25] Eikermann M, Hunkemöller I, Peine L, Armbruster W, Stegen B, Hüsing J, Peters J. Optimal rocuronium dose for intubation during inhalation induction with sevoflurane in children. Br J Anaesth. 2002 Aug;89(2):277-81. doi: 10.1093/bja/aef177.

[S26] Fang Q，Feng X，Huang Y，Zhu Z，Tao Y. [Appraisal of anesthesia effect of mivacurium and CIS atracurium besilate in pediatric anesthesia in tonsillectomy and adenoidectomy.]

J Mod Med Health. 2015: 31(5): 660-2. doi:10.3969/j.issn.1009-5519.2015.05.008

[S27] Fletcher JE, Heard CM. The clinical effect of mixing different proportions of rocuronium and mivacurium. Paediatr Anaesth. 2004 Feb;14(2):152-7. doi: 10.1111/j.1460-9592.2004.01166.x.

[S28] Frediani M, Capanna M, Casini L, Lorenzetti MG, Bianchini G, Pacini P. Uso dei miorilassanti a media durata d'azione a dosaggi ridotti in interventi di adenotonsillectomia [The use of low doses of intermediate acting muscle relaxants in adenotonsillectomy]. Minerva Anestesiol. 1993 Mar;59(3):109-14.

[S29] Friesdorf W, Schultz M, Fösel T, Altemeyer KH. Pharmakodynamik von Vecuronium im Kleinkindesalter bei intravenöser Narkoseeinleitung mit Ketamin [Pharmacodynamics of vecuronium in infants during intravenous induction of anesthesia with ketamine]. Anaesthesist. 1986 Feb;35(2):99-102.

[S30] Fuchs-Buder T, Tassonyi E. Intubating conditions and time course of rocuronium-induced neuromuscular block in children. Br J Anaesth. 1996 Sep;77(3):335-8. doi: 10.1093/bja/77.3.335.

[S31] Gelberg J, Kongstad L, Werner O. Intubation conditions in young infants after propofol and remifentanil induction with and without low-dose rocuronium. Acta Anaesthesiol Scand. 2014 Aug;58(7):820-5. doi: 10.1111/aas.12346.

[S32] Gera S, Dali JS, Sharma KR, Garg R, Arya M. Evaluation of intubating conditions in children after sevoflurane induction using propofol or rocuronium bromide--a randomised, prospective, double blind study. Acta Anaesthesiol Belg. 2015;66(4):25-30.

[S33] Gnani BCN, Uma BR. A clinical comparative study of succinylcholine versus rocuronium in various doses for pediatric intubation. Indian Journal of Clinical Anaesthesia 2017; 4: 214-8.

DOI: 10.18231/2394-4994.2017.0045

[S34] González del Pino I, Domech A, Rodríguez Fundora M, Reboredo Rodríguez Y, Suárez

Rodríguez LA, Liriano Rodríguez MI. Bromuro de veruconio o besilato de atracurio para la intubación endotraqueal en el paciente pediátrico. [Vecuronium bromide or atracuric bensilate for endotracheal intubation in the pediatric patient]. Revista Médica Electrónica. 2008;30 (1) Disponible en URL:http://www.cpimtz.sld.cu/revista%20medica/ano%202008/vol1%202008/ tema 1.htm.

[S35] Green DW, Fisher M, Sockalingham I. Mivacurium compared with succinylcholine in children with liver disease. Br J Anaesth. 1998 Sep;81(3):463-5. doi: 10.1093/bja/81.3.463.

[S36] Grubhofer G, Petcold R, Donner A, Klimscha W, Steuer J, Semsroth M. Facilitated tracheal intubation in infants: is propofol a safe alternative to atracurium? Paediatr Anaesth. 1993 May;3:139-145. doi.org/10.1111/j.1460-9592.1993.tb00052.x

[S37] Grundmann U, Ismaily AJ, Kleinschmidt S, Motsch J. Vergleichende Untersuchungen von Atracurium und Vecuronium für mittellang dauernde operative Eingriffe bei Säuglingen und Kleinkindern [Comparative studies of atracurium and vecuronium for medium-length surgical procedures in infants and young children]. Anästhesiol Intensivmed Notfallmed Schmerzther. 1991 Feb;26(1):25-8. German. doi: 10.1055/s-2007-1000532.

[S38] Hansen D, Heitz E, Toussaint S, Schaffartzik W, Striebel HW. Deep halothane anaesthesia compared with halothane-suxamethonium anaesthesia for tracheal intubation in young children. Eur J Anaesthesiol. 1997 Jan;14(1):29-34. doi: 10.1046/j.1365-2346.1997.00070.x.

[S39] Huang AP, Kong GY, Liu JS, Jiang JY, Zhang XY. Pharmacodynamics of Cisastracurium in Children at Different Age. China Pharm. 2007;18:2281–3.

[S40] Huh H, Park JJ, Kim JY, Kim TH, Yoon SZ, Shin HW, Lee HW, Lim HJ, Cho JE. Optimal dose of rocuronium bromide undergoing adenotonsillectomy under 5% sevoflurane with fentanyl. Int J Pediatr Otorhinolaryngol. 2017 Oct;101:70-74. doi: 10.1016/j.ijporl.2017.07.030.

[S41] Kapdi MS, Shah B, Nayi M, Kalsaria P, Patel K. Comparative study of rocuronium, vecuronium & atracurium as non depolarising muscle relaxant in paediatric patients.

International Journal of Contemporary Medical Research (IJCMR). 2020; 12: L17-L19. Doi: http://dx.doi.org/10.21276/ijcmr.2020.7.12.23

[S42] Karadeniz Ü, Ünlü S, Çaǧlar S, M. Tokmakoǧlu M. Comparison of intubating conditions and haemodynamic effects of cisatracuriam and atracurium in children.

Türk Anesteziyoloji ve Reanimasyon 2000; 28:512-5.

[S43] Klemola UM, Hiller A. Tracheal intubation after induction of anesthesia in children with propofol--remifentanil or propofol-rocuronium. Can J Anaesth. 2000 Sep;47(9):854-9. doi: 10.1007/BF03019664.

[S44] Kulkarni KR, Patil MR, Shirke AM, Jadhav SB. Perioperative respiratory complications in cleft lip and palate repairs: An audit of 1000 cases under 'Smile Train Project'. Indian J Anaesth. 2013 Nov;57(6):562-8. doi: 10.4103/0019-5049.123328.

[S45] Kumar A, Kumar A, Bharti AK, Choudhary A, Hussain M, Dhiraj S. A Randomized Double-Blind Comparative Study of the Intubating Conditions and Hemodynamic Effects of Rocuronium and Succinylcholine in Pediatric Patients. Cureus. 2023 Sep 4;15(9):e44631. doi: 10.7759/cureus.44631.

[S46] Lekmanov AU, Suvorov SG, Tartakovskiĭ I. Intubatsiia trakhei u deteĭ pri primenenii nedepoliarizuiushchikh relaksantov benzilizokhinolinovogo riada atrakuriuma i mivakuriuma [Tracheal intubation in children using the benzyl isoquinoline-series nondepolarizing relaxants atracurium and mivacurium]. Anesteziol Reanimatol. 1998 Jan-Feb;(1):24-7. Russian..

[S47] Lysakowski C, Fuchs-Buder T, Tassonyi E. Mivacurium or vecuronium for paediatric ENT surgery. Clinical experience and cost analysis. Anaesthesist. 2000 May;49(5):387-91. doi: 10.1007/s001010070106.

[S48] Malhotra P, Saxena N, Kiran U, Choudhary M. Comparison of rocuronium and vecuronium in paediatric cardiac surgery, using sevoflurane anaesthesia.

Indian J Thorac Cardiovasc Surg 2002; 18:105-9. https://doi.org/10.1007/s12055-002-0016-6.

[S49] Mangat PS, Evans DE, Harmer M, Lunn JN. A comparison between mivacurium and suxamethonium in children. Anaesthesia. 1993 Oct;48(10):866-9.

[S50] Mazurek AJ, Rae B, Hann S, Kim JI, Castro B, Coté CJ. Rocuronium versus succinylcholine: are they equally effective during rapid-sequence induction of anesthesia? Anesth Analg. 1998 Dec;87(6):1259-62. doi: 10.1097/00000539-199812000-00009.

[S51] Mikailu A, Atiku M, Abdurrahman A, Salahu D, Adesope S. A Comparative Study between Propofol-fentanyl versus Propofol-suxamethonium for Ease of Endotracheal Intubation in Children. J West Afr Coll Surg. 2023 Jan-Mar;13(1):55-59. doi: 10.4103/jwas.jwas_229_22.

[S52] Módolo NS, do Nascimento Júnior P, Croitor LB, Vianna PT, Castiglia YM, Ganem EM, Braz JR, Takito DS, Takaesu LA. Onset time and duration of rocuronium, atracurium and mivacurium in pediatric patients. Rev Bras Anestesiol. 2002 Apr;52(2):185-96. English, Portuguese. doi: 10.1590/s0034-70942002000200006.

[S53] Montgomery CJ, Steward DJ. A comparative evaluation of intubating doses of atracurium, d-tubocurarine, pancuronium and vecuronium in children. Can J Anaesth. 1988 Jan;35(1):36-40. doi: 10.1007/BF03010542.

[S54] Morgan JM, Barker I, Peacock JE, Eissa A. A comparison of intubating conditions in children following induction of anaesthesia with propofol and suxamethonium or propofol and remifentanil. Anaesthesia. 2007 Feb;62(2):135-9. doi: 10.1111/j.1365-2044.2006.04903.x.

[S55] Mortazavi MT, Parish M, Abedini N, Baradaran R, Abafattash G, Ansari M. Tracheal intubation in children after induction of anesthesia with propofol and remifentanil without a muscle relaxant.

Rawal Medical Journal (RMJ) 2010; 35: 6-9

[S56] Nadirsha A, Agrawal N, Karim HMR. Atracurium Versus Cis-Atracurium for Laryngeal Relaxation and Hemodynamic Stability in Pediatric Patients: A Randomized, Double-Blind Study. Cureus. 2023 Jun 24;15(6):e40882. doi: 10.7759/cureus.40882.

[S57] Naguib M, Samarkandi AH, Ammar A, Turkistani A. Comparison of suxamethonium and different combinations of rocuronium and mivacurium for rapid tracheal intubation in children. Br J Anaesth. 1997 Oct;79(4):450-5. doi: 10.1093/bja/79.4.450.

[S58] Nava-Ocampo AA, Aguirre-Garay FT, Velázquez-Armenta EY, Moyao-García D. Effect of mivacurium 200 and 250 &mgr;g/kg in infants during isoflurane anesthesia: a randomized controlled trial [ISRCTN07742712]. BMC Anesthesiol. 2001;1(1):1. doi: 10.1186/1471-2253-1-1.

[S59] Naziri F, Amiri HA, Rabiee M, Banihashem N, Nejad FM, Shirkhani Z, Solimanian S. Endotracheal intubation without muscle relaxants in children using remifentanil and propofol: Comparative study. Saudi J Anaesth. 2015 Oct-Dec;9(4):409-12. doi: 10.4103/1658-354X.159465.

[S60] NG KP, Wang CY. Alfentanil for intubation under halothane anaesthesia in children. Paediatr Anaesth. 1999;9(6):491-4. doi: 10.1046/j.1460-9592.1999.00420.x.

[S61] Oztekin S, Hepaguşlar H, Kilercik H, Kar AA, Boyaci F, Elar Z. Low doses of rocuronium during remifentanil-propofol-based anesthesia in children: comparison of intubating conditions. Paediatr Anaesth. 2004 Aug;14(8):636-41. doi: 10.1111/j.1460-9592.2004.01273.x.

[S62] Papagiannopoulou P, Sfyra E, Georgiou M, Georgiadou T, Kanakoudis F. Onset of action and intubating conditions after administration of rocuronium or mivacurium in children. Greek E-Journal of Perioperative Medicine 2008; 6: 79-85.

[S63] Park S, Kim JH, Bae JC, Lee JR, Kim MS. Tracheal intubation with or without a neuromuscular blocking agent for a short surgical procedure in children: Prospective, randomized, double-blind trial. Paediatr Anaesth. 2021 Aug;31(8):863-870. doi: 10.1111/pan.14205.

[S64] Pineda Díaz MV, González Guzmán M, López Flores M. Neuromuscular blockade induced by rocuronium compared with vecuronium and atracurium during intravenous anesthesia in children. Rev. Mex. Anestesiol. 1996; 19: 102-7.

[S65] Politis GD, Brill J, Jones J. Use of low-dose rocuronium for intubation of children during volunteer surgery abroad. Paediatr Anaesth. 2005 Aug;15(8):648-52. doi: 10.1111/j.1460-9592.2004.01519.x.

[S66] Rapp HJ, Altenmueller CA, Waschke C. Neuromuscular recovery following rocuronium bromide single dose in infants. Paediatr Anaesth. 2004 Apr;14(4):329-35. doi: 10.1046/j.1460-9592.2003.01216.x.

[S67] Rizvanović N, Čaušević S, Šabanović A. Conditions of endotracheal intubation with and without muscle relaxant in children. Med Glas (Zenica). 2017 Feb 1;14(1):41-48. doi: 10.17392/865-16.

[S68] Rodney GE, Reichert CC, O'Regan DN, Blackstock D, Steward DJ. Propofol or propofol/alfentanil compared to thiopentone/succinylcholine for intubation of healthy children.

Can J Anaesth. 1992; 39: A129.

[S69] Said-Ahmed HAEF. Intubation condition after low dose rocuronium with propofol versus sevoflurane in children. Acta Anaesth Italica. 2006; 57(1):39-50

[S70] Salawu MM, Ogboli-Nwasor EO, Jamgbadi SS, Akpa FN. A study of intubating conditions: Sevoflurane versus propofol-suxamethonium in children. Niger Postgrad Med J. 2017 Jul-Sep;24(3):155-161. doi: 10.4103/npmj.npmj_66_17.

[S71] Scheiber G, Ribeiro FC, Marichal A, Bredendiek M, Renzing K. Intubating conditions and onset of action after rocuronium, vecuronium, and atracurium in young children. Anesth Analg. 1996 Aug;83(2):320-4. doi: 10.1097/00000539-199608000-00020.

[S72] Schultz J, Crawford M. Intubating conditions 30 seconds after rocuronium or succinylcholine in children. Anesth Analg. 1998; 86: 417S. doi: 10.1097/00000539-199802001-0041

[S73] Senel AC, Aktürk G, Yurtseven M. Comparison of intubation conditions under propofol in children--alfentanil vs atracurium. Middle East J Anaesthesiol. 1996 Oct;13(6):605-11.

[S74] Shaginian AK, Mikhel'son VA, Agavelian EG. [Myoplegia with rocuronium bromide in children]. Anesteziol Reanimatol. 2008 Jan-Feb;(1):29-32.

[S75] Shaikh SI, Bellagali VP. Tracheal intubation without neuromuscular block in children. Indian J Anaesth. 2010 Jan;54(1):29-34. doi: 10.4103/0019-5049.60493.

[S76] Shakeet J, Lata S, Jasuja S, Saxena M. A Comparative Study of Duration & Onset of Action and Intubating Conditions Between Rocuronium Bromide and Suxamethonium Chloride in Paediatric Patients at Tertiary Care Centre. International Journal of Health and Clinical Research. 2021; 4(21):272-274.

[S77] ShangGuan W, Lian Q, Li J, Gao F. The neuromuscular blocking effect of different doses of cisatracurium in children. Chin J Anesthesiol. 2007; 27: 54-7.

[S78] Shorten GD, Crawford MW, St Louis P. The neuromuscular effects of mivacurium chloride during propofol anesthesia in children. Anesth Analg. 1996 Jun;82(6):1170-5. doi: 10.1097/00000539-199606000-00012.

[S79] Shrey S, Singam A. A Comparative Study of Atracurium and Cisatracurium in paediatric cleft Lip and Cleft palate surgeries. Research Journal of Pharmacy and Technology (RJPT). 2020; 13(2): 867-70. doi: 10.5958/0974-360X.2020.00164.X

[S80] Sloan MH, Lerman J, Bissonnette B. Pharmacodynamics of high-dose vecuronium in children during balanced anesthesia. Anesthesiology. 1991 Apr;74(4):656-9. doi: 10.1097/00000542-199104000-00006.

[S81] Söğüt N, Memiş D, Alpaydin T, Pamukçu Z. (2000) [Comparison of the effects of rocuronium, mivacurium and cisatracurium on hemodynamic parameters, intubation conditions and neuromuscle junction in paediatric patients.] Türk Anesteziyoloji ve Reanimasyon Dernegi Dergisi 2000; 28, 29–32.

[S82] Srivastava U, Kumar A, Gandhi NK, Saxena S, Agarwal S. Comparison of Propofol and Fentanyl with thiopentone and suxamethanium for tracheal intubation in children.

Indian J Anaesth. 2001;45:263–6

[S83] Steyn MP, Quinn AM, Gillespie JA, Miller DC, Best CJ, Morton NS. Tracheal intubation without neuromuscular block in children. Br J Anaesth. 1994 Apr;72(4):403-6. doi: 10.1093/bja/72.4.403.

[S84] Stoddart PA, Mather SJ. Onset of neuromuscular blockade and intubating conditions one minute after the administration of rocuronium in children. Paediatr Anaesth. 1998;8(1):37-40. doi: 10.1046/j.1460-9592.1998.00719.x.

[S85] Tartari S, Marchi M, Guberti A, Vivarelli R, Tosatti S, Gritti G. Bromuro di vecuronio in anestesia pediatrica [Vecuronium bromide in pediatric anesthesia]. Minerva Anestesiol. 1990 May;56(5):153-9.

[S86] Thwaites AJ, Edmends S, Tomlinson AA, Kendall JB, Smith I. Double-blind comparison of sevofluran vs propofol and succinylcholine for tracheal intubation in children. Br J Anaesth. 1999 Sep;83(3):410-4. doi: 10.1093/bja/83.3.410.

[S87] Ved SA, Chen J, Reed M, Fleming N. Intubation with low-dose atracurium in children. Anesth Analg. 1989 May;68(5):609-13.

[S88] Villegas-Sánchez F, Charles-Torres JD, Moyao-García D, Nava-Ocampo AA, Velázquez-Armenta EY. Rocuronium administration in children during isoflurane anesthesia: neuromuscular effects. Arch Med Res. 1999 Jul-Aug;30(4):307-14. doi: 10.1016/s0188-0128(99)00029-9.

[S89] Voss J, Riedel T, Sommer M, Rosolski T. [Cis-atracurium--an equivalent substitution for atracurium in pediatric anesthesia?]. Anaesthesiol Reanim. 2002;27(4):93-7.

[S90] Wei LX, Deng XM, Liu JH, et al. Inductions and intubating conditions with sevoflurane and different doses of remifentanil without muscle relaxant in children.

Acta Acad. Med. Sin. 2008; 30: 723-7. doi: 10.3881/j.issn. 1000-503X.2008.06.019

[S91] Wei R, Bian Y, Zhang M, Chen Y. Comparison of neuromuscular blocking effects of different doses of cisatracurium in children. Shanghai Med J. 2010; 33: 156-8.

[S92] Wei D, Du J, Fu Y, Jin Q. Application of cisatracurium in rapid tracheal intubation in children. Journal of Shanghai Jiaotong University (Medical Science) 2011; 31: 1625-7. doi: 10.3969/j.issn. 1674-8115.2011.11.026.

[S93] Wu L, Wei S, Xiang Z, Yu E, Chen Z, Qu S, Du Z. Effect of neuromuscular block on surgical conditions during laparoscopic surgery in neonates and small infants: A randomised controlled trial. Eur J Anaesthesiol. 2023 Dec 1;40(12):928-935. doi: 10.1097/EJA.0000000000001898.

[S94] Woloszczuk - Gebicka B. Intubation conditions in children following rocuronium 0.8 mg kg-1. Anesth Analg. 1998; 86: 421S. doi: 10.1097/00000539-199802001-00419

[S95] Zeng R, Liu X, Zhang J, Yin N, Fei J, Zhong S, Hu Z, Hu M, Zhang M, Li B, Li J, Lian Q, ShangGuan W. The efficacy and safety of mivacurium in pediatric patients. BMC Anesthesiol. 2017 Apr 17;17(1):58. doi: 10.1186/s12871-017-0350-2.

[S96] Hung CT, Shih MH, Shih CJ, Liou SC, Kau YC, Chan C, Wong KM. Intubation conditions with low dose rocuronium under sevoflurane induction for children. Chang Gung Med J. 2005 Mar;28(3):174-9.

[S97] Hadimioglu N, Ertugrul F, Ertug Z, Yegin A, Karaguzel G, Erman M. The comparative effect of single dose mivacurium during sevoflurane or propofol anesthesia in children. Paediatr Anaesth. 2005 Oct;15(10):852-7. doi: 10.1111/j.1460-9592.2004.01563.x.

[S98] Kocabaş S, Balcioglu T, Cevik A. [The comparison of the effects of inhalational and intravenous anaesthesia on the neuromuscular block of rocuronium in children].

Türk anesteziyoloji ve reanimasyon 2002; 30: 127‐32

[S99] Kenaan CA, Estacio RL, Bikhazi GB. Pharmacodynamics and intubating conditions of cisatracurium in children during halothane and opioid anesthesia. J Clin Anesth. 2000 May;12(3):173-6. doi: 10.1016/s0952-8180(00)00132-x.

[S100] Meakin GH, Meretoja OA, Perkins RJ, Waite I, Taivainen T, Wirtavuori K, Murphy AK, Raiha L. Tracheal intubating conditions and pharmacodynamics following cisatracurium in infants and children undergoing halothane and thiopental-fentanyl anesthesia. Paediatr Anaesth. 2007 Feb;17(2):113-20. doi: 10.1111/j.1460-9592.2006.02042.x.

[S101] Hopkinson JM, Meakin G, McCluskey A, Baker RD. Dose-response relationship and effective time to satisfactory intubation conditions after rocuronium in children. Anaesthesia. 1997 May;52(5):428-32. doi: 10.1111/j.1365-2044.1997.121-az0115.x.

[S102] Lee SK, Hong JH, Kim AR. Is the rapid sequence induction possible with 0.6 mg/kg rocuronium in pediatric patient? Korean J Anesthesiol. 2010 Jan;58(1):20-4. doi: 10.4097/kjae.2010.58.1.20.

[S103] McCluskey A, Meakin G. Dose-response and minimum time to satisfactory intubation conditions after mivacurium in children. Anaesthesia. 1996 May;51(5):438-41. doi: 10.1111/j.1365-2044.1996.tb07787.x.

[S104] McDonald PF, Sainsbury DA, Laing RJ. Evaluation of the onset time and intubation conditions of rocuronium bromide in children. Anaesth Intensive Care. 1997 Jun;25(3):260-1. doi: 10.1177/0310057X9702500309.

[S105] Simhi E, Brandom BW, Lloyd ME, Gronert BJ, Woelfel SK. Intubation in children after 0.3 mg/kg of mivacurium. J Clin Anesth. 1997 Nov;9(7):576-81. doi: 10.1016/s0952-8180(97)00148-7.

1. [↑](#footnote-ref-1)
