# Supplementary material for: Effect of neuromuscular blocking agents on tracheal intubation quality in paediatric patients: a systematic review using network meta-analysis and meta-regression
Source: Br J Anaesth. 2025 Sep 3;135(6):1787–802. doi: 10.1016/j.bja.2025.08.036 (PMC12799451; doi:10.1016/j.bja.2025.08.036)
Supplement: Multimedia Component 4 [file mmc4.docx]

**Supplementary material File 4.:**

**Timelines of NMBA use in paediatric intubation studies**

**included in the network meta-analysis.**


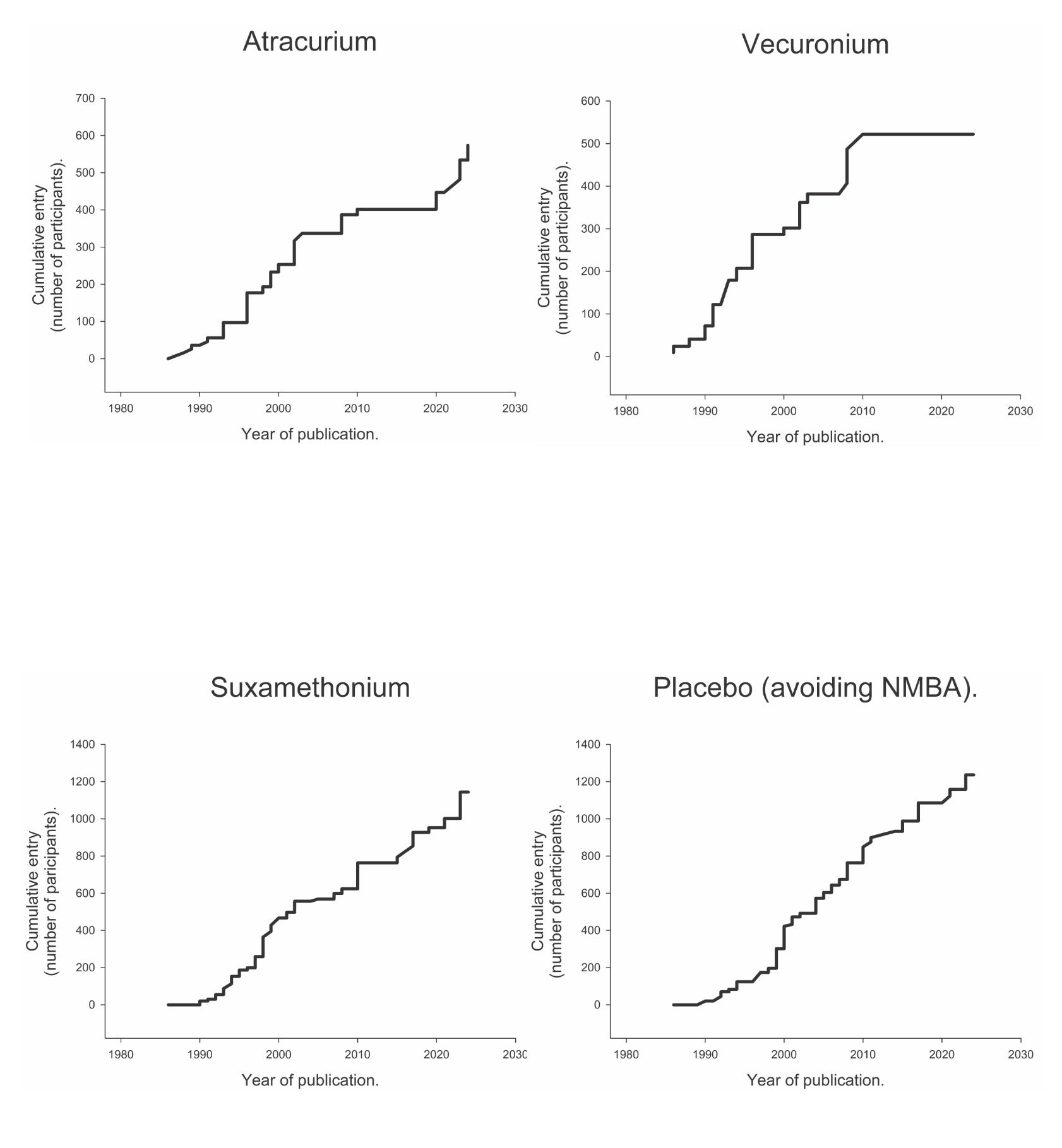


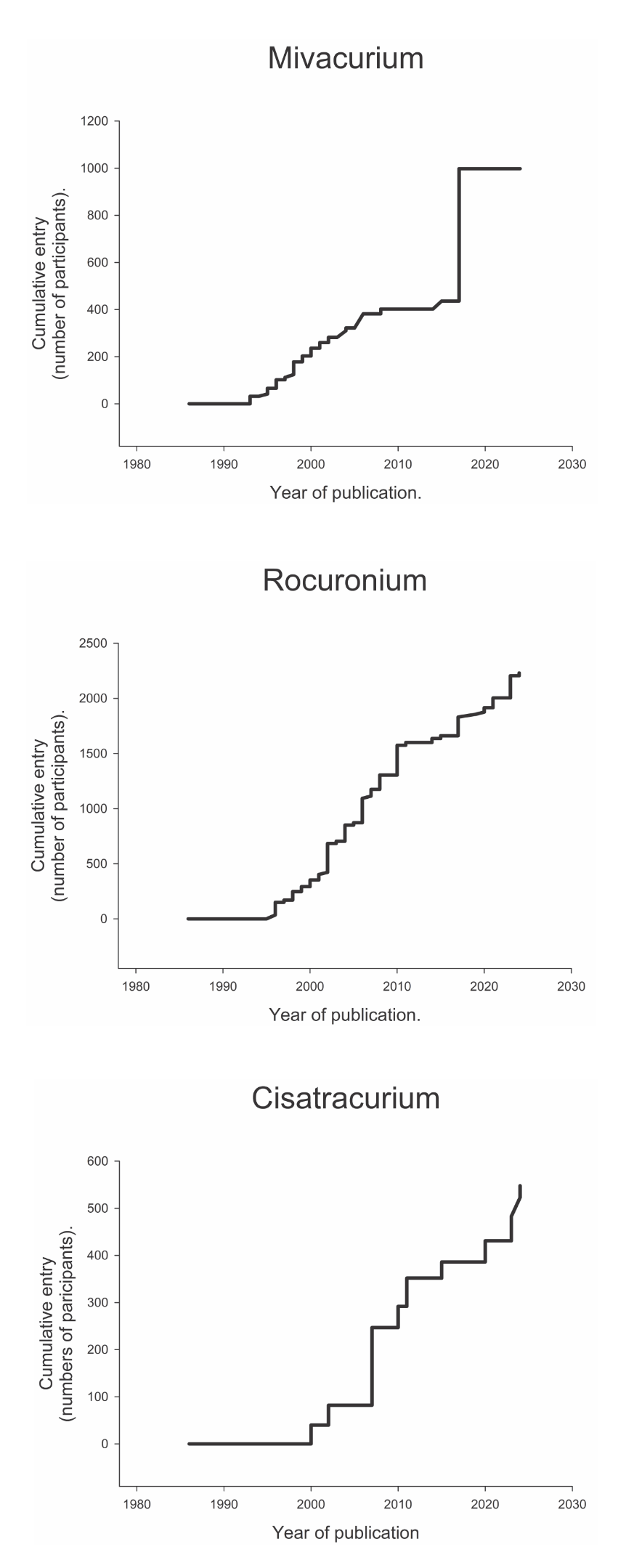


**Figure S1:** Cumulative entry of patients in paediatric intubation studies over time by neuromuscular blocking agent (NMBA) used. Data are shown by publication year, allowing temporal trends in NMBA application to be identified.
