# Supplementary material for: Effect of neuromuscular blocking agents on tracheal intubation quality in paediatric patients: a systematic review using network meta-analysis and meta-regression
Source: Br J Anaesth. 2025 Sep 3;135(6):1787–802. doi: 10.1016/j.bja.2025.08.036 (PMC12799451; doi:10.1016/j.bja.2025.08.036)
Supplement: Multimedia Component 5 [file mmc5.docx]

**Supplementary material File 5:**

**Participant recruitment, and demographics.**

1. **Accrual of participants to included trials according to the year of publication.**


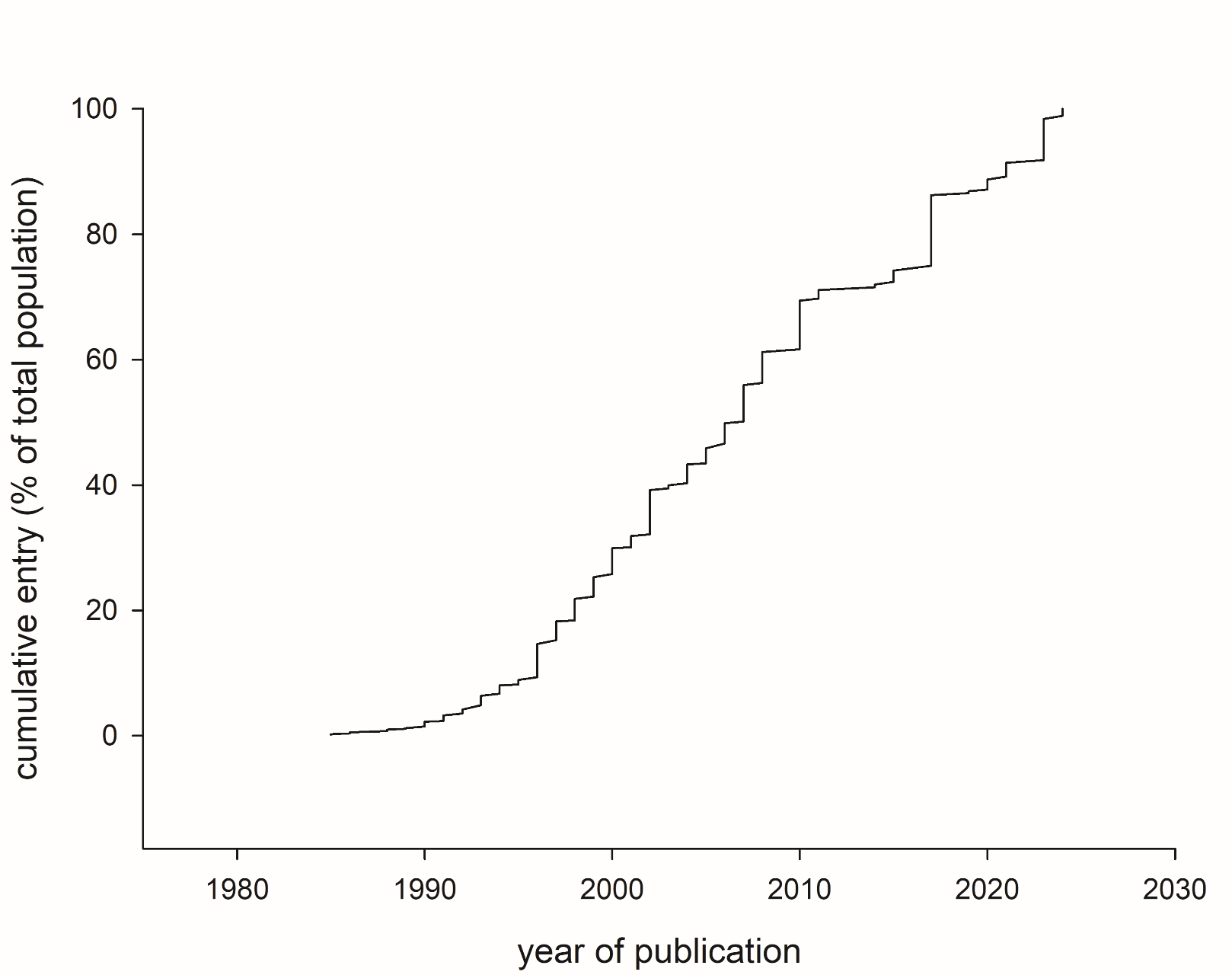


**Figure S2.** Cumulative participant enrollment across the included trials is shown by year of publication. “% of total” indicates the proportion of the 8008 participants from 105 trials in this meta-analysis. Recruitment spanned nearly 39 years (January 1986–November 2024), with 28.1% enrolled in studies published before 2000.

1. **Accrual of participants according to the average age in the treatment arms.**


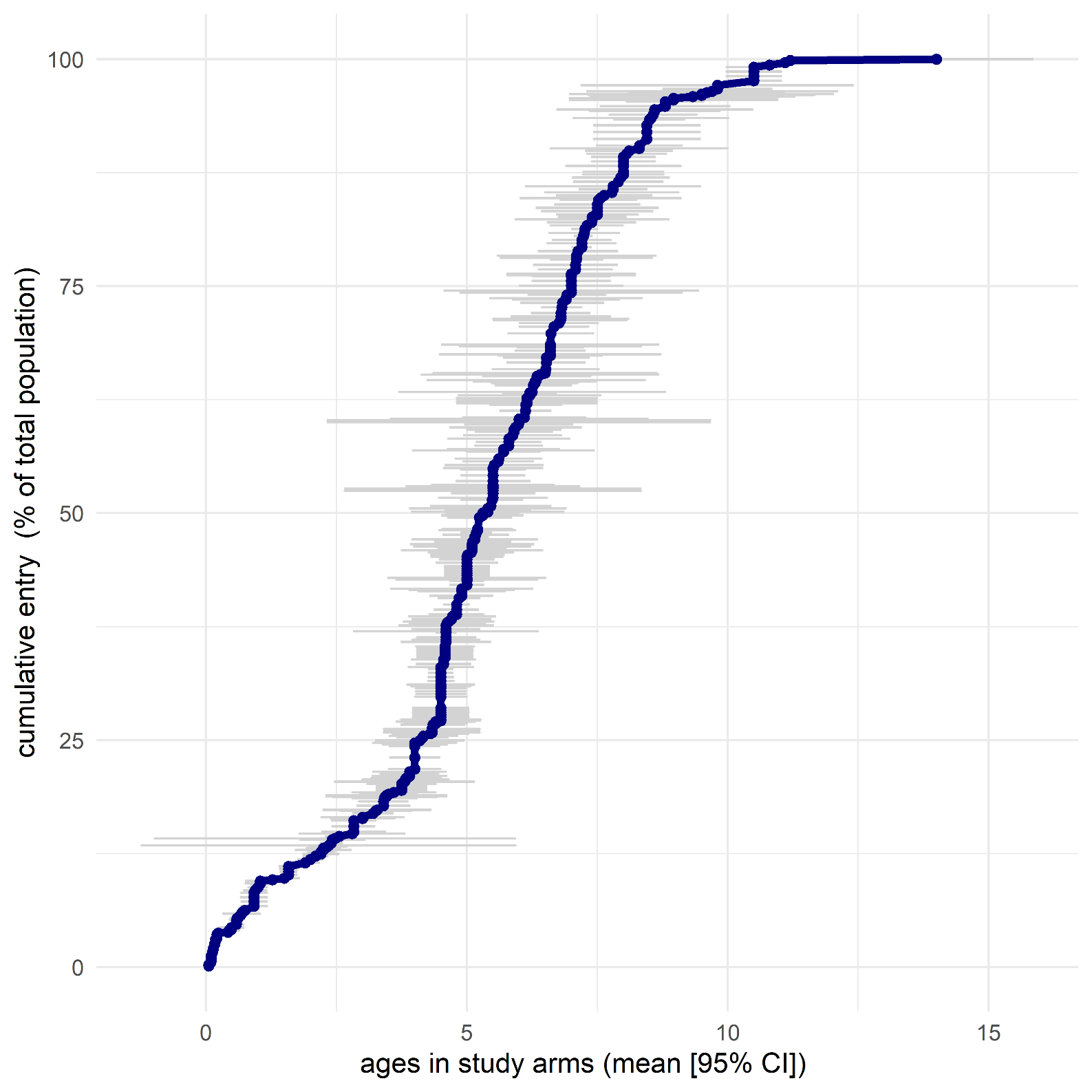


**Figure S3.** Cumulative enrollment of participants (n=8008) across 315 study arms from 105 trials is presented by average age, with gray horizontal lines indicating 95% confidence intervals. The median age was 5.09 [IQR: 3.85–6.80] years, and 95% of participants were younger than 8.80 years. Infants (≤1 year) accounted for 8.50% of the population, while children aged ≤2 years represented 11.58%. The majority of study arms (88.41%) enrolled participants with an average age between 2 and 12 years.

1. **Relationship between paediatric age and weight of the included population.**


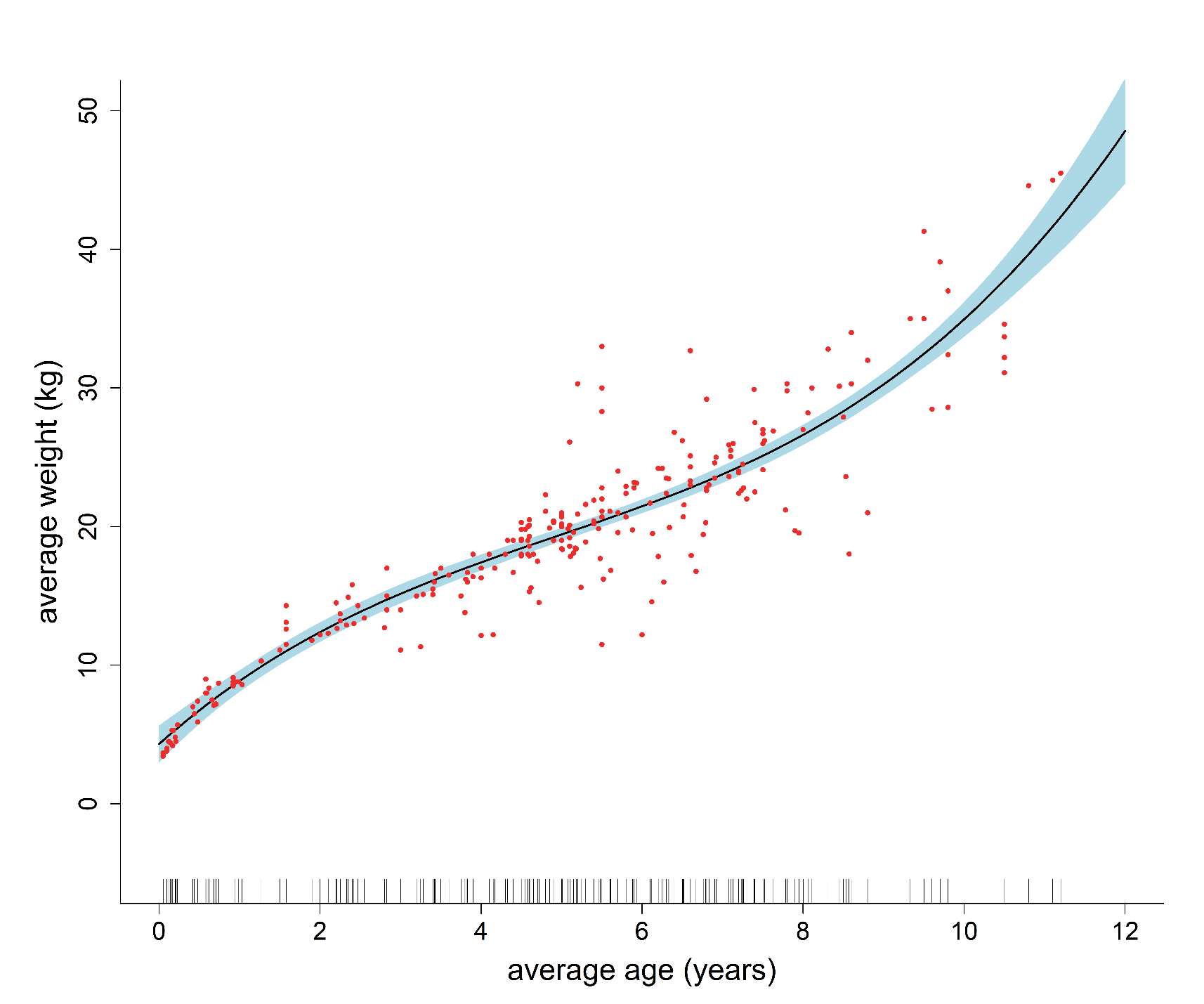


**Figure S4.** The relationship between average age (years) and average weight (kg) across study arms was modeled using a semiparametric regression (generalized additive model [GAM])^[[1]](#footnote-1)^. The blue shaded area shows the 95% confidence interval. The observed pattern closely aligns with established growth standards from the World Health Organization (WHO)^[[2]](#footnote-2)^ and Centers for Disease Control and Prevention (CDC)^[[3]](#footnote-3)^.

1. Harezlak, J., Ruppert, D., & Wand, M. P. (2018). Semiparametric Regression with R. Springer Series in Statistics. Springer. [↑](#footnote-ref-1)
2. https://www.who.int/news/item/27-04-2006-world-health-organization-releases-new-child-growth-standards [↑](#footnote-ref-2)
3. https://www.cdc.gov/growthcharts/who-growth-charts.htm [↑](#footnote-ref-3)
