# Supplementary material for: Effect of neuromuscular blocking agents on tracheal intubation quality in paediatric patients: a systematic review using network meta-analysis and meta-regression
Source: Br J Anaesth. 2025 Sep 3;135(6):1787–802. doi: 10.1016/j.bja.2025.08.036 (PMC12799451; doi:10.1016/j.bja.2025.08.036)
Supplement: Multimedia Component 6 [file mmc6.docx]

**Supplementary material File 6: Risk of Bias assessment^[[1]](#footnote-1),^^[[2]](#footnote-2)^.**

1. **Risk of Bias evaluation for non-randomized controlled trials (ROBINS-I).**
2. Robin-I assessments for individual studies using traffic light plot**.**


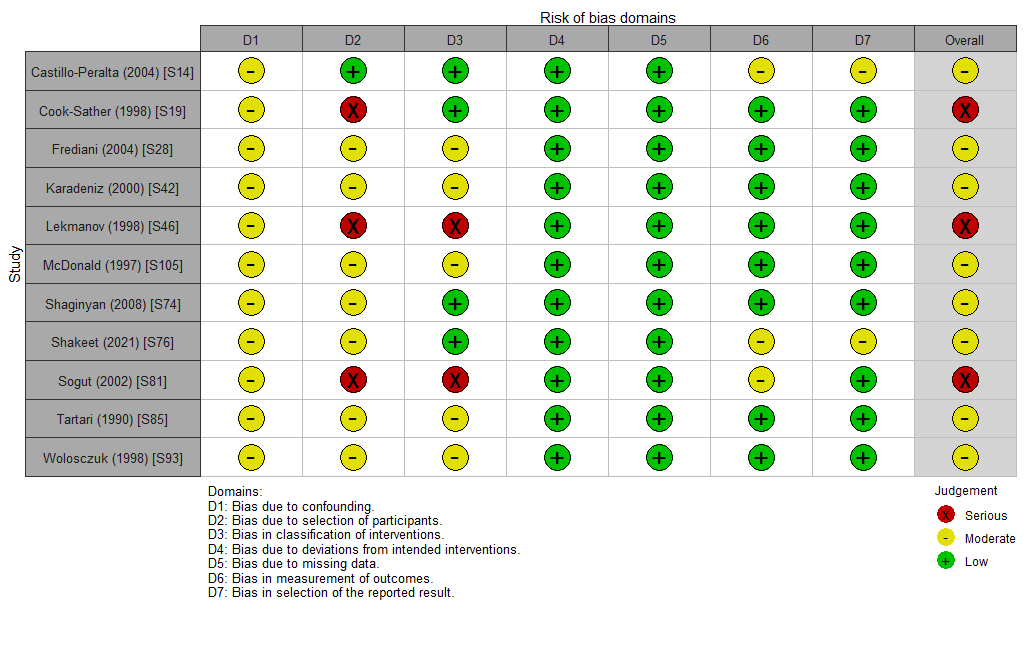


1. Summary bar plot of Robin-I assessment


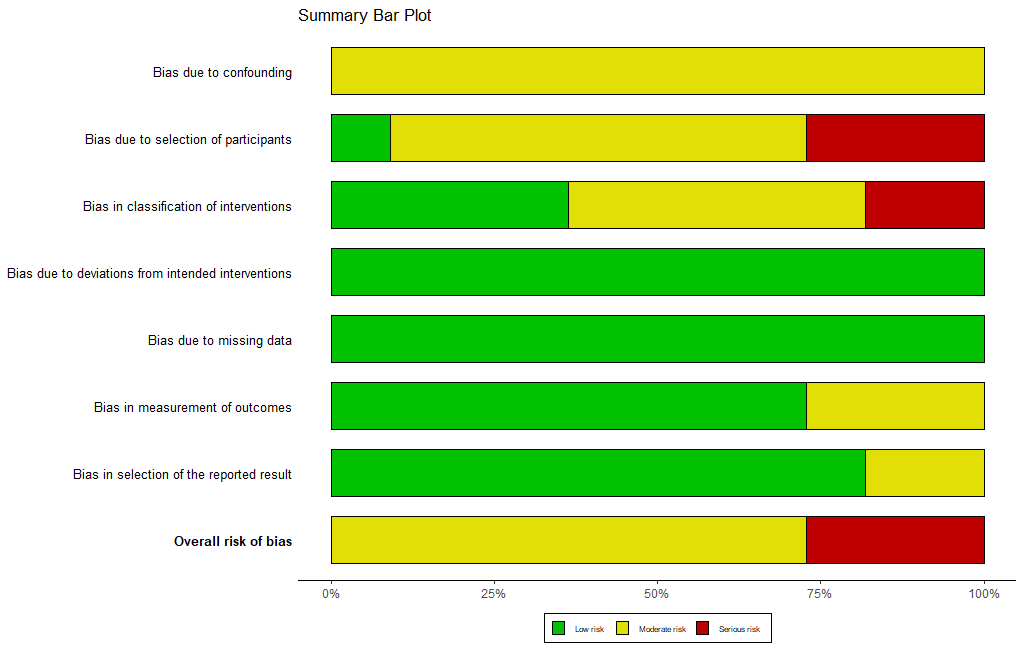


**Figure S5a.** Risk-of-bias assessment using the ROBINS-I tool for non-randomized studies (n=11), presented as a traffic light plot (upper panel) for individual study assessments and a summary bar plot displaying the risk-of-bias judgments across different domains for the included studies^^[[3]](#footnote-3)^^.

1. **Risk of Bias evaluation for randomized controlled trials (Rob2)-**
2. Rob2 assessments of individual studies: traffic light plots.


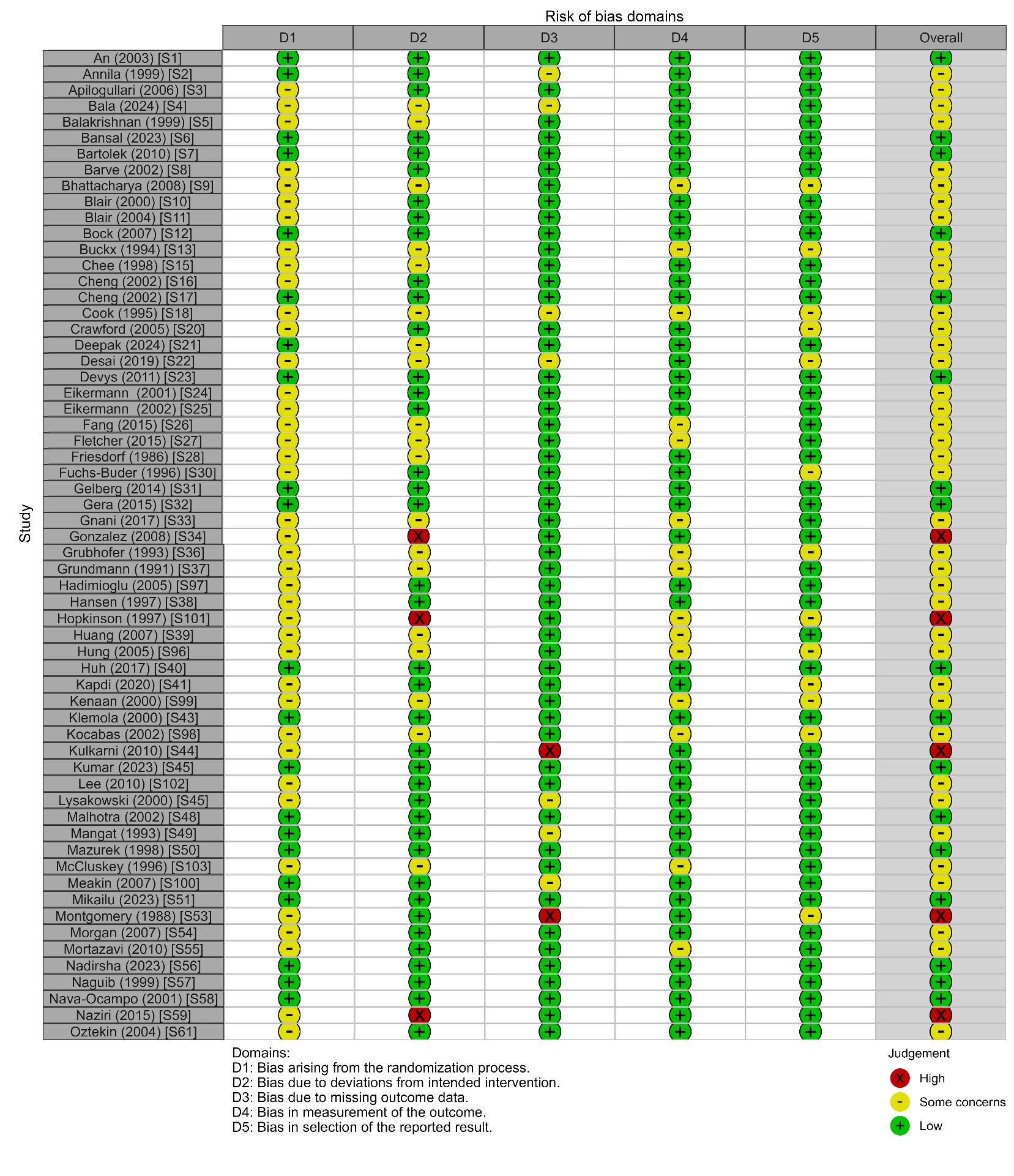


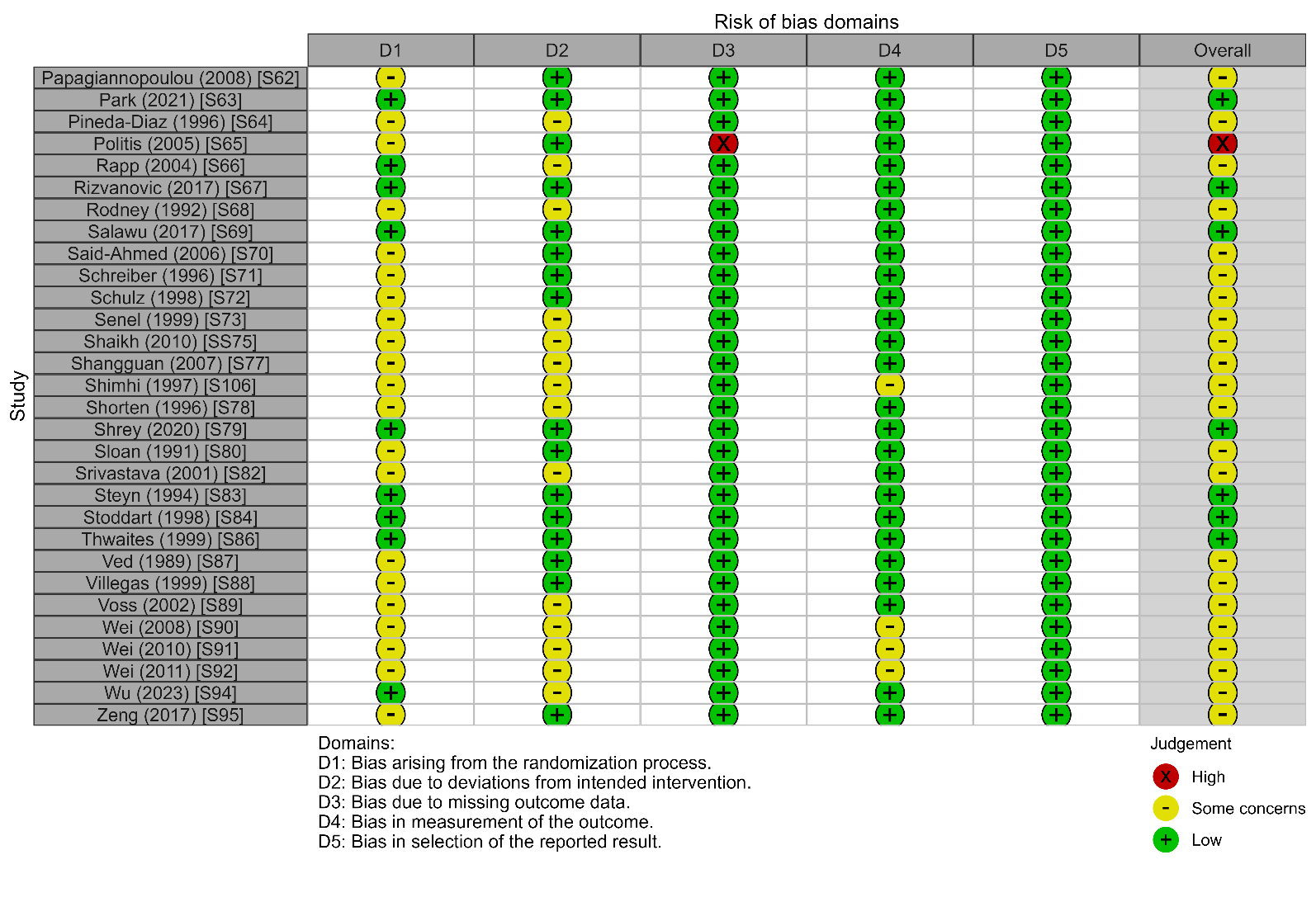


1. Summary bar plot of ROB2 assessment


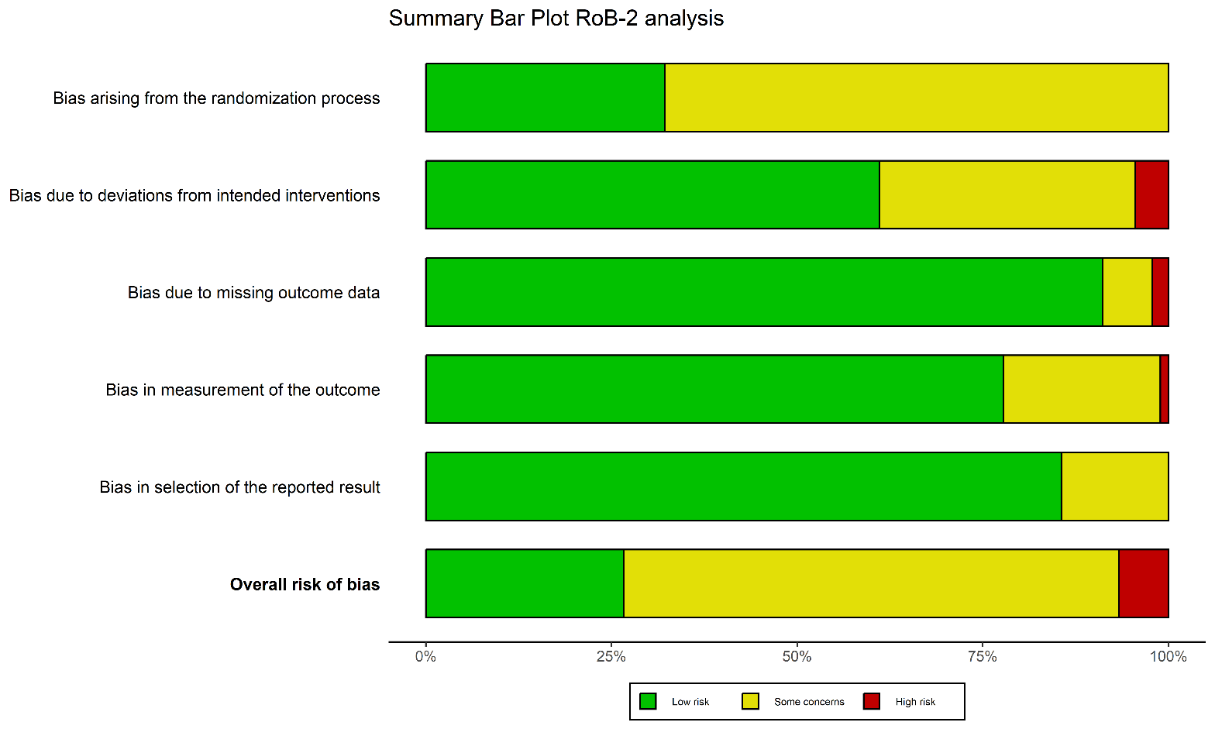


**Figure S5b.** Risk-of-bias assessment using the Rob2 tool for Randomized Controlled trials (n=94), presented as a traffic light plot (upper panel) for individual study assessments and a summary bar plot displaying the risk-of-bias judgments across different domains for the included studies.

1. Sterne JA, Hernán MA, Reeves BC, et al. ROBINS-I: a tool for assessing risk of bias in non-randomised studies of interventions. BMJ. 2016;355:i4919. [↑](#footnote-ref-1)
2. Sterne JAC, Savović J, Page MJ, et al. RoB 2: a revised tool for assessing risk of bias in randomised trials. BMJ. 2019;366:l4898. [↑](#footnote-ref-2)
3. McGuinness LA, Higgins JPT. Risk-of-bias VISualization (robvis): An R package and Shiny web app for visualizing risk-of-bias assessments. Res Synth Methods. 2021 Jan;12(1):55-61. doi: 10.1002/jrsm.1411. [↑](#footnote-ref-3)
