# Supplementary material for: Effect of neuromuscular blocking agents on tracheal intubation quality in paediatric patients: a systematic review using network meta-analysis and meta-regression
Source: Br J Anaesth. 2025 Sep 3;135(6):1787–802. doi: 10.1016/j.bja.2025.08.036 (PMC12799451; doi:10.1016/j.bja.2025.08.036)
Supplement: Multimedia Component 7 [file mmc7.docx]

**Supplementary material File 7.:** **Funnel plots**

1. **Funnelplot network analysis for Excellent intubation Conditions**


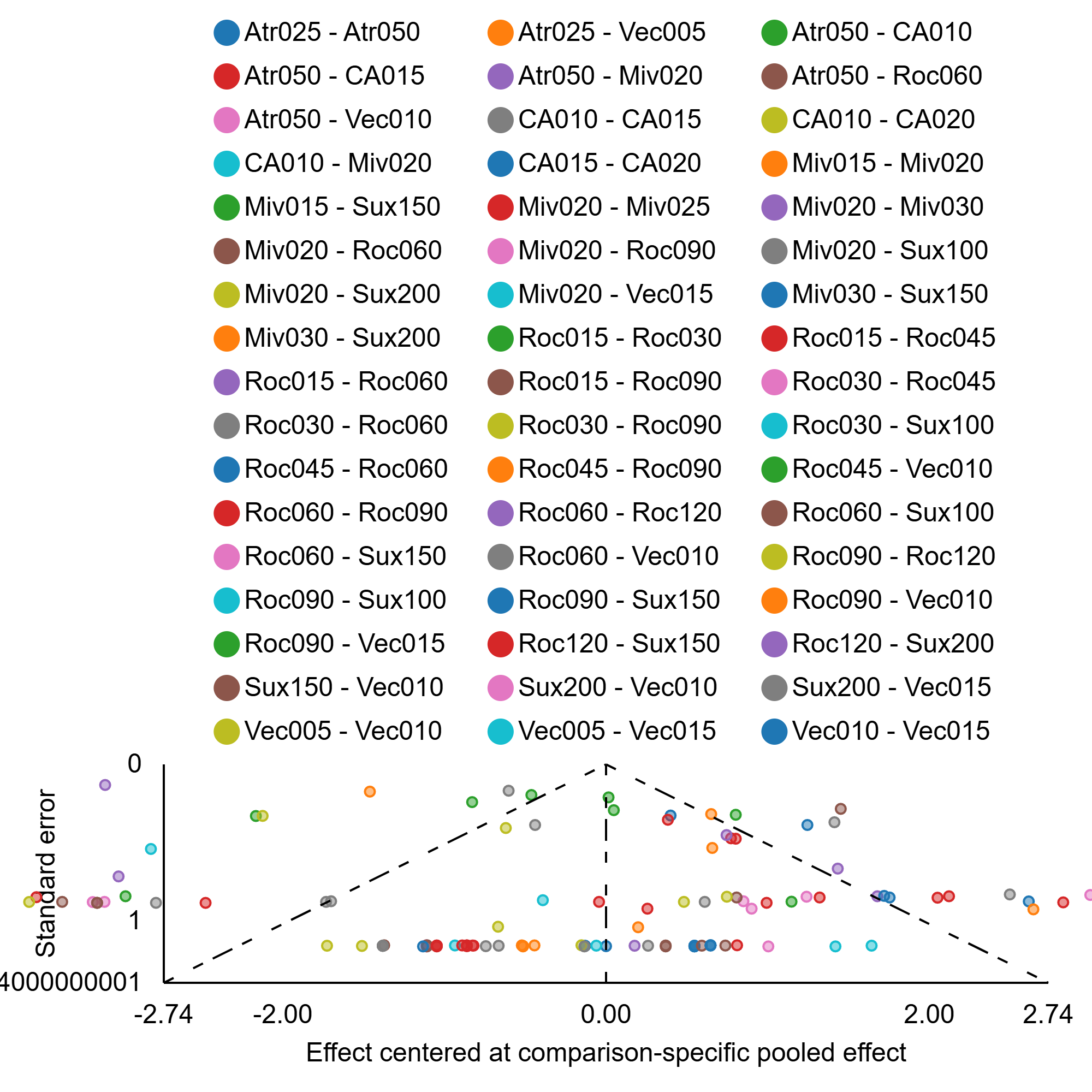


1. **Funnelplot Network analysis for Excellent intubation Conditions**


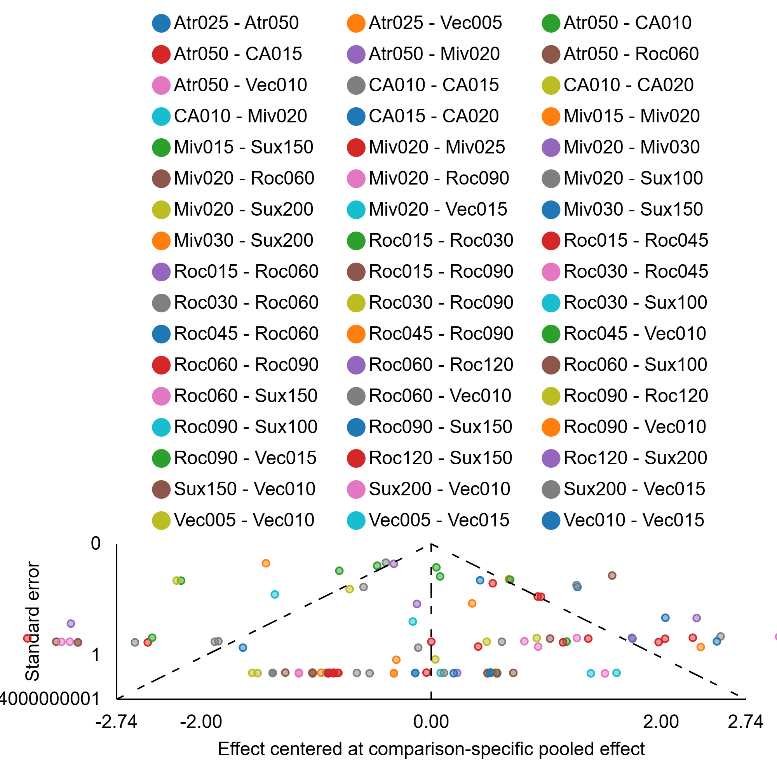


**Figure S6.** Funnel plots assessing publication bias for excellent (EIC, upper panel) and acceptable (AIC, lower panel) intubation conditions. Effect sizes are plotted against study precision (inverse of the standard error). The vertical line represents no intervention effect, and the dashed lines indicate the 95% confidence region. Symmetry suggests the absence of publication bias or small-study effects, while asymmetry may indicate their presence. Egger’s test showed no significant asymmetry for EIC (p = 0.1908) or AIC (p = 0.0561).^[[1]](#footnote-1),^^[[2]](#footnote-2),^^[[3]](#footnote-3)^

1. Sterne JA, Sutton AJ, Ioannidis JP, et al. Recommendations for examining and interpreting funnel plot asymmetry in meta-analyses of randomised controlled trials. BMJ. 2011;343:d4002. [↑](#footnote-ref-1)
2. Egger M, Davey Smith G, Schneider M, Minder C. Bias in meta-analysis detected by a simple, graphical test. BMJ. 1997 Sep 13;315(7109):629-34. [↑](#footnote-ref-2)
3. Lin L, Chu H. Quantifying publication bias in meta-analysis. Biometrics. 2018 Sep;74(3):785-794. [↑](#footnote-ref-3)
