# Supplementary material for: Effect of neuromuscular blocking agents on tracheal intubation quality in paediatric patients: a systematic review using network meta-analysis and meta-regression
Source: Br J Anaesth. 2025 Sep 3;135(6):1787–802. doi: 10.1016/j.bja.2025.08.036 (PMC12799451; doi:10.1016/j.bja.2025.08.036)
Supplement: Multimedia Component 8 [file mmc8.docx]

**Supplementary material File 8.:** **Ranking**


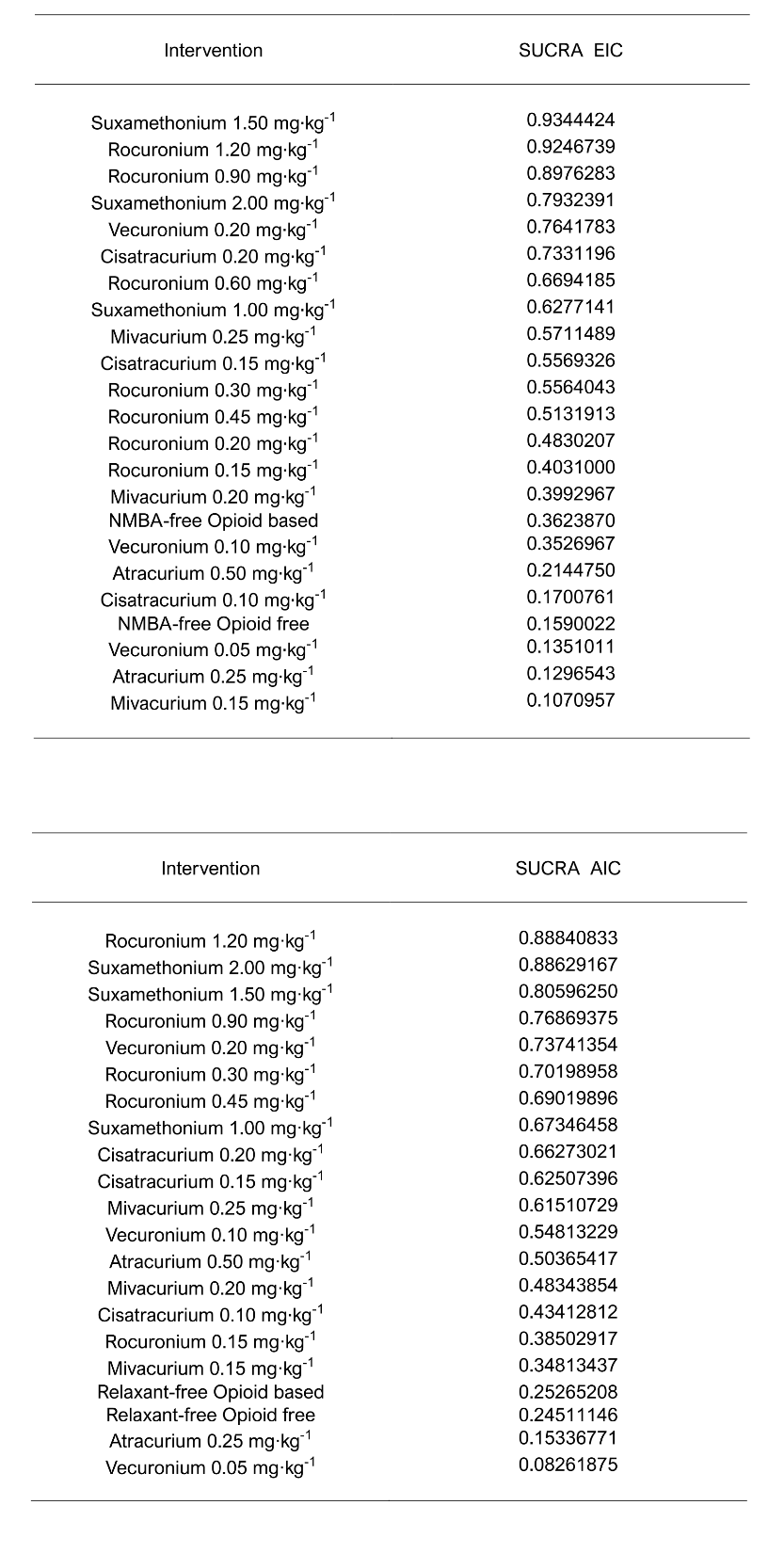


**Table S3**. Comparative effectiveness research using network meta-analysis can establish a classification of competing treatments, ranging from the most to the least preferable option. The tables display the hierarchy of interventions used to facilitate airway management in paediatric patients, ranked according to their Surface Under the Cumulative Ranking (SUCRA) values, which range from 0 to 1. Higher SUCRA values (closer to 1) indicate greater effectiveness, while lower values (closer to 0) suggest reduced benefit. The upper panel shows the ordering for excellent intubation conditions (EIC), while the lower panel presents the ordering for acceptable intubation conditions (AIC).^[[1]](#footnote-1)^

1. Salanti G, Nikolakopoulou A, Efthimiou O, Mavridis D, Egger M, White IR. Introducing the Treatment Hierarchy Question in Network Meta-Analysis. Am J Epidemiol. 2022 Mar 24;191(5):930-938. [↑](#footnote-ref-1)
