# Supplementary material for: Effect of neuromuscular blocking agents on tracheal intubation quality in paediatric patients: a systematic review using network meta-analysis and meta-regression
Source: Br J Anaesth. 2025 Sep 3;135(6):1787–802. doi: 10.1016/j.bja.2025.08.036 (PMC12799451; doi:10.1016/j.bja.2025.08.036)
Supplement: Multimedia Component 9 [file mmc9.docx]

**Supplementary material File 9.: Times to intubation**

| NMBA treatment | Time to intubation (s)  (Median [IQR]) |
| --- | --- |
|  |  |
| Atr 0.25 | 129.6 [86.8, 160.1] |
| Atr 0.50 | 131.1 [121.0, 140.9] |
| CA 0.10 | 172.9 [157.6, 187.3] |
| CA 0.15 | 132.6 [119.6, 144.6] |
| CA 0.20 | 118.9 [98.9, 136.0] |
| Miv 0.15 | 123.1 [97.5, 145.3] |
| Miv 0.20 | 132.0 [125.0, 138.8] |
| Miv 0.25 | 129.3 [119.0, 139.2] |
| Roc 0.15 | 106.1 [99.6, 112.5] |
| Roc 0.30 | 92.6 [87.0, 98.0] |
| Roc 0.45 | 88.7 [79.5, 97.9] |
| Roc 0.60 | 79.7 [75.5, 83.9] |
| Roc 0.90 | 69.9 [63.7, 76.0] |
| Roc 1.20 | 64.8 [44.7, 83.7] |
| Sux 1.00 | 57.3 [56.1, 58.4] |
| Sux 1.50 | 65.0 [60.2, 69.8] |
| Sux 2.00 | 66.4 [58.1, 74.5] |
| Vec 0.05 | 157.5 [141.4, 170.8] |
| Vec 0.10 | 134.6 [124.8, 144.1] |
| Vec 0.20 | 83.3 [76.2, 90.3] |
|  |  |

**Table S4.** Median [IQR] of intubation times across study arms, stratified by the neuromuscular blocking agent (NMBA) used and its administered dose (mg∙kg⁻¹). The NMBAs assessed include: atracurium (Atr), cisatracurium (CA), mivacurium (Miv), rocuronium (Roc), succinylcholine (Sux), and vecuronium (Vec).

|  | **Atr0.25** | **Atr0.50** | **CA0.10** | **CA0.15** | **CA0.20** | **Miv0.15** | **Miv0.20** | **Miv0.25** | **Roc0.15** | **Roc0.30** | **Roc0.45** | **Roc0.60** | **Roc0.90** | **Roc1.20** | **Sux1.00** | **Sux1.50** | **Sux2.00** | **Vec0.05** | **Vec0.10** | **Vec0.20** |
| --- | --- | --- | --- | --- | --- | --- | --- | --- | --- | --- | --- | --- | --- | --- | --- | --- | --- | --- | --- | --- |
|  |  |  |  |  |  |  |  |  |  |  |  |  |  |  |  |  |  |  |  |  |
| **Atr0.25** | 0.500 | 0.483 | 0.197 | 0.476 | 0.568 | 0.532 | 0.480 | 0.497 | 0.655 | 0.725 | 0.738 | 0.778 | 0.810 | 0.808 | 0.849 | 0.827 | 0.820 | 0.310 | 0.458 | 0.763 |
| **Atr0.50** | 0.517 | 0.500 | 0.074 | 0.479 | 0.659 | 0.587 | 0.479 | 0.533 | 0.911 | 0.982 | 0.975 | 0.998 | 0.999 | 0.971 | 1.000 | 1.000 | 0.998 | 0.196 | 0.434 | 0.990 |
| **CA0.10** | 0.803 | 0.926 | 0.500 | 0.903 | 0.929 | 0.884 | 0.935 | 0.931 | 0.988 | 0.995 | 0.995 | 0.998 | 0.999 | 0.994 | 1.000 | 0.999 | 0.999 | 0.693 | 0.910 | 0.997 |
| **CA0.15** | 0.524 | 0.521 | 0.097 | 0.500 | 0.662 | 0.593 | 0.508 | 0.550 | 0.874 | 0.952 | 0.950 | 0.982 | 0.989 | 0.961 | 0.996 | 0.993 | 0.990 | 0.224 | 0.464 | 0.970 |
| **CA0.20** | 0.432 | 0.341 | 0.071 | 0.338 | 0.500 | 0.460 | 0.317 | 0.363 | 0.659 | 0.790 | 0.803 | 0.871 | 0.906 | 0.875 | 0.941 | 0.922 | 0.912 | 0.164 | 0.298 | 0.843 |
| **Miv0.15** | 0.468 | 0.413 | 0.116 | 0.407 | 0.540 | 0.500 | 0.399 | 0.432 | 0.670 | 0.777 | 0.791 | 0.851 | 0.887 | 0.866 | 0.927 | 0.905 | 0.896 | 0.223 | 0.377 | 0.827 |
| **Miv0.20** | 0.520 | 0.521 | 0.065 | 0.492 | 0.683 | 0.601 | 0.500 | 0.558 | 0.960 | 0.997 | 0.990 | 1.000 | 1.000 | 0.978 | 1.000 | 1.000 | 1.000 | 0.184 | 0.443 | 0.997 |
| **Miv0.25** | 0.503 | 0.467 | 0.069 | 0.450 | 0.637 | 0.568 | 0.442 | 0.500 | 0.890 | 0.973 | 0.966 | 0.995 | 0.997 | 0.966 | 1.000 | 0.999 | 0.997 | 0.183 | 0.402 | 0.985 |
| **Roc0.15** | 0.345 | 0.089 | 0.012 | 0.126 | 0.341 | 0.330 | 0.040 | 0.110 | 0.500 | 0.845 | 0.840 | 0.977 | 0.990 | 0.906 | 0.999 | 0.996 | 0.987 | 0.067 | 0.060 | 0.927 |
| **Roc0.30** | 0.275 | 0.018 | 0.005 | 0.048 | 0.210 | 0.223 | 0.003 | 0.027 | 0.155 | 0.500 | 0.592 | 0.883 | 0.959 | 0.823 | 0.999 | 0.988 | 0.952 | 0.042 | 0.011 | 0.748 |
| **Roc0.45** | 0.262 | 0.025 | 0.005 | 0.050 | 0.197 | 0.209 | 0.010 | 0.034 | 0.160 | 0.408 | 0.500 | 0.722 | 0.864 | 0.771 | 0.977 | 0.925 | 0.878 | 0.041 | 0.017 | 0.621 |
| **Roc0.60** | 0.222 | 0.002 | 0.002 | 0.018 | 0.129 | 0.149 | 0.000 | 0.005 | 0.023 | 0.117 | 0.278 | 0.500 | 0.812 | 0.698 | 0.999 | 0.934 | 0.833 | 0.028 | 0.002 | 0.388 |
| **Roc0.90** | 0.190 | 0.001 | 0.001 | 0.011 | 0.094 | 0.113 | 0.000 | 0.003 | 0.010 | 0.041 | 0.136 | 0.188 | 0.500 | 0.569 | 0.908 | 0.662 | 0.592 | 0.022 | 0.001 | 0.181 |
| **Roc1.20** | 0.192 | 0.029 | 0.006 | 0.039 | 0.125 | 0.134 | 0.022 | 0.034 | 0.094 | 0.177 | 0.229 | 0.302 | 0.431 | 0.500 | 0.606 | 0.498 | 0.480 | 0.031 | 0.024 | 0.276 |
| **Sux1.00** | 0.151 | 0.000 | 0.001 | 0.004 | 0.059 | 0.073 | 0.000 | 0.000 | 0.001 | 0.001 | 0.023 | 0.001 | 0.092 | 0.394 | 0.500 | 0.152 | 0.232 | 0.015 | 0.000 | 0.025 |
| **Sux1.50** | 0.173 | 0.000 | 0.001 | 0.007 | 0.078 | 0.095 | 0.000 | 0.001 | 0.004 | 0.012 | 0.075 | 0.066 | 0.338 | 0.502 | 0.848 | 0.500 | 0.462 | 0.019 | 0.000 | 0.094 |
| **Sux2.00** | 0.180 | 0.002 | 0.001 | 0.010 | 0.088 | 0.104 | 0.000 | 0.003 | 0.013 | 0.048 | 0.122 | 0.167 | 0.408 | 0.520 | 0.768 | 0.538 | 0.500 | 0.020 | 0.001 | 0.161 |
| **Vec0.05** | 0.690 | 0.804 | 0.307 | 0.776 | 0.836 | 0.777 | 0.816 | 0.817 | 0.933 | 0.958 | 0.959 | 0.972 | 0.978 | 0.969 | 0.985 | 0.981 | 0.980 | 0.500 | 0.778 | 0.967 |
| **Vec0.10** | 0.542 | 0.566 | 0.090 | 0.536 | 0.702 | 0.623 | 0.557 | 0.598 | 0.940 | 0.989 | 0.983 | 0.998 | 0.999 | 0.976 | 1.000 | 1.000 | 0.999 | 0.222 | 0.500 | 0.993 |
| **Vec0.20** | 0.237 | 0.010 | 0.003 | 0.030 | 0.157 | 0.173 | 0.003 | 0.015 | 0.074 | 0.252 | 0.379 | 0.612 | 0.819 | 0.724 | 0.975 | 0.906 | 0.839 | 0.033 | 0.007 | 0.500 |
|  |  |  |  |  |  |  |  |  |  |  |  |  |  |  |  |  |  |  |  |  |

**Table S5.** Bayesian pairwise post-hoc comparison results for intubation times across different NMBA treatments. Each value represents the probability that the treatment listed in the row has a higher mean intubation time than the treatment in the column. A value close to 1 indicates strong evidence that the row group has a longer average intubation time; values near 0.5 suggest no meaningful difference; and values close to 0 indicate that the row group has a shorter average intubation time than the column group.
