# Supplementary material for: Effect of neuromuscular blocking agents on tracheal intubation quality in paediatric patients: a systematic review using network meta-analysis and meta-regression
Source: Br J Anaesth. 2025 Sep 3;135(6):1787–802. doi: 10.1016/j.bja.2025.08.036 (PMC12799451; doi:10.1016/j.bja.2025.08.036)
Supplement: Multimedia Component 10 [file mmc10.docx]

**Supplementary material File 10.: Subgroup analysis of factors affecting intubation quality with NMBAs.**


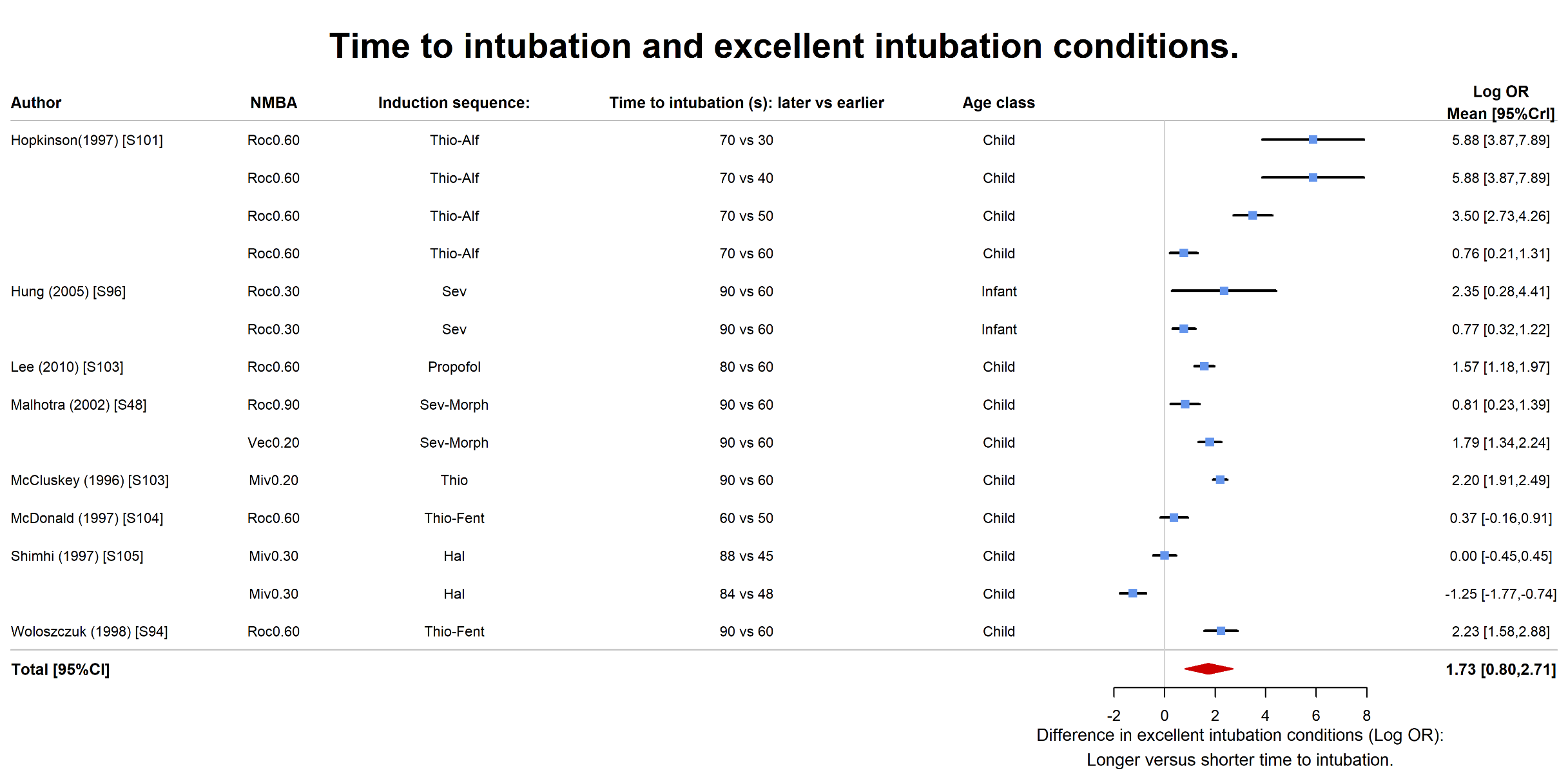


**Table S6.** A pairwise meta-analysis estimating the impact of longer intubation times on the likelihood of achieving excellent intubation conditions. logOR of 1.73 [0.80, 2.71] or OR of 5.64 [2.23, 15.03] (mean [95% CrI]).


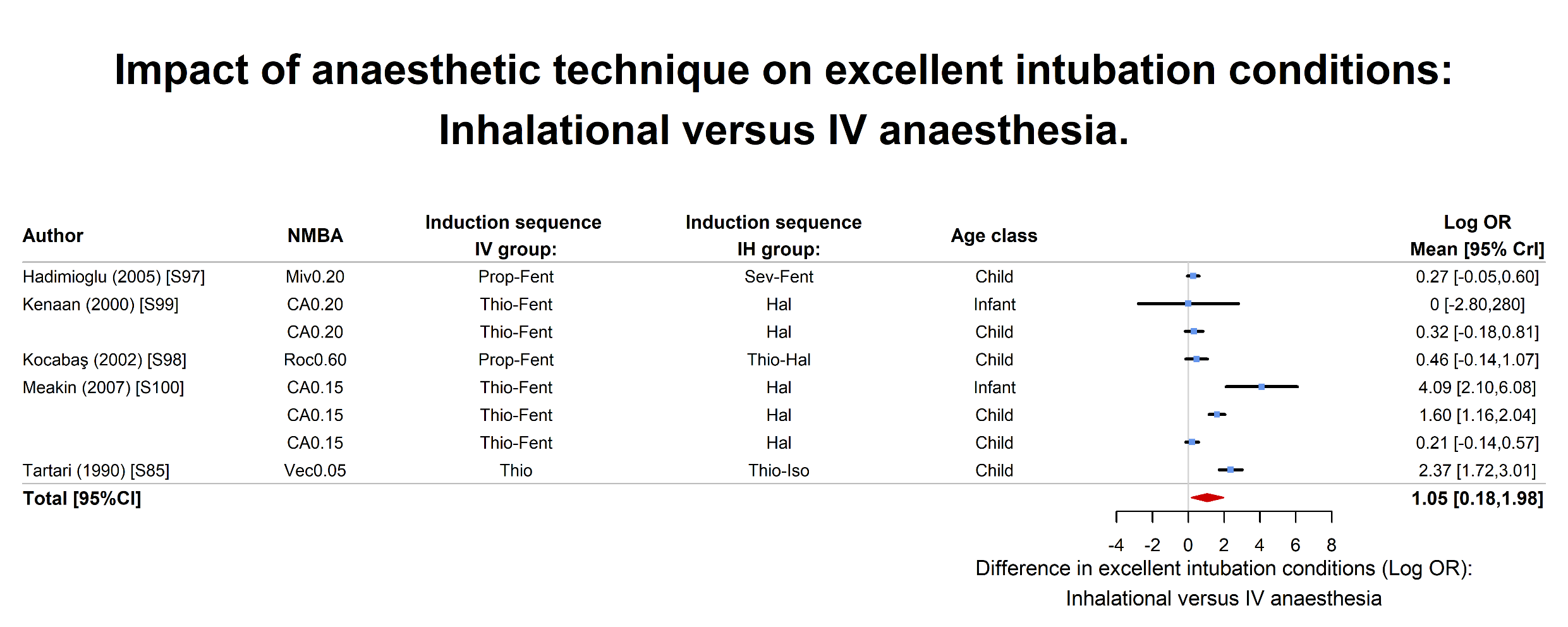


**Table S7**. A pairwise meta-analysis estimating the impact of inhalational anaesthetics on achieving excellent intubation conditions. logOR: 1.05 [0.18,1.98] corresponding to OR: 2.86 [1.20, 7.24] (mean[95%CrI]).


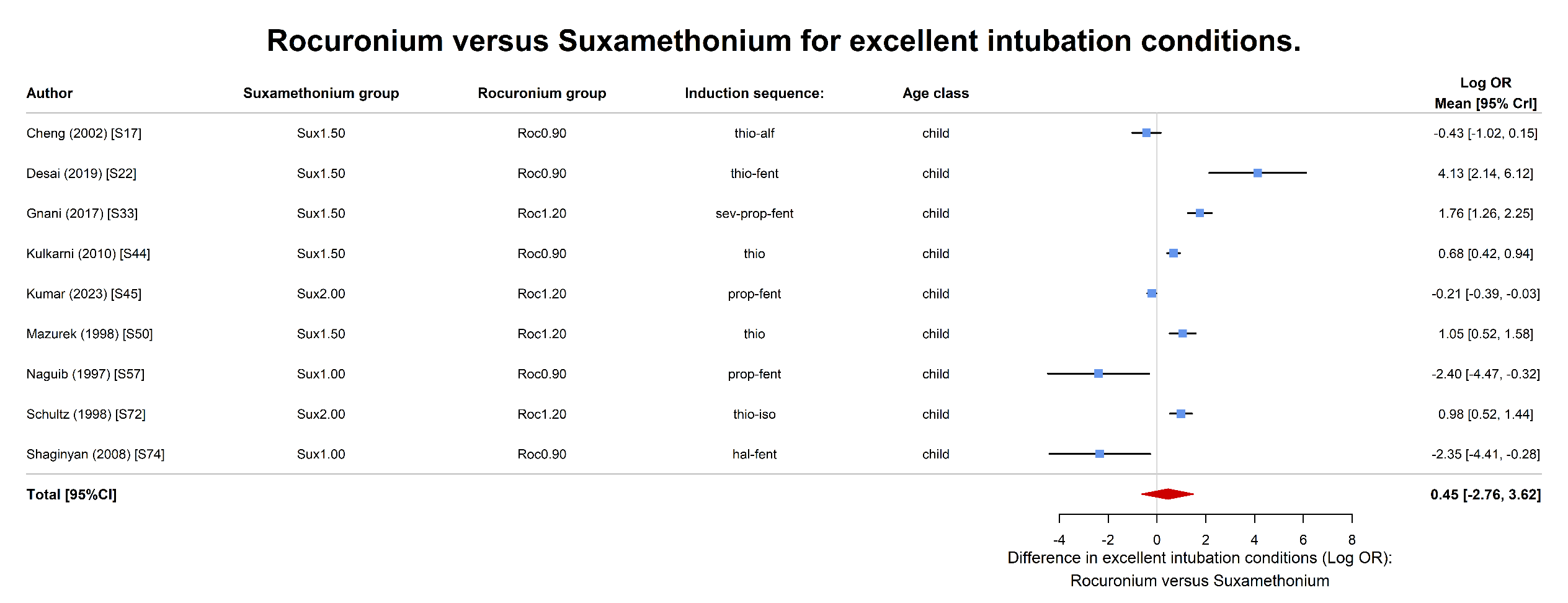


**Table S8.** Pairwise meta-analysis comparing the effect of Rocuronium ≥0.90 mg∙kg^-1^ versus Suxamethonium 1-2 mg∙kg^-1^ on the creation of excellent intubation conditions. LogOR: 0.45 [-2.76,3.62] corresponding to OR=1.57 [0.06, 37.34] (mean[95%CrI]).
