# Supplementary material for: Effect of neuromuscular blocking agents on tracheal intubation quality in paediatric patients: a systematic review using network meta-analysis and meta-regression
Source: Br J Anaesth. 2025 Sep 3;135(6):1787–802. doi: 10.1016/j.bja.2025.08.036 (PMC12799451; doi:10.1016/j.bja.2025.08.036)
Supplement: Multimedia Component 11 [file mmc11.docx]

**Supplementary material File 11.: Outcomes associated with the use or avoidance of NMBAs**


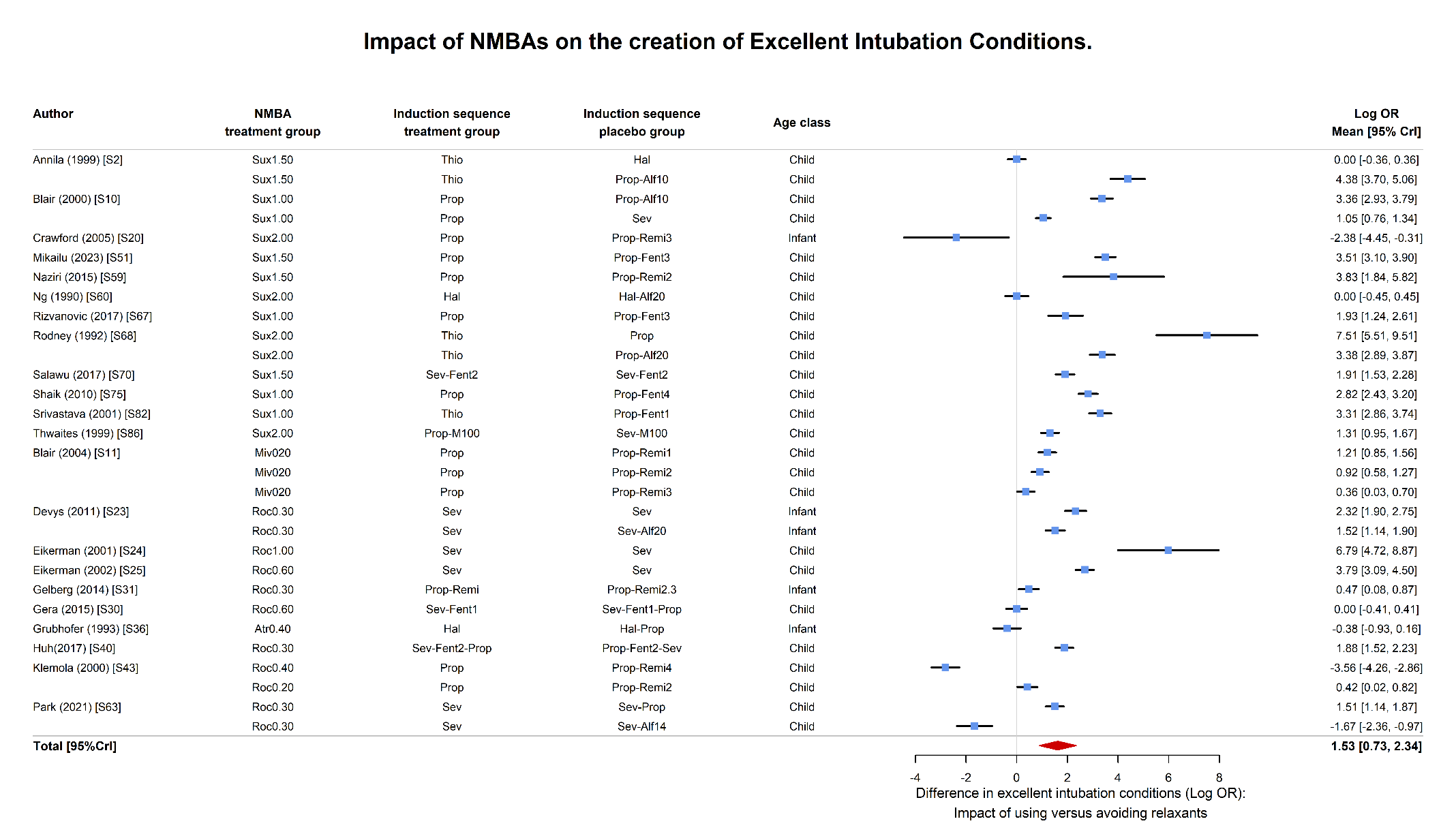


**Table S9.** A pairwise meta-analysis estimating the effect of using NMBAs versus avoiding them on the likelihood of achieving excellent intubation conditions. Log OR: 1.62 [0.89, 2.35], corresponding to an odds ratio of 5.05 [2.43,10.48] (mean [95% CrI]).


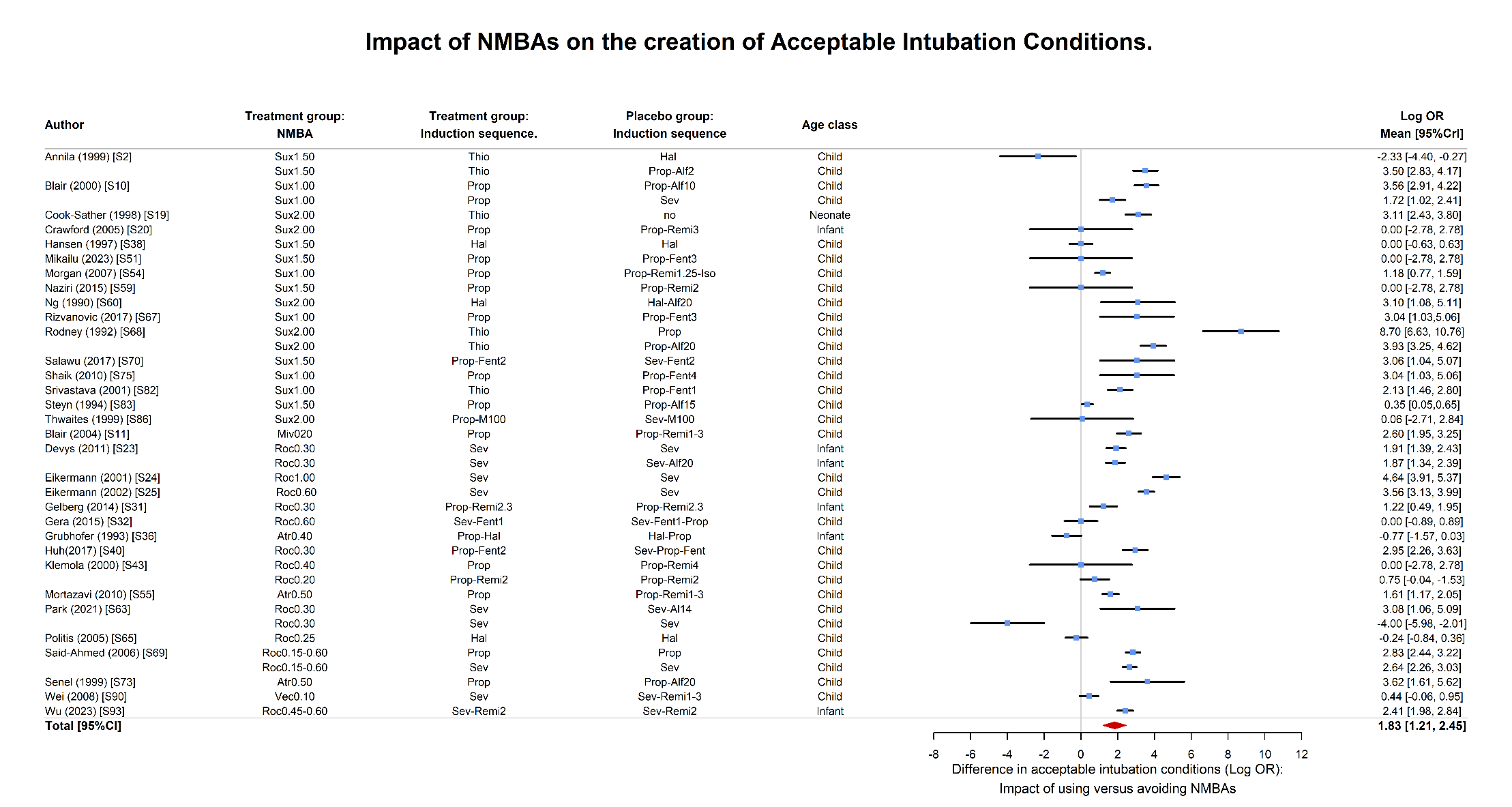


**Table S10.** A pairwise meta-analysis estimating the effect of using NMBAs versus avoiding them on the likelihood of achieving acceptable intubation conditions. Log OR: 1.83 [1.21, 2.45], equivalent to an odds ratio of 6.23 [3.35, 11.58] (mean [95%CrI]).


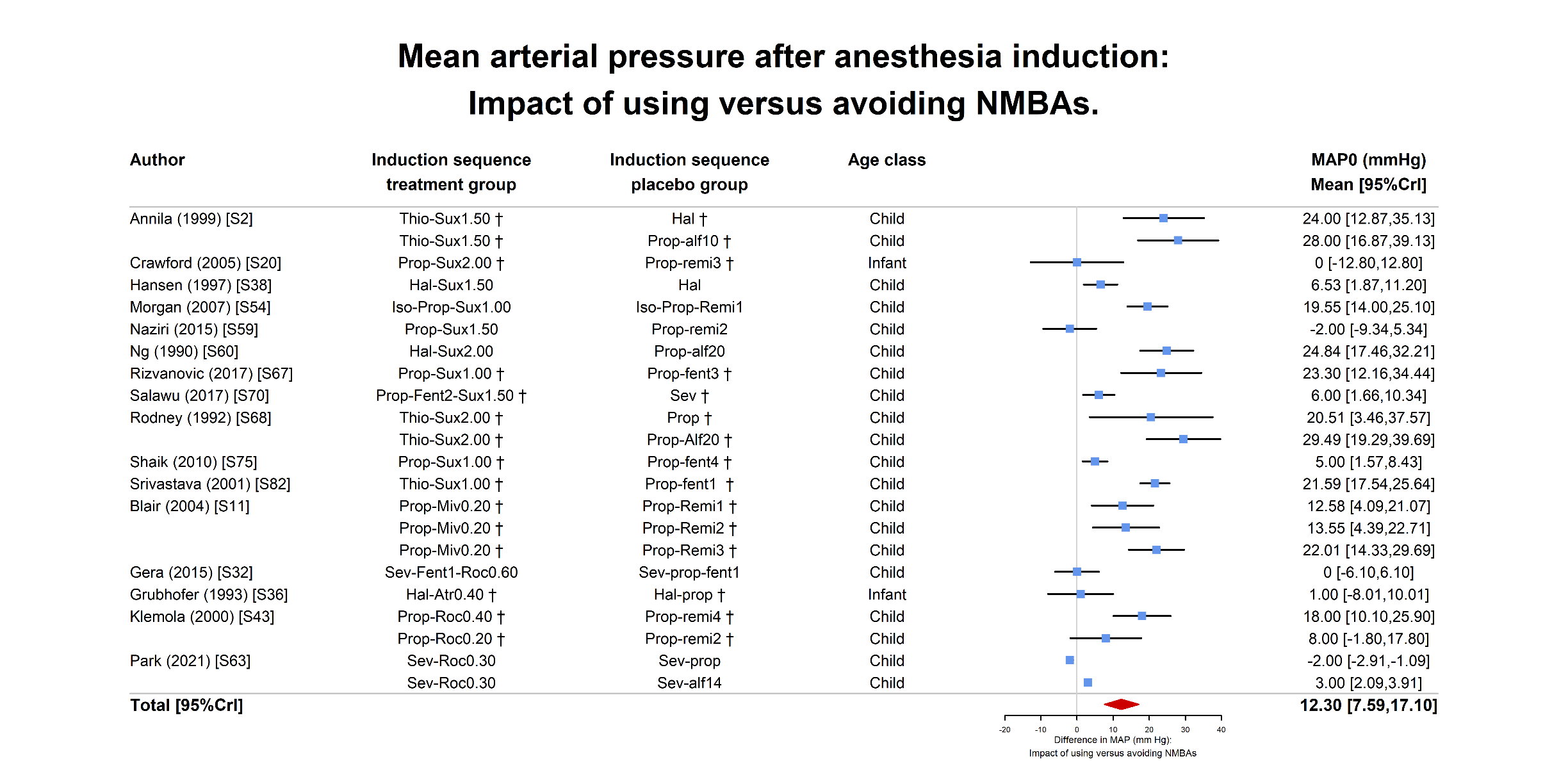


**Table S11.** Effect of anaesthesia induction techniques involving NMBAs versus those avoiding them on mean arterial pressure (MAP). Values are mean difference [95%CrI] mmHg. †: indicates use of an anticholinergic, atropine or glycopyrrolate during induction.


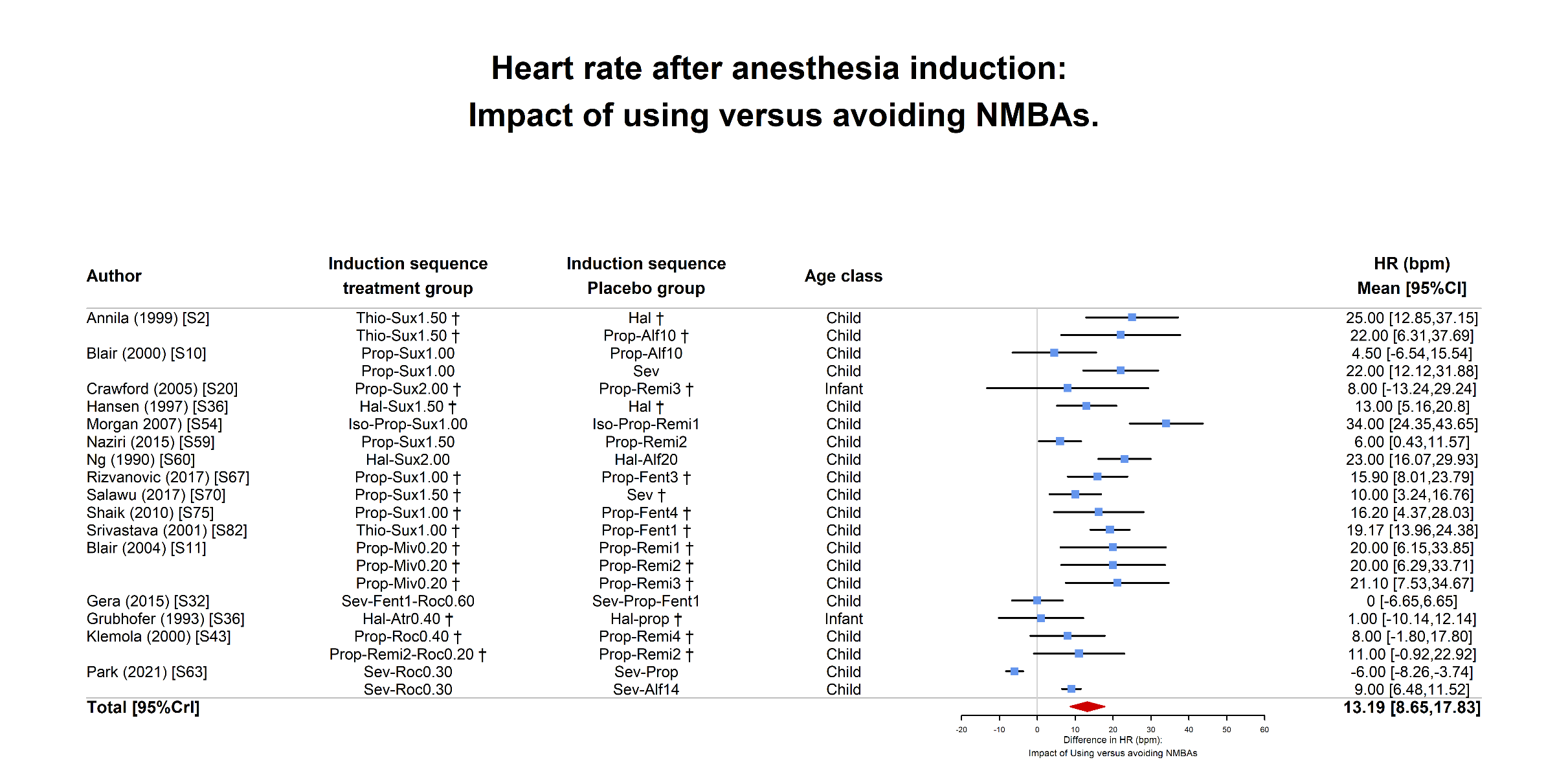


**Table S12.** Effect of anaesthesia induction techniques involving NMBAs versus those avoiding them on heart rate [HR]. Values are mean difference [95%CrI] beats per minute (bpm). †Indicates use of an anticholinergic agent, such as atropine or glycopyrrolate during induction.
