# Supplementary figures and images for: Effect of neuromuscular blocking agents on tracheal intubation quality in paediatric patients: a systematic review using network meta-analysis and meta-regression
Source: Br J Anaesth. 2025 Sep 3;135(6):1787–802. doi: 10.1016/j.bja.2025.08.036 (PMC12799451; doi:10.1016/j.bja.2025.08.036)

**Supplementary material File 12: Trial sequential analysis.**


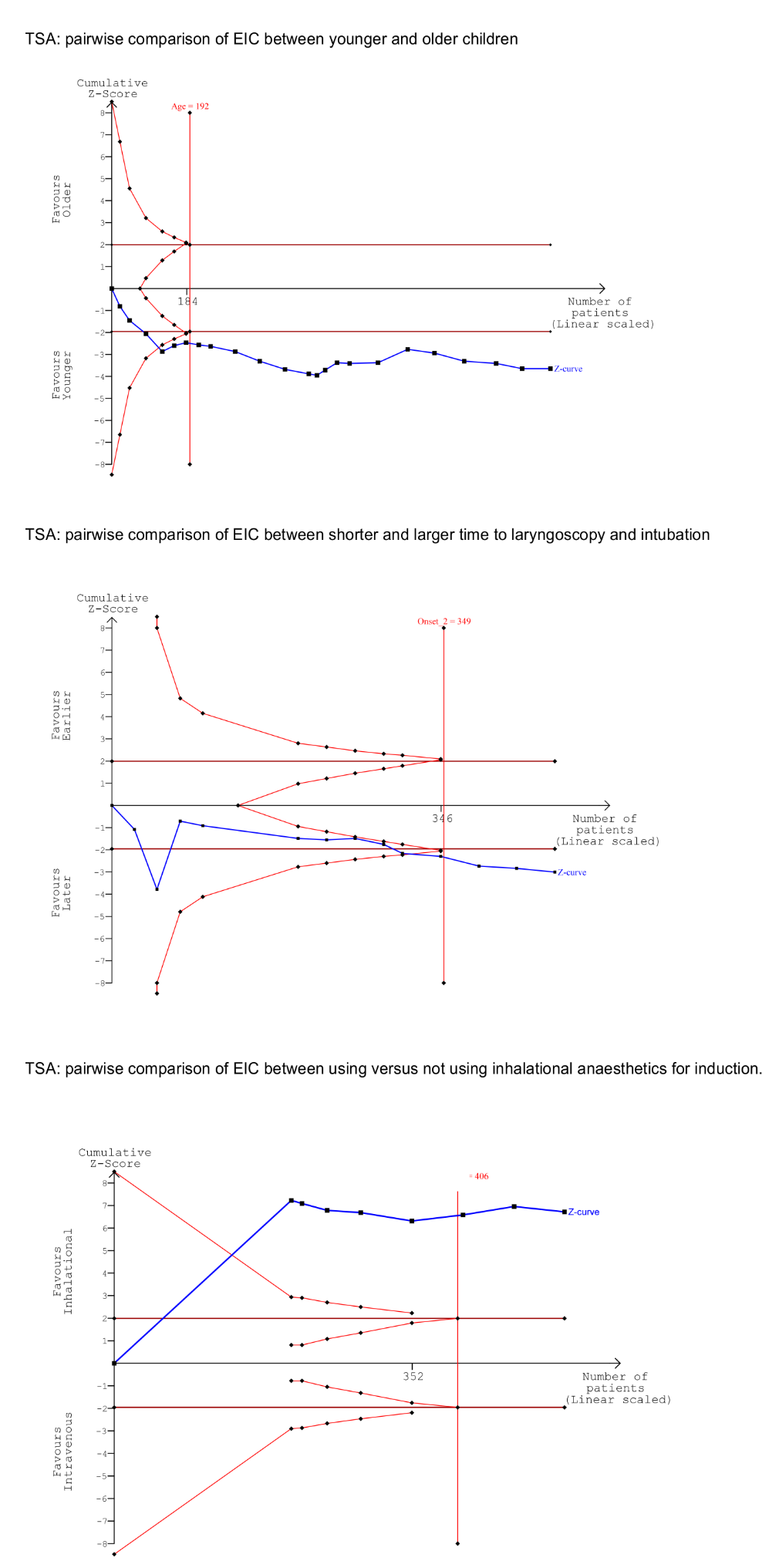


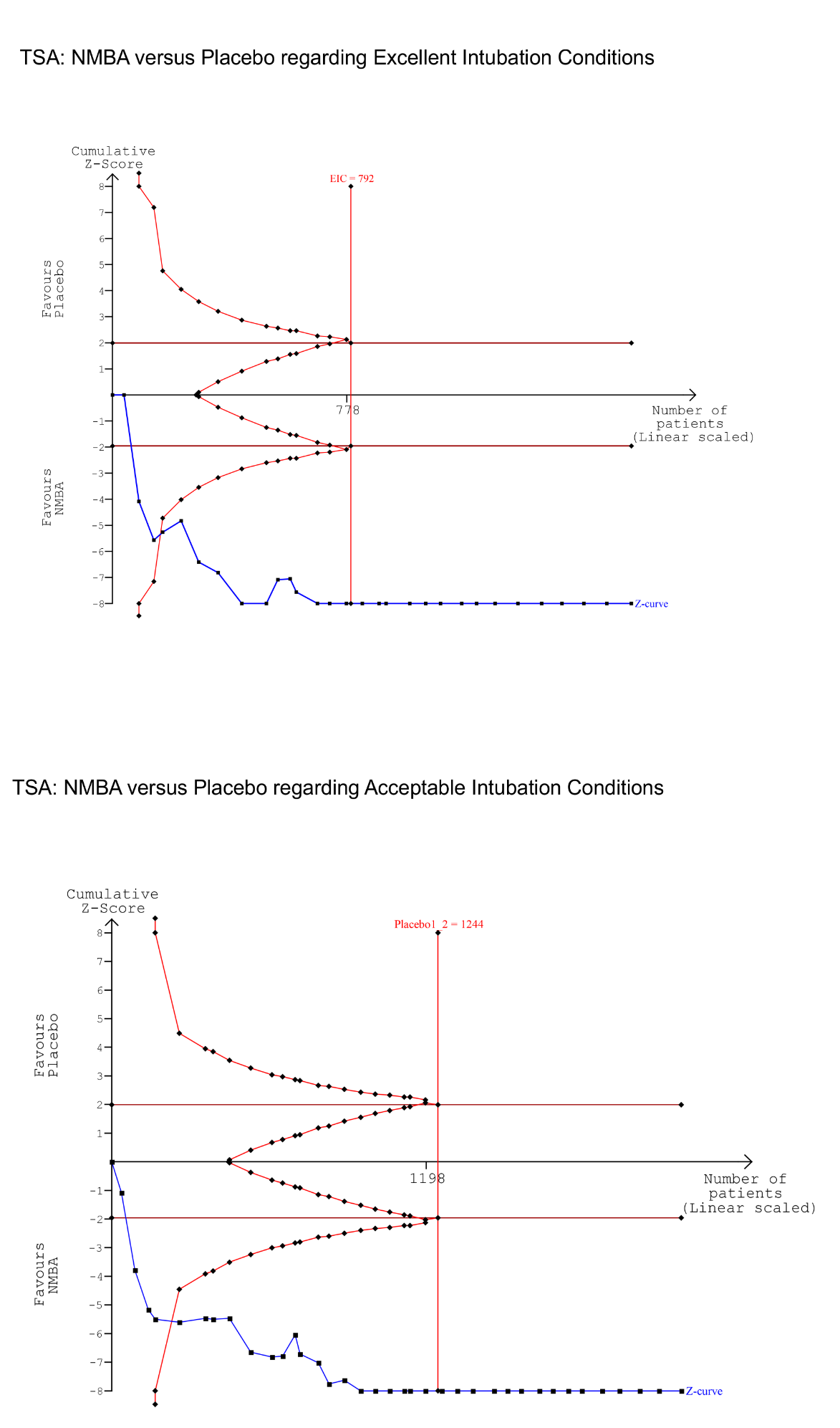


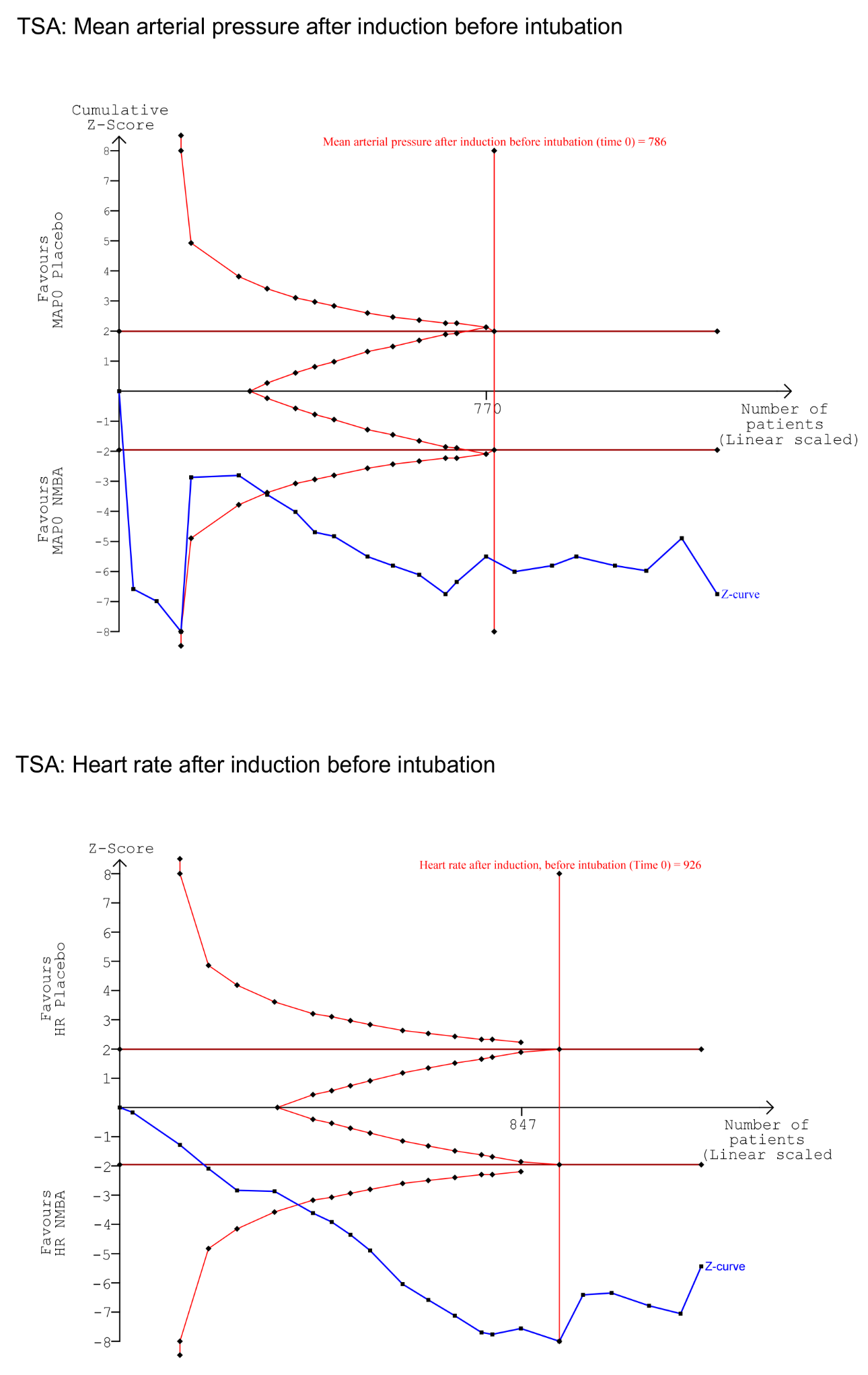

Supplement: Multimedia Component 12 [file mmc12.docx]
