# Supplementary material for: Effect of neuromuscular blocking agents on tracheal intubation quality in paediatric patients: a systematic review using network meta-analysis and meta-regression
Source: Br J Anaesth. 2025 Sep 3;135(6):1787–802. doi: 10.1016/j.bja.2025.08.036 (PMC12799451; doi:10.1016/j.bja.2025.08.036)
Supplement: Multimedia Component 13 [file mmc13.docx]

**Supplementary material File 13.:**

**Metaregression of factors associated with the quality of intubation.**

1. **Linear random effects metaregression model^^[[1]](#footnote-1)^,^[[2]](#footnote-2)^^**

ΔYi ~ β_0_ + β_1_ ∙ X_1i_ + β_2_∙X_2i_ + … + β_n_ ∙ X_ni_ + ε_i_ + ϛ

In which:

| ΔYi | difference in trial i regarding   - logOR of EIC or AIC (ΔlogOR) - mean arterial pressure (ΔMAP) or heart rate (ΔHR) |
| --- | --- |
| β_0_, β_1_, β_2_, β_3_ | coefficients to be estimated; |
| X_1i_, X_2i_, … , X_ni_, | covariables (prognostic variables or moderators) in trial i to be tested, eg.   - age (yr) or ln(age); - use of suxamethonium (yes/no or 1/0); - use of opioids (yes/no or 1/0); - use of anticholinergic agents (yes/no or 1/0); |
| ε_i_ | variation within trial i: sampling error |
| Ϛ | across trial variation N(0,τ²): heterogeneity |

1. **Exploring factors associated with intubation quality.**

| Parameter | Δ Intubation quality (logOR):  NMBA facilitated versus relaxant-free | |
| --- | --- | --- |
|  | Coefficients parameters  meta-regression model EIC | Coefficients parameters (AIC)  meta-regression model AIC |
|  |  |  |
| Age (years) | 0.34 [0.20,0.49] * | 0.36 [0.22,0.50] * |
| Suxamethonium (1/0) | 1.47 [0.64,2.30] * | 1.24 [0.40,2.10] * |
| Opioids in the NMBA-free group (1/0) | -2.61 [-2.57,-0.67] * | -1.04 [-2.00,-0.11] * |
| Opioids in the NMBA group (1/0) | 0.59 [-0.54,1.74] ns | -0.36 [-1.53,0.81] ns |
| tau (τ) | 2.12 [1.85,2.45] * | 1.84 [1.56,2.20] * |
|  |  |  |

**Table S13.** Meta-regression of factors associated with intubation quality. The meta-regression analysis indicated that both age and the use of suxamethonium were associated with greater differences in intubation quality (ΔLogOR for EIC and AIC) between protocols that included NMBAs and those that did not. Conversely, adding opioids to the NMBA-free (placebo) group appeared to attenuate these differences. However, adding opioids to the NMBA group had no significant effect on intubation quality. tau(τ): standard deviation of random effect; *: significant; ns: not significant.

1. **Exploring factors associated with haemodynamics.**

| Parameter | Δ Haemodynamics:  NMBA facilitated versus relaxant-free | |
| --- | --- | --- |
|  | ΔMAP (mm Hg) | ΔHR (bpm) |
|  |  |  |
| Anticholinergic (1/0) | 6.98 [3.67,10.34]* | 4.81 [1.38,8.25]* |
| Suxamethonium (1/0) | 6.23[2.88, 9.58]* | 8.69 [5.08,12.30]* |
| Opioids in the NMBA-free group (1/0) | 6.98 [3.67,10.34]* | 7.75 [4.28,11.24]* |
| Opioids in the NMBA group (1/0) | -3.32[-9.98,-3.30]* | -6.36 [-13.27,-0.56]* |
| tau (τ) | 7.38 [6.03,9.02] * | 6.03 [4.44,8.02] * |
|  |  |  |

**Table S14.** Meta-regression analysis of factors associated with haemodynamic responses during anaesthesia induction prior to tracheal intubation. The meta-regression analysis suggests that opioids were associated with a reduction in mean arterial pressure (MAP) and heart rate (HR), while both anticholinergics and suxamethonium induced transient increase in HR and MAP, particularly within the first few minutes of administration. tau(τ): standard deviation of random effect; *: significant; ns: not significant.

1. DerSimonian R, Laird N. Meta-analysis in clinical trials revisited. Contemp Clin Trials. 2015 Nov;45(Pt A):139-45. doi: 10.1016/j.cct.2015.09.002. [↑](#footnote-ref-1)
2. Röver C. Bayesian random-effects meta-analysis using the Bayesmeta R package. J Stat Softw. 2020; 93(6): 1-51. [↑](#footnote-ref-2)
