# Supplementary material for: Effect of neuromuscular blocking agents on tracheal intubation quality in paediatric patients: a systematic review using network meta-analysis and meta-regression
Source: Br J Anaesth. 2025 Sep 3;135(6):1787–802. doi: 10.1016/j.bja.2025.08.036 (PMC12799451; doi:10.1016/j.bja.2025.08.036)
Supplement: Multimedia Component 14 [file mmc14.docx]

**Supplementary material File 14.:**

**Confidence In Network Meta-Analysis (CiNeMA)^^[[1]](#footnote-1)^^.**

**
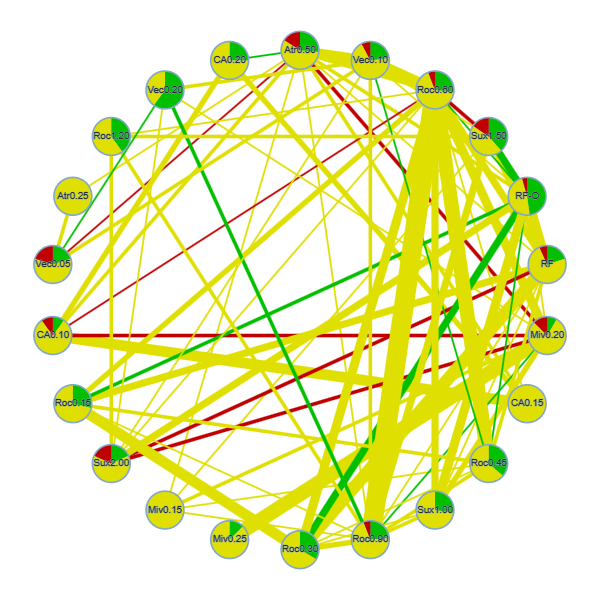
**

**Figure S7.** A network plot of treatments used to facilitate tracheal intubation, including neuromuscular blocking agents (NMBAs) and NMBA-free interventions. The plot illustrates the connections within the treatment comparison network. Edge widths are proportional to the number of study arms included in each comparison. The colours of the edges and nodes refer to the risk of bias: low (green), moderate (yellow) and high (red).


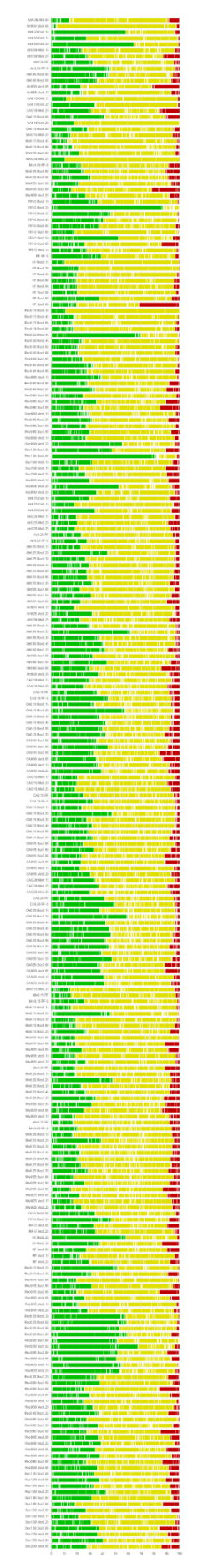


**Figure S8.** A bar chart displaying the risk of bias in treatment comparisons for facilitating tracheal intubation. Each bar represents a comparison from the network shown in Figure S7 and indicates the percentage contribution of studies rated as having low (green), moderate (yellow), or high (red) risk of bias.

1. Nikolakopoulou A, Higgins JPT, Papakonstantinou T, Chaimani A, Del Giovane C, Egger M, Salanti G. CINeMA: An approach for assessing confidence in the results of a network meta-analysis. PLoS Med. 2020;17:e1003082. doi: 10.1371/journal.pmed.1003082. [↑](#footnote-ref-1)
